# A Dutch paediatric palliative care guideline: a systematic review and evidence-based recommendations for symptom treatment

**DOI:** 10.1186/s12904-024-01367-w

**Published:** 2024-03-13

**Authors:** Kim C. van Teunenbroek, Renée L. Mulder, Inge M. L. Ahout, Karen G. C. B. Bindels-de Heus, Catharina M. Delsman-van Gelder, Annemie F. S. Galimont-Collen, Marinka A. R. de Groot, Katja M. J. Heitink-Polle, Jeffry Looijestijn, Maarten O. Mensink, Selma Mulder, Jolanda H. Schieving, Antoinette Y. N. Schouten-van Meeteren, Johannes M. A. Verheijden, Hester Rippen, Brigitt C. M. Borggreve, Leontien C. M. Kremer, A. A. Eduard Verhagen, Erna M. C. Michiels, on behalf of the working groups symptom treatment and refractory symptom treatment of the Dutch paediatric palliative care guideline, Mattijs W. Alsem, Esther M. M. van den Bergh, Govert Brinkhorst, Arno Colenbrander, Linda Corel, Jennifer van Dijk, Laurent Favié, Karin Geleijns, Saskia J. Gischler, Lisette ‘t Hart-Kerkhoffs, Hanneke Heinen, Cindy Joosen, Carla C. M. Juffermans, Hennie Knoester, Barbara de Koning, Tom de Leeuw, Hilda Mekelenkamp, Mariska P. Nieuweboer, Sebastianus B. J. Oude Ophuis, Suzanne G. M. A. Pasmans, Elise M. van de Putte, Emmy Räkers, Irma M. Rigter, Christel D. Rohrich, Elisabeth J. Ruijgrok, Kim van der Schoot, Ellen Siegers-Bennink, Henriette Sjouwke, Tanneke Snijders-Groenendijk, Suzanne van de Vathorst, Leo van Vlimmeren, Anne Weenink, Willemien de Weerd, Ilse H. Zaal-Schuller

**Affiliations:** 1 Princess Máxima Center for Pediatric Oncology, Utrecht, the Netherlands; 2 Department of Pediatrics, Amalia Children’s Hospital, Radboud University Medical Center, Nijmegen, the Netherlands; 3 Department of Pediatrics, Erasmus Medical Center, Sophia Children’s Hospital, Rotterdam, the Netherlands; 4 Department of Pediatrics and Neonatology, Máxima Medical Center, Veldhoven, the Netherlands; 5 Department of Dermatology, Bravis Hospital, Roosendaal, the Netherlands; 6 Emma Palliative Care Team, Emma Children’s Hospital, Amsterdam University Medical Centre (UMC), Amsterdam, the Netherlands; 7 Department of Pediatrics, Beatrix Children’s Hospital, University Medical Centre Groningen, University of Groningen, Groningen, the Netherlands; 8 Wilhelmina Children’s Hospital, University Medical Centre Utrecht, Utrecht, the Netherlands; 9 Department of Pediatric Neurology, Radboud University Medical Center, Nijmegen, the Netherlands; 10 Dutch Knowledge Centre for Children’s Palliative Care, Utrecht, the Netherlands; 11 Stichting Kind en Ziekenhuis, Utrecht, the Netherlands; 12 the Netherlands Comprehensive Cancer Organization (IKNL), Utrecht, the Netherlands; 13 Department of Pediatrics, Emma Children’s Hospital, Amsterdam University Medical Centre (UMC), University of Amsterdam, Amsterdam, the Netherlands

**Keywords:** Clinical practice guideline, Evidence-based medicine, Paediatric palliative care, Symptom treatment

## Abstract

**Background:**

Children with life-threatening and life-limiting conditions can experience high levels of suffering due to multiple distressing symptoms that result in poor quality of life and increase risk of long-term distress in their family members. High quality symptom treatment is needed for all these children and their families, even more so at the end-of-life. In this paper, we provide evidence-based recommendations for symptom treatment in paediatric palliative patients to optimize care.

**Methods:**

A multidisciplinary panel of 56 experts in paediatric palliative care and nine (bereaved) parents was established to develop recommendations on symptom treatment in paediatric palliative care including anxiety and depression, delirium, dyspnoea, haematological symptoms, coughing, skin complaints, nausea and vomiting, neurological symptoms, pain, death rattle, fatigue, paediatric palliative sedation and forgoing hydration and nutrition. Recommendations were based on evidence from a systematic literature search, additional literature sources (such as guidelines), clinical expertise, and patient and family values. We used the GRADE methodology for appraisal of evidence. Parents were included in the guideline panel to ensure the representation of patient and family values.

**Results:**

We included a total of 18 studies that reported on the effects of specific (non) pharmacological interventions to treat symptoms in paediatric palliative care. A few of these interventions showed significant improvement in symptom relief. This evidence could only (partly) answer eight out of 27 clinical questions. We included 29 guidelines and two textbooks as additional literature to deal with lack of evidence. In total, we formulated 221 recommendations on symptom treatment in paediatric palliative care based on evidence, additional literature, clinical expertise, and patient and family values.

**Conclusion:**

Even though available evidence on symptom-related paediatric palliative care interventions has increased, there still is a paucity of evidence in paediatric palliative care. We urge for international multidisciplinary multi-institutional collaboration to perform high-quality research and contribute to the optimization of symptom relief in palliative care for all children worldwide.

**Supplementary Information:**

The online version contains supplementary material available at 10.1186/s12904-024-01367-w.

## Background

Worldwide, there are approximately 21 million children with conditions that can benefit from a palliative care approach [1]. Of these children, more than eight million need specialized paediatric palliative care [1, 2]. In the Netherlands, it is estimated that 7000 children, adolescents and young adults aged 0 to 20 years are living with life-threatening or life-limiting conditions and need palliative care [3]. Approximately, 23% of these children are diagnosed with oncological conditions and 77% have complex chronic conditions such as neonatal, neurological, or metabolic disorders [4, 5]. Annually, 1000 children die due to the consequences of these conditions [5]. All these children and their families require paediatric palliative care that focuses on improving quality of life and alleviating physical, psychological, social, and spiritual suffering [6].

As all children with life-threatening or life-limiting conditions can experience multiple distressing symptoms, high quality symptom treatment is an essential component of paediatric palliative care [2]. Previous international studies have reported high levels of suffering in children with cancer, complex chronic conditions, and advanced heart disease due to symptoms such as pain, dyspnoea, and fatigue [7–10]. Parents reported that these symptoms, which are often amenable to treatment, are insufficiently controlled [9, 10]. High levels of suffering due to symptoms decrease health-related quality of life in children and adolescents with life-threatening or life-limiting conditions [11, 12]. Poor quality of life affects not only the child but the whole family including parents and siblings [8, 13]. It also increases the risk of long-term distress in surviving family members [14]. Clearly, there is room for improvement in easing distress due to symptoms in these children. This is even more evident at the end of life, when suffering tends to worsen and attempts to control symptoms with traditional symptom-directed interventions are more likely to be unsuccessful [10, 15].

High quality symptom treatment should be ensured for all children with life-threatening or life-limiting conditions and their families. Clinical Practice Guidelines (CPGs) are powerful instruments that can facilitate consistent, efficient, and high-quality care by translating evidence into recommendations for clinical practice [16–18]. As a result, CPGs can contribute to the integration of high-quality palliative care into daily practice.

In 2013, the first Dutch CPG for Paediatric Palliative Care was published and provided the first recommendations on symptom treatment [19, 20]. Almost a decade after the development of the first Dutch CPG for paediatric palliative care, stakeholders expressed the need for an update and expansion of the CPG. Most importantly, health care providers and parents requested guidance on symptom treatment including treatment of refractory symptoms at the end-of life. Additionally, recommendations needed to be updated with evidence from new scientific literature. As a result, the first Dutch CPG for Paediatric Palliative care is revised and updated with recommendations on topics that were not covered in the first CPG [21].

The revised CPG provides recommendations on symptom treatment, advance care planning, shared decision-making, organisation of care, psychosocial care, and loss and bereavement care [21]. In this paper, we present the recommendations on symptom treatment and give an overview of the most recent evidence that was used to base recommendations upon. The recommendations on advance care planning, shared decision-making, and organisation of care; and psychosocial care and loss and bereavement care will be presented in two subsequent papers.

## Methods

The full methodology of the Dutch CPG for paediatric palliative care has been published in a separate paper [22].

### Scope

This guideline provides guidance on palliative care for all children aged 0 to 18 years with life- threatening or life-limiting conditions, their caregivers, and siblings (hereafter referred to as families) throughout the entire palliative trajectory (from palliative diagnosis till after end-of-life), with the ultimate goal to improve quality of palliative care and thereby quality of life of children and their families [6]. In this paper we provide recommendations for symptom treatment.

### Multidisciplinary guideline development panel

The guideline development panel consisted of an expert panel of 56 professionals with expertise in paediatric palliative care and a panel of nine (bereaved) parents (Appendix A). Professionals from multiple disciplines such as psychologists, neurologists, paediatricians, nurse practitioners, dermatologists, anaesthesiologists, intensivists, physical and occupational therapists, and specialists in paediatric rehabilitation and intellectual disabilities were included in the guideline panel. Professionals were selected based on their experience with paediatric palliative care, of whom some had specific certified training in this field. Within the guideline development panel, a core group of 11 experts was established to ensure consistency throughout the guideline. The other 45 experts were appointed to working groups (WGs) that focused on symptom treatment (WG 1) and refractory symptom treatment (WG 2). WGs covered multiple topics for which sub-WGs were established. WG 1 consisted of 11 sub-WGs that focused on non-pharmacological and pharmacological interventions of anxiety and depression, delirium, dyspnoea, haematological symptoms, coughing, skin complaints, nausea and vomiting, neurological symptoms, pain, death rattle, and fatigue. WG 2 focused on paediatric palliative sedation and forgoing hydration and nutrition. All topics were selected based on priorities of health care providers and parents [22]. An overview of the working structure and guideline development process is shown in Appendix B and C.

### Representation of patients and their families

Different methods were used to include perspectives of children and their families [22]. Two members of the core group were dedicated to ensure representation of child and family and their values during the entire guideline process. Additionally, a diverse panel consisting of nine (bereaved) parents of children with life-threatening or life-limiting conditions reviewed the first drafts of all guideline texts and recommendations and reviewed the complete concept guideline. We ensured that the panel represented a broad spectrum of experiences regarding paediatric palliative care by including parents of children with a variety of palliative conditions, age, and stage of disease (currently receiving palliative care or deceased).

### Formulation of clinical questions

The two WGs formulated a total of 27 clinical questions (Appendix D). WG1 formulated 24 clinical questions on the effect non-pharmacological and pharmacological interventions for symptom treatment. WG 2 formulated three clinical questions that focused on the effect of paediatric palliative sedation and the effect of forgoing hydration and nutrition.

### Search strategy and selection criteria

For the 27 clinical questions, we updated the literature search that was conducted for the former CPG (2013) [19] identifying randomized controlled trials (RCTs), controlled clinical trials (CCTs), or systematic reviews (SRs) of RCTs and CCTs on paediatric palliative care interventions (last update, January 24, 2020) (Appendix E). Studies were selected according to inclusion criteria related to study design (RCTs, CCTs, SRs of RCTs and CCTs), study population (children aged 0 to 18 with a life-threatening or life-limiting condition, according to the definition of the World Health Organization [6]) and study subject (paediatric palliative care interventions related to symptom treatment). Only studies published in English or Dutch language were included. Studies that described interventions on complementary or alternative medicine were excluded (Appendix F). We also searched for eligible studies in reference lists of included studies and identified SRs, guidelines, and textbooks. Moreover, we asked WG members to provide eligible studies.

### Summary and appraisal of evidence

To answer the clinical questions, we summarized included studies in evidence tables. We categorized evidence by outcome measures in summary of findings tables. Then, we formulated conclusions of evidence for each outcome measure. The quality of the total body of evidence was graded using the Grading of Recommendations Assessment, Development, and Evaluation (GRADE) method [22]. Study selection, summary and appraisal of evidence and formulation of conclusions was performed by one independent reviewer [22]. All processes were checked by members of the core group. In case the systematic literature search would yield little to no evidence, we searched for textbooks on paediatric palliative care and existing evidence-based guidelines on general paediatrics, and paediatric and adult palliative care in several guideline databases (Appendix E). Textbooks and guidelines were included if they were deemed relevant for the topics addressed in the (sub) WGs (Appendix F). We used textbooks and recommendations from guidelines to refine considerations and recommendations for this guideline.

### Translating evidence into recommendations

When translating the evidence into recommendations, several factors were taken into account: (1) the quality of the evidence (the higher the quality of evidence, the more likely it is to formulate a strong recommendation), (2) additional literature including textbooks and guidelines, (3) patient and family values and needs, (4) clinical expertise, (5) acceptability (legal and ethical considerations), (6) feasibility (sufficient time, knowledge, and manpower), and (7) benefits versus harms of the interventions. For each clinical question, WG members described the relevant considerations. Decisions regarding the final formulation of the recommendations were made through group consensus.

The strength of each recommendation was graded according to published evidence-based methods (appendix G) [23, 24]. Recommendations were categorised as strong to do (green), moderate to do (yellow) or strong not to do (red).

## Results

### Identification of evidence and additional literature

The systematic search for RCTs, CCTs and SRs of RCTs and CCTs on paediatric palliative care interventions yielded 5078 studies of which 168 were subjected to full-text screening. A total of 18 studies (three SRs of RCTs and 15 RCTs) on non-pharmacological and pharmacological interventions to treat symptoms were included (Appendix H). Furthermore, we included two textbooks, and 29 CPGs (six paediatric palliative care CPGs, 11 general paediatric CPGs and 12 adult palliative care CPGs) as additional literature to deal with lack of evidence (Appendix I).

#### Evidence

The evidence tables and summary of findings tables are presented in Appendix J and K and the conclusions of evidence are shown in Table 1.

**Table 1:**
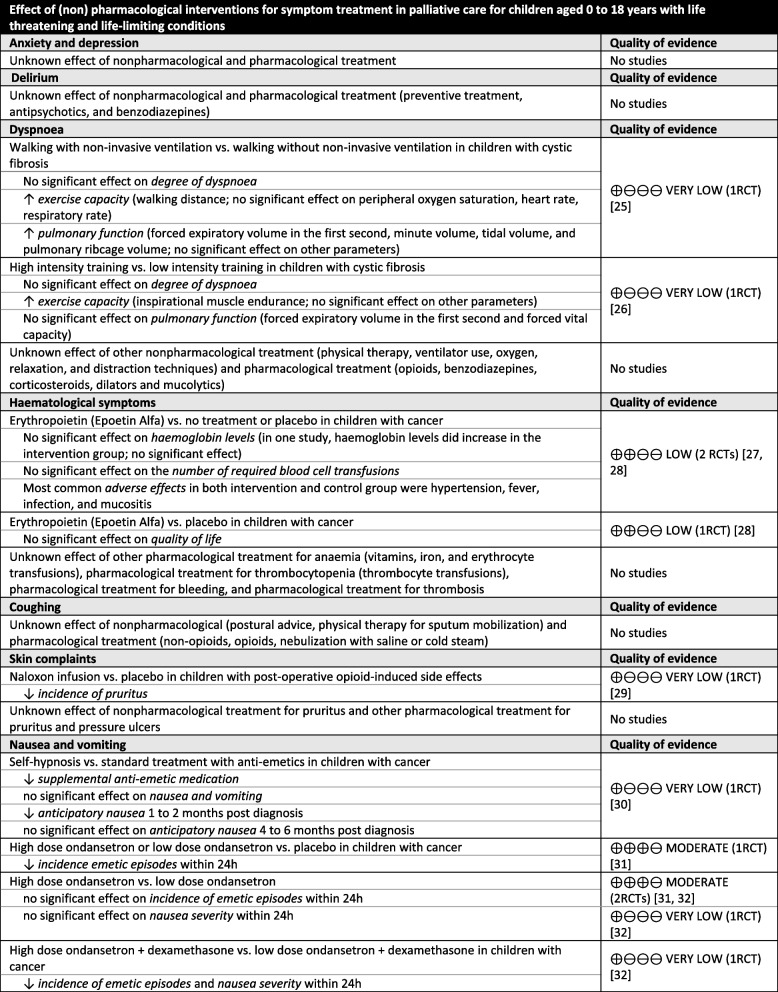

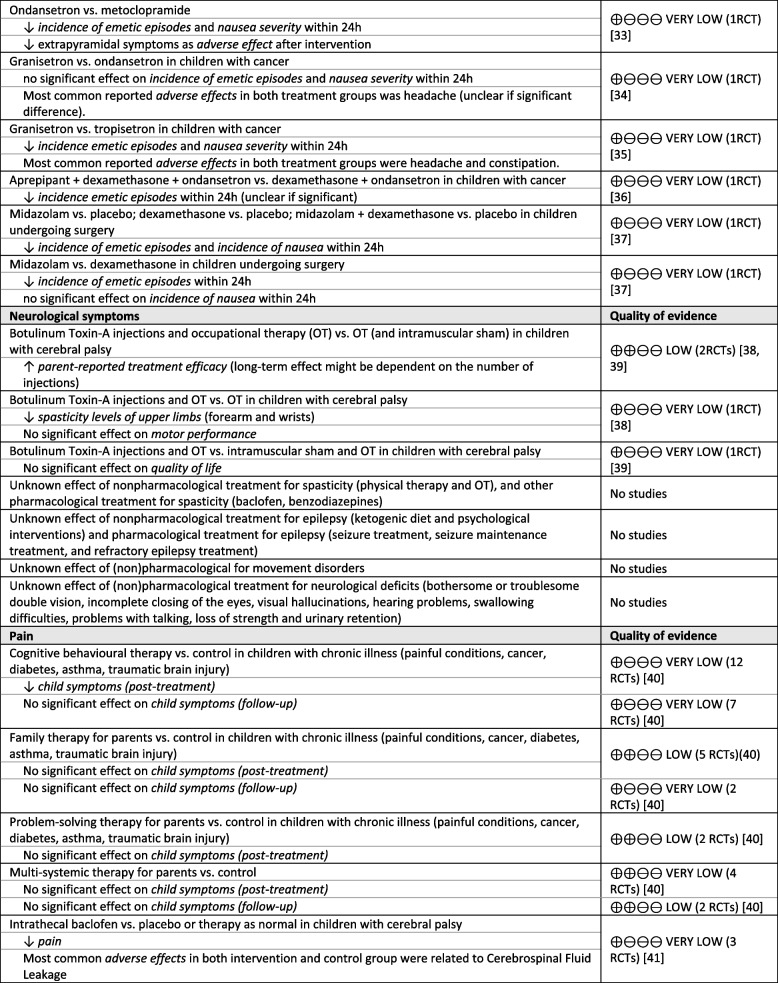
Conclusions of evidence on symptom treatment in paediatric palliative care [25–41]

^a^In the systematic review of Wiffen et al., no evidence on the effect of opioids on cancer-related pain was identified [42]

Studies reported on the effects of the following specific interventions: non-invasive ventilation [25] and high intensity training [26] for dyspnoea; erythropoietin for anaemia (haematological symptoms) [27, 28]; naloxone for pruritus (skin complaints) [29]; self-hypnosis [30] and anti-emetics including ondansetron [31–33], metoclopramide [33], granisetron [34, 35], tropisetron [35], aprepipant [36], midazolam [37] and dexamethasone [37] for nausea and vomiting; botulinum toxin-A injections and occupational therapy [38, 39] for spasticity (neurological symptoms); and psychological interventions for parents including cognitive behavioural therapy, family therapy, problem-solving therapy and multi-systemic therapy [40] and adjuvant medication including intrathecal baclofen, botulinum toxin A injections, oral alendronate, oral risedronate and intravenous pamidronate [41] for pain.

A few interventions showed significant improvement in relief of symptoms or quality of life among children with life-threatening or life-limiting conditions. Non-invasive ventilation and high intensity training significantly improved exercise capacity in children with cystic fibrosis (very low quality evidence) [25, 26]. One study showed that treatment with naloxone significantly decreased incidence of pruritus in children who received opioids postoperatively (very low quality evidence). Regarding nausea and vomiting, self-hypnosis significantly decreased supplemental anti-emetic medication use and anticipatory nausea in children with cancer (very low quality evidence) [30]. In addition, most anti-emetic medication including ondansetron, granisetron, aprepipant, midazolam and dexamethasone significantly decreased the incidence of emetic episodes and/or nausea severity (very low to moderate quality evidence) [31–37]. Concerning interventions for neurological symptoms, botulinum toxin-A injections significantly decreased spasticity levels of upper limbs and significantly increased parent-reported efficacy in children with cerebral palsy (very low to low quality evidence) [38, 39]. Furthermore, cognitive behavioural therapy for parents significantly decreased child symptoms including pain in children with chronic illnesses (very low quality evidence) [40]. Additionally, oral alendronate decreased pain in children with osteogenesis imperfecta (low quality evidence) [41].

For other interventions no significant effects were found. This included treatment with erythropoietin to improve anaemia in children with cancer (low quality evidence) [27, 28]. Regarding pain in children with chronic illnesses, no significant effect was found for family therapy, problem-solving therapy, and multi-systemic therapy for parents which aimed to improve child symptoms (very low to low quality evidence) [40]. Also, botulinum toxin A injections, oral risedronate, and intravenous pamidronate did not significantly decrease pain (very low to low quality evidence) [41]. Furthermore, the effect of opioids on cancer-related pain remains unknown, as the systematic review did not identify any studies on this topic [42].

#### Additional literature

Because there was limited evidence on paediatric palliative care interventions, we identified additional literature. The relevant recommendations from 29 guidelines on paediatric palliative care, general paediatrics and adult paediatric palliative care [43–71] and the textbooks [72, 73] on paediatric palliative care were used to refine considerations and recommendations.

### Translating evidence into recommendations

Recommendations were based upon the evidence, additional literature, clinical expertise, patient and family values, and other considerations such as costs and availability of medication. All members of the guideline development panel agreed that quality of life and values and needs of the child and family should be the main focus of every treatment-related decision. This was the starting point in the process of formulating recommendations.

Clinical experts, patient representatives and parents identified other key aspects that frequently influenced treatment-related recommendations. In addition to the treatment effects, the expected burden of treatment on the child was considered. Physical therapy techniques, for instance, can help to relief suffering due to coughing but are physically challenging and can only be considered if the child is willing and able to perform these techniques. Moreover, the adverse effects of potential interventions were considered. For example, starting antipsychotics to treat delirium carry a high risk of adverse effects [70]. Health care providers should be aware of these adverse effects of antipsychotics and monitor daily. If adverse effects occur, other medication should be considered. Finally, the child’s life-expectancy or prognosis is of importance. For example, vitamins and nutritional supplements should not be given to children with anaemia if life expectancy is short.

When formulating recommendations on paediatric palliative sedation and forgoing hydration and nutrition, clinical experts and parent representatives acknowledged the importance of thoughtful communication and careful preparation of all processes related to end-of-life care. Therefore, recommendations on refractory symptom treatment covered the entire process of paediatric palliative sedation and forgoing hydration and nutrition including communication, preparation, execution, and evaluation.

**Fig. 1:**
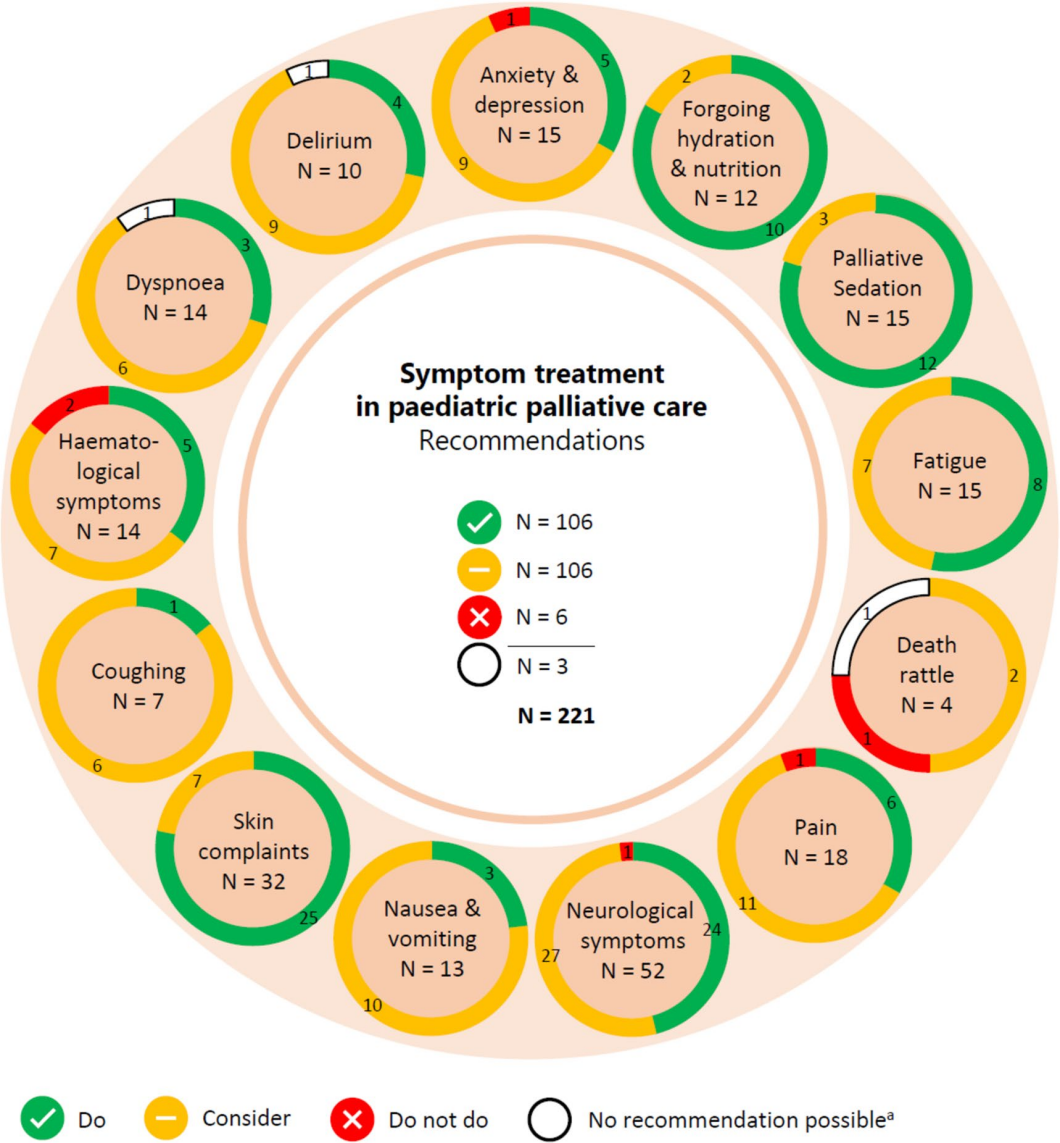
Number and strength of recommendations on symptom treatment in paediatric palliative care **a** It was not possible to formulate a recommendation due to insufficient evidence and lack of consensus among experts

We formulated a total of 221 recommendations on (non-)pharmacological treatment of anxiety and depression, delirium, dyspnoea, haematological symptoms, coughing, skin complaints, neurological symptoms, pain, death rattle, fatigue, paediatric palliative sedation and forgoing hydration and nutrition. Based on the level of evidence and other factors such as patient and family values, clinical expertise, and benefits and harms of the intervention, we formulated 106 strong recommendations to do (green), 106 weak recommendations to consider (yellow) and six strong recommendations not to do (red). In three situations, there was insufficient evidence and lack of consensus among experts to determine whether the benefits of the specific intervention outweigh potential harms. As a result, it was not possible to formulate a recommendation. In Fig. 1 we provide an overview of the number of recommendations on symptom treatment per topic. All recommendations are shown in Tables 2 and 3.

**Table 2:**
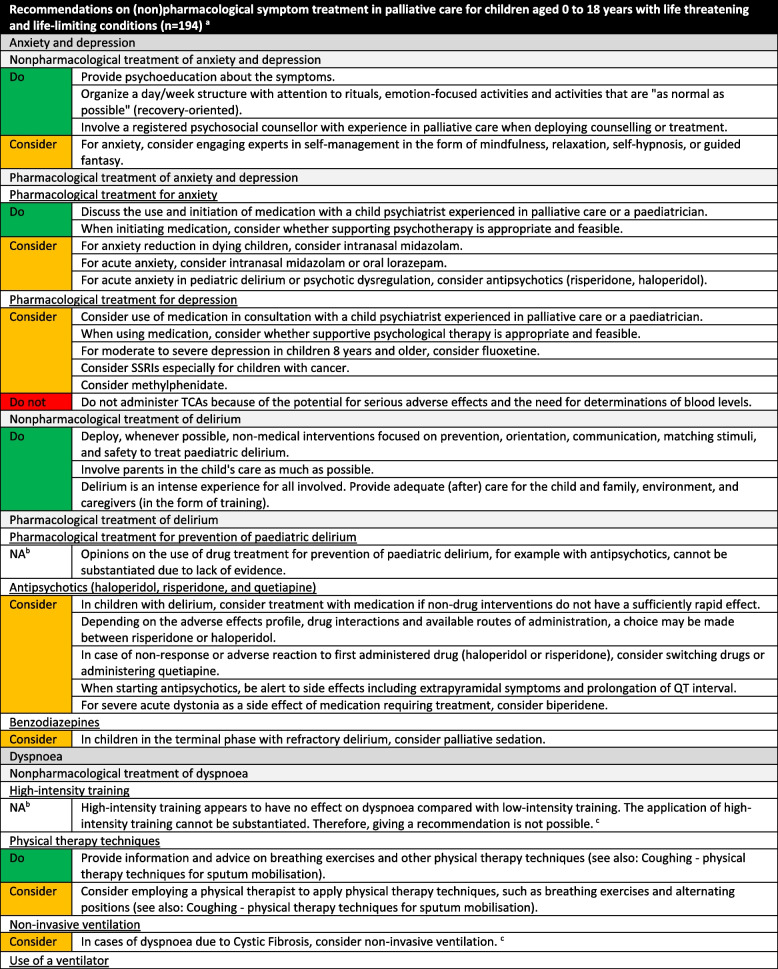

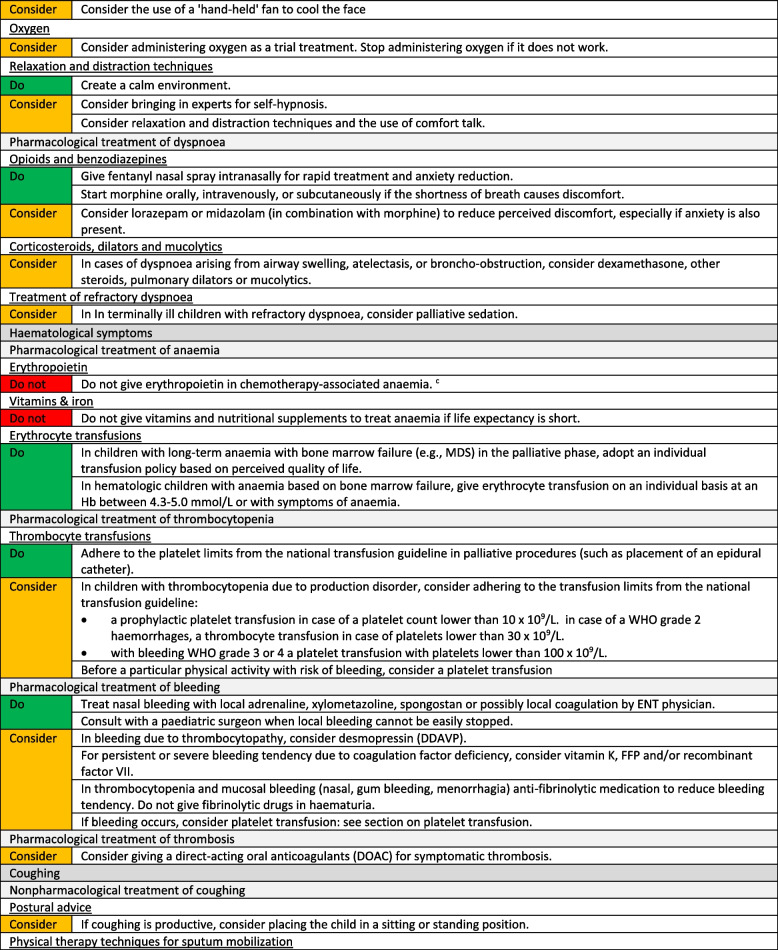

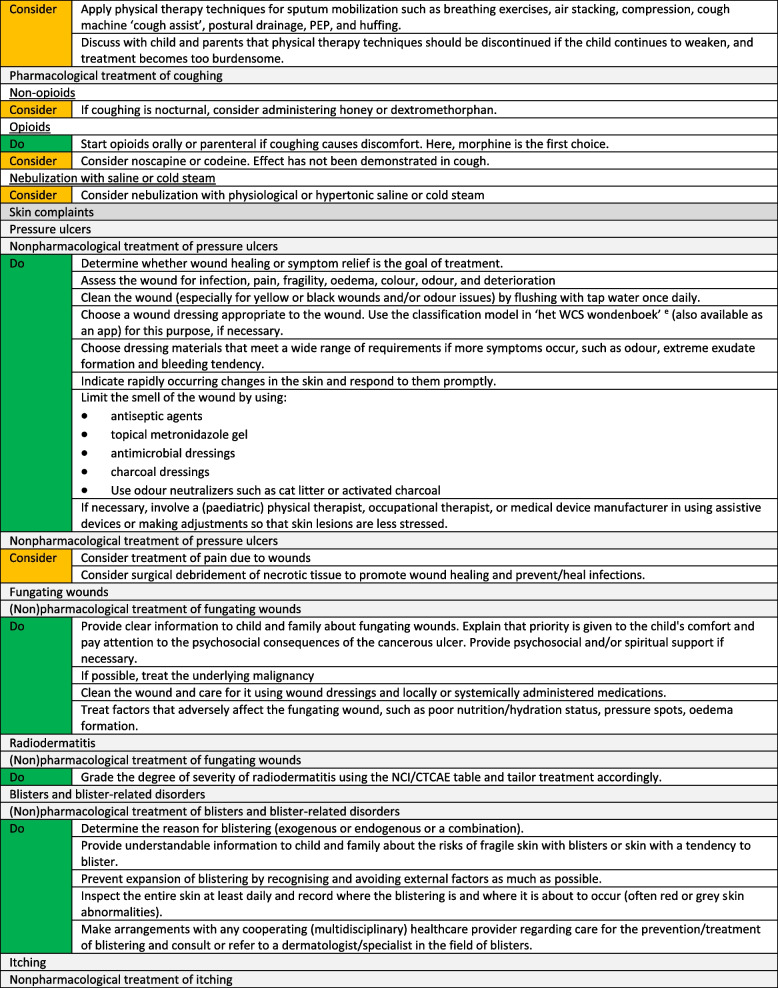

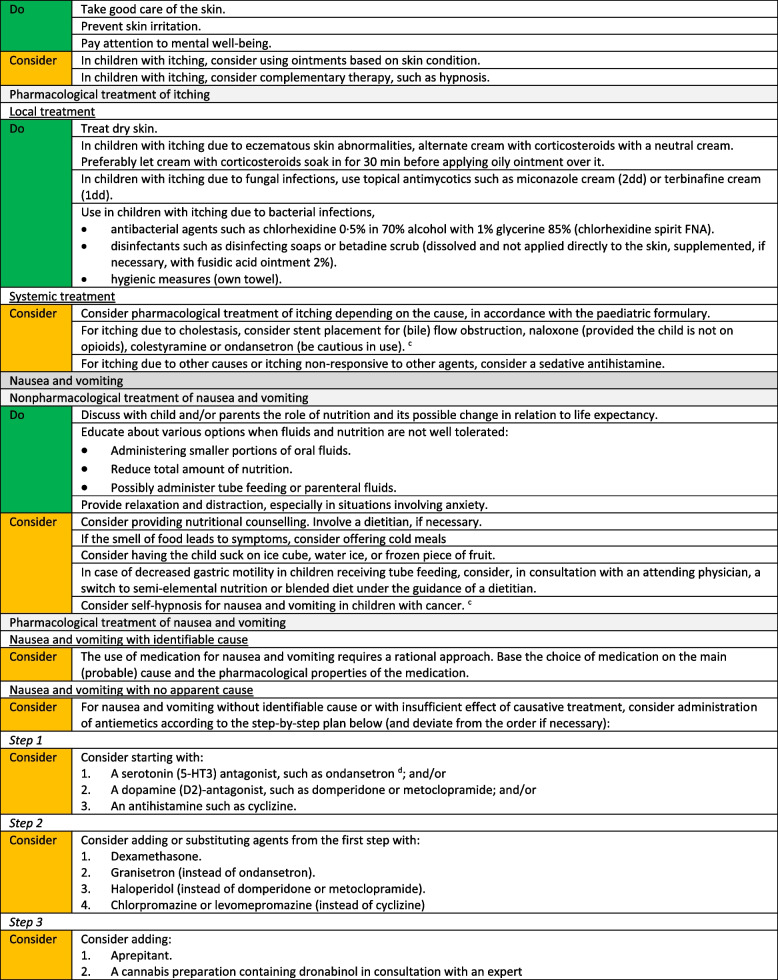

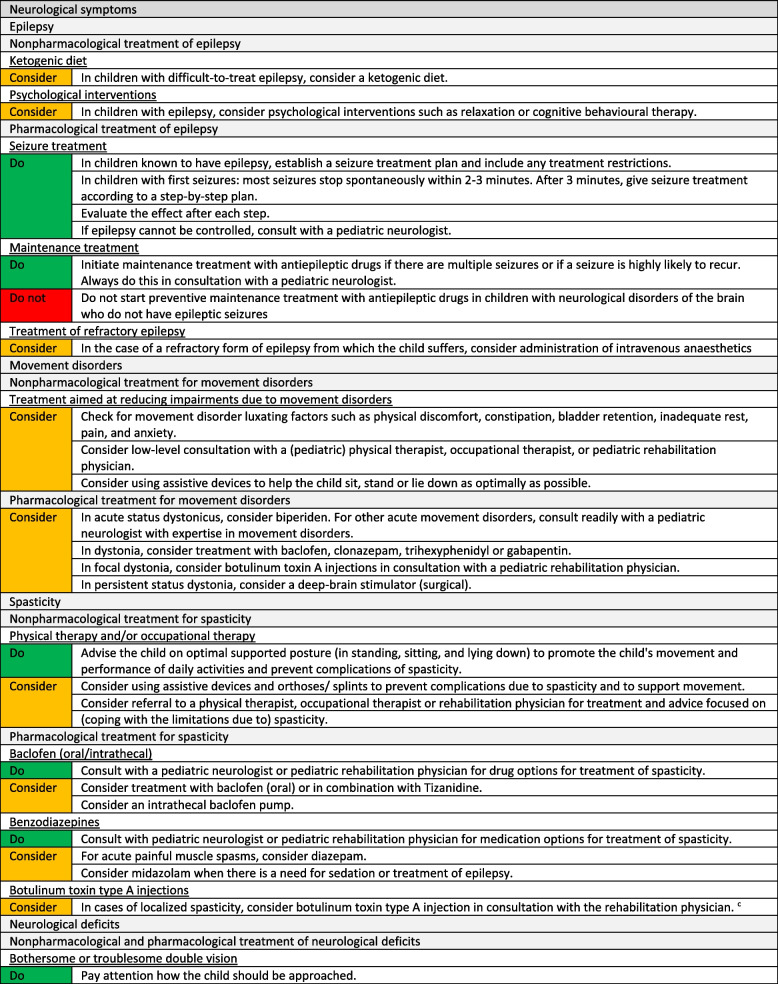

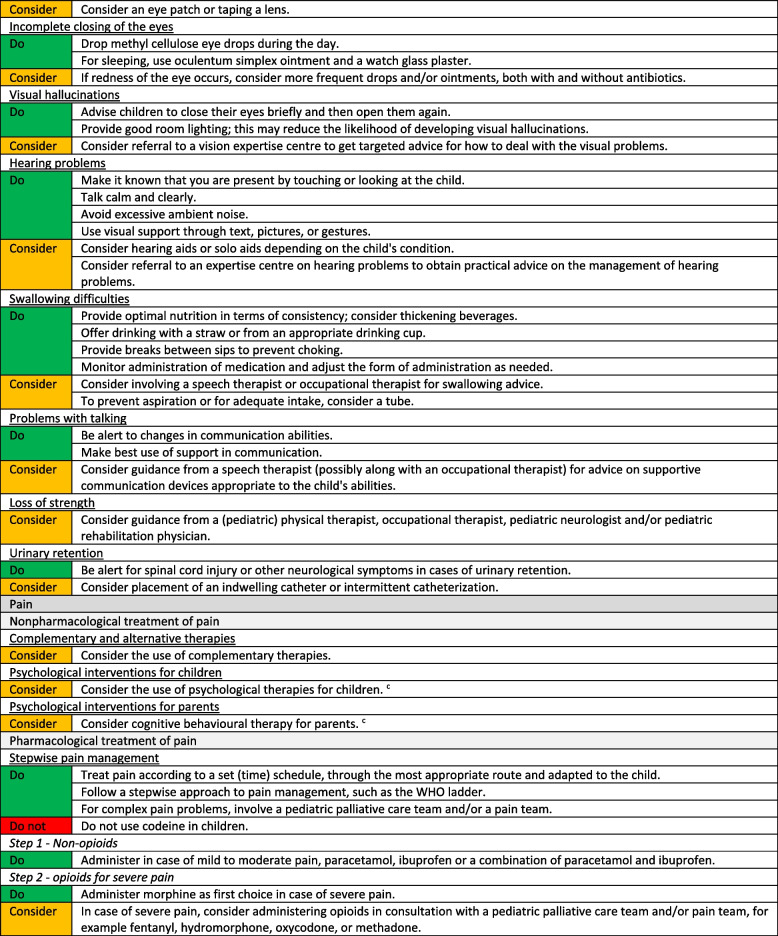
Recommendations on symptom treatment in paediatric palliative care

^a^This table shows the recommendations on (non) pharmacological treatment of symptoms. We also formulated recommendations on diagnosis and evaluation of symptoms for which we did not systematically search in scientific literature. These recommendations are available on request

^b^Not applicable, it was not possible to formulate a recommendation due to insufficient evidence and lack of consensus among experts

^c^For this recommendation, very low to low quality evidence was identified

^d^For this recommendation, moderate quality evidence was identified

^e^Only available in Dutch

**Table 3:**
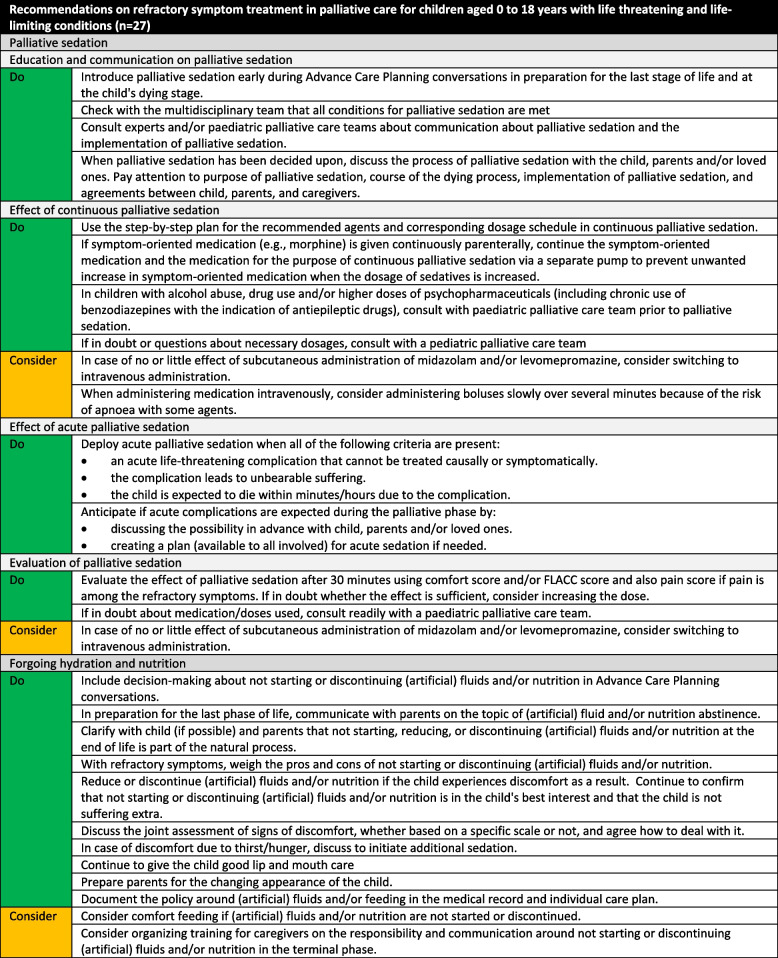
Recommendations on refractory symptom treatment in paediatric palliative care

## Discussion

Optimal treatment to relieve symptoms in children with life-threatening or life-limiting conditions is intense and challenging. Although progress has been made in improving and integrating paediatric palliative care in the Netherlands [4], health care providers, parents and other stakeholders have urged for more guidance to relieve physical suffering and ease distress in these children and their families. We responded to this need by developing recommendations on symptom treatment, including anxiety and depression, delirium, dyspnoea, haematological symptoms, coughing, skin complaints, nausea and vomiting, neurological symptoms, pain, death rattle, fatigue, paediatric palliative sedation and forgoing hydration and nutrition, as part of the revised Dutch CPG for paediatric palliative care. With these recommendations we aim to optimize symptom treatment in paediatric palliative care in the Netherlands. Furthermore, these recommendations can be used in other countries to optimize symptom treatment on a global scale.

This study has multiple strengths. First of all, the selection of symptoms was based upon priorities of clinical experts and parents [22]. This approach allowed us to provide recommendations on the symptoms that were most relevant to children with life-threatening or life-limiting conditions and their families. Furthermore, in this way, we were able to provide recommendations for a diverse group of children without limiting to a specific diagnosis. It should be noted that the selection of symptoms does not cover the full range of symptoms that may occur in paediatric palliative care. Still, our selection of symptoms is most comprehensive in comparison to other international guidelines on paediatric palliative care [66]. Additionally, we introduce the first evidence-based recommendations on paediatric palliative sedation in Europe.

Second, our recommendations on symptom treatment are based on an evidence-based methodology, meaning that we systematically searched for RCTs, CCTs and SRs of RCTs and CCTs in scientific literature. We identified 18 studies reporting on effectivity of several non-pharmacological and pharmacological interventions to treat symptoms. We found that since the development of the first Dutch guideline in 2013, the number of studies on paediatric palliative care interventions has increased [19, 22]. However, after allocating the studies to the relevant clinical questions, we concluded that the evidence could only (partly) answer eight out of 27 clinical questions. Also, the total body of evidence was rated as low to very low quality, mainly due to imprecision of effects (as a result of small number of participants) and potential risk of bias. For the other 19 clinical questions on effects of symptom treatment, we did not find evidence. Therefore, we developed a strategy to deal with this lack of evidence and included additional literature: 29 guidelines on paediatric palliative care, general paediatrics, and adult palliative care, and two textbooks on paediatric palliative care.

Finally, our recommendations are carefully developed according to a transparent and comprehensive guideline methodology [22]. We closely collaborated with experts in paediatric palliative care from multiple disciplines and parents. The transparency and the interactive relationship between all stakeholders increased validity and trustworthiness of our guideline process and recommendations.

The recommendations within this guideline are based on national clinical expertise, patient perspectives, and international evidence. We believe that these targeted recommendations on symptom treatment will be largely applicable to other contexts and can give guidance for symptom treatment in other countries as well. However, country-specific factors such as availability of non- pharmacological and pharmacological interventions, infrastructure, financial resources, and cultural backgrounds, should always be carefully considered before applying any recommendations in other contexts.

Unfortunately, we identified multiple gaps in knowledge for non-pharmacological and pharmacological interventions to treat symptoms (Table 4). Even though evidence on paediatric palliative care has increased there is still paucity in evidence on non-pharmacological and pharmacological interventions to treat symptoms [19, 74]. However, it should be noted that these knowledge gaps are based on our search that focused on paediatric palliative care only. Extrapolating evidence from general paediatrics might fill some knowledge gaps for treatment of symptoms. On the other hand, it is acknowledged that paediatric palliative care requires expertise that is often lacking in general paediatrics [75]. Extrapolating study results from general paediatrics is not always appropriate, so caution is needed when applying this evidence.

**Table 4:**
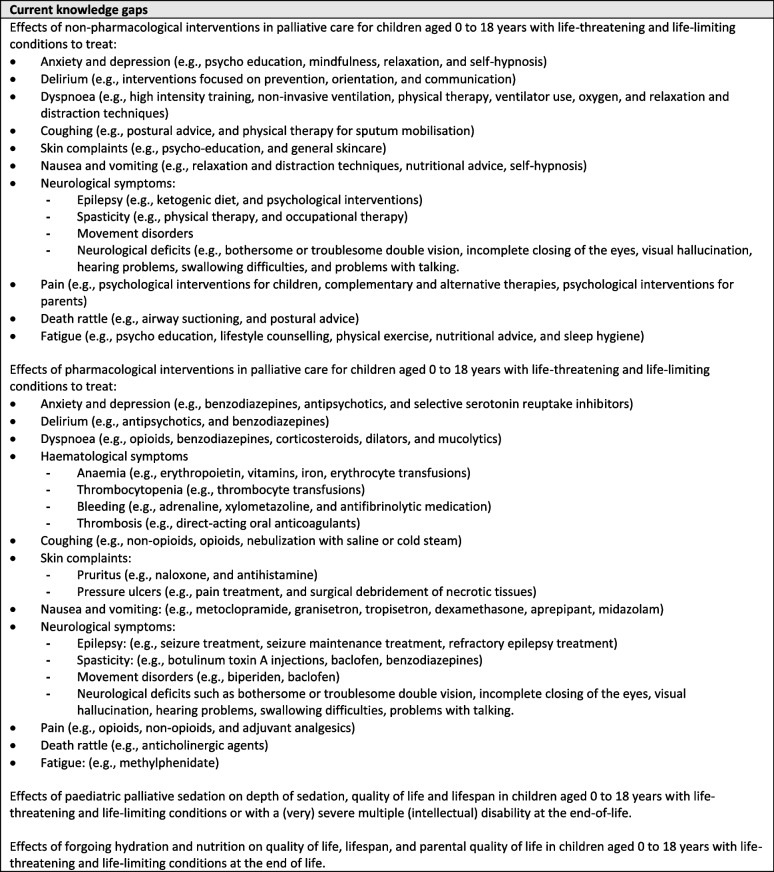
Knowledge gaps for symptom treatment in paediatric palliative care

It is clear that more research is required to relieve symptom-related suffering and to ease distress in children and family members. Future research should focus on international, multidisciplinary, and multi-institutional collaboration to reach higher numbers of participants, to broaden the scope of study questions and also improve study quality. In this way, we can strengthen the evidence base of our guideline and contribute to the optimization symptom treatment in paediatric palliative care. Additionally, attention should be given to facilitate implementation of knowledge and guidelines in paediatric palliative care for the purpose of achieving sufficient symptom relief in children with life-threatening and life-limiting conditions [75]. Furthermore, it should be noted that other factors such as access to financial resources and the organizational infrastructure of paediatric palliative care impact the quality of palliative care and differ among countries [2, 76]. These factors should be addressed to achieve optimal symptom treatment in paediatric palliative care on a global scale.

With these recommendations, we aim to limit symptom-related suffering and ease distress in children with life-threatening and life-limiting conditions and their families. Our methodology allowed us to provide evidence-based recommendations on a comprehensive selection of symptoms in close collaboration with experts in paediatric palliative care, and parents. Even though available evidence on symptom-related paediatric palliative care interventions has increased, there still is a paucity in evidence on non-pharmacological and pharmacological interventions to treat symptoms in paediatric palliative care. We urge for international multidisciplinary multi-institutional collaboration to perform high-quality research and to contribute to the optimization of symptom relief for all children with life-threatening or life-limiting conditions worldwide.

### Supplementary Information

**Additional file 1: Appendix A.** Paediatric palliative care guideline panel. **Appendix B.** Working structure for guideline development. **Appendix C.** Guideline development process. **Appendix D.** Clinical questions. **Appendix E.** Search strategies. **Appendix F.** Inclusion criteria. **Appendix G.** Criteria for grading levels of evidence and strength of recommendations. **Appendix H.** Flowchart of the study selection process. **Appendix I.** Results of the systematic literature search: included studies; **Appendix J.** Evidence tables; **Appendix K.** Summary of findings tables, appraisal of evidence, and conclusions of evidence.

## Data Availability

No datasets were generated or analysed during the current study.

## Abbreviations

CPG: Clinical Practice Guideline
CCT: Controlled Clinical Trial
GRADE: Grading Recommendation Assessment Development and Evaluation
RCT: Randomized Controlled Trial
SR: Systematic Review
WG: Working Group
WG1: Working group 1: Symptom treatment
WG2: Working group 2: Refractory symptom treatment
