# Supplementary material for: A Dutch paediatric palliative care guideline: a systematic review and evidence-based recommendations for symptom treatment
Source: BMC Palliat Care. 2024 Mar 13;23:72. doi: 10.1186/s12904-024-01367-w (PMC10935792; doi:10.1186/s12904-024-01367-w)
Supplement: Supplementary file 1 — Additional file 1: Appendix A. Paediatric palliative care guideline panel. Appendix B. Working structure for guideline development. Appendix C. Guideline development process. Appendix D. Clinical questions. Appendix E. Search strategies. Appendix F. Inclusion criteria. Appendix G. Criteria for grading levels of evidence and strength of recommendations. Appendix H. Flowchart of the study selection process. Appendix I. Results of the systematic literature search: included studies; Appendix J. Evidence tables; Appendix K. Summary of findings tables, appraisal of evidence, and conclusions of evidence. [file 12904_2024_1367_MOESM1_ESM.docx]

**Additional file**

A Dutch paediatric palliative care guideline: Evidence-based recommendations for symptom treatment

**Table of Contents**

[Appendix A. Paediatric palliative care guideline development panel 2](#_Toc152934836)

[Appendix B. Working structure for guideline development 4](#_Toc152934837)

[Appendix C. Guideline development process 6](#_Toc152934838)

[Appendix D. Clinical questions 7](#_Toc152934839)

[Appendix E. Search strategies 10](#_Toc152934840)

[Appendix F. Inclusion criteria 13](#_Toc152934841)

[Appendix G. Criteria for appraisal of evidence and strength of recommendations. 14](#_Toc152934842)

[Appendix H. Flowchart of the study selection process 15](#_Toc152934843)

[Appendix I. Results of the systematic literature search: included studies 16](#_Toc152934844)

[Appendix J. Evidence tables 19](#_Toc152934845)

[Appendix K. Summary of findings tables, appraisal of evidence, and conclusions of evidence 53](#_Toc152934852)

# Appendix A. Paediatric palliative care guideline development panel

**Expert panel**

Core group members

| Name | Role | Area of expertise |
| --- | --- | --- |
| Erna Michiels | Chair | Paediatric oncology, paediatric palliative care |
| Eduard Verhagen | Co-Chair | Paediatrics, paediatric palliative care |
| Kim van Teunenbroek | Coordinator | Guideline development (PhD candidate) |
| Leontien Kremer | Advisor | Guideline development, paediatrics, paediatric oncology |
| Renée Mulder | Advisor | Guideline development, paediatric oncology |
| Hester Rippen | Patient representative (Stichting Kind en Ziekenhuis) | |
| Johannes Verheijden | Patient representative (Dutch Knowledge Centre for Children’s Palliative Care) | |
| Brigitt Borggreve | Process support (advisor palliative care IKNL) | |
| Fleur Godrie | Process support (advisor palliative care IKNL) | |
| Inge van Trigt | Process support (advisor palliative care IKNL) | |
| Francis Essers | Process support (secretary IKNL) | |

Working group members

| Name | Profession | Working group - Role |
| --- | --- | --- |
| **WG chairs** | | |
| Jeffry Looijestein | Health care psychologist | Anxiety and Depression (WG 1A) – Chair |
| Jolanda Schieving | Paediatric neurologist | Delirium (WG 1B) - Chair  Neurological symptoms (WG 1H) - Chair |
| Carin Delsman-van Gelder | Paediatrician in training | Delirium (WG 1B) - Chair  Neurological symptoms (WG 1H) - Chair  Refractory symptom treatment (WG 2) – WG member |
| Marinka de Groot | Nurse practitioner specialized in paediatric palliative care | Dyspnoea (WG 1C) - Chair  Death rattle (WG 1J) – WG member  Refractory symptom treatment (WG 2) – WG member |
| Katja Heitink-Polle | Paediatric haematologist and oncologist | Haematological symptoms (WG 1D) - Chair |
| Inge Ahout | Paediatrician | Coughing (WG 1E) - Chair  Death rattle (WG 1J) - Chair  Nausea and vomiting (WG 1G) – WG member |
| Annemie Galimont | Dermatologist | Skin complaints (WG 1F) - Chair |
| Karin Bindels-de Heus | Paediatrician - genetic developmental disorders | Nausea and vomiting (WG 1G) - Chair |
| Maarten Mensink | Paediatric anaesthesiologist | Pain (WG 1I) - Chair |
| Selma Mulder | Child occupational therapist | Fatigue (WG 1K) - Chair  Neurological symptoms (WG 1H) - Reviewer |
| Netteke Schouten-van Meeteren | Paediatrician, paediatric oncologist | Refractory symptoms treatment (WG 2) - Chair |
| **WG members** | | |
| Esther van den Bergh | Clinical psychologist | Anxiety and Depression (WG 1A) – WG member  Fatigue (WG 1K) – WG member |
| Kim van der Schoot | Health care psychologist | Anxiety and Depression (WG 1A) – WG member |
| Hennie Knoester | Paediatrician, paediatric intensivist | Delirium (WG 1B) – WG member |
| Mariska Nieuweboer | Paediatric oncology nurse | Delirium (WG 1B) – WG member  Dyspnoea (WG 1C) – WG member  Haematological symptoms (WG 1D) – WG member  Pain (WG 1I) – WG member |
| Willemien de Weerd | Paediatrician | Dyspnoea (WG 1C) – WG member  Haematological symptoms (WG 1D) – WG member  Skin complaints - Reviewer |
| Saskia Gischler | Paediatrician, intensivist | Haematological symptoms (WG 1D) – WG member  Skin complaints (WG 1F) - Reviewer  Refractory symptom treatment (WG 2) – WG member |
| Arno Colenbrander | Paediatrician | Coughing (WG 1E) – WG member  Death rattle (WG 1J) – WG member |
| Govert Brinkhorst | Paediatrician, pulmonologist | Coughing (WG 1E) – WG member  Death rattle (WG 1J) – WG member |
| Leo van Vlimmeren | Child physical therapist | Coughing (WG 1E) – WG member  Death rattle (WG 1J) – WG member |
| Suzanne Pasmans | (Child)dermatologist, immunologist | Skin Complaints (WG 1F) – WG member |
| Barbara de Koning | Paediatrician, gastroenterologist | Nausea and vomiting (WG 1G) – WG member |
| Irma Rigter | Hospital pharmacist | Nausea and vomiting (WG 1G) – WG member |
| Christel Rohrich | Paediatric rehabilitation specialist | Neurological symptoms (WG 1H) – WG member  Fatigue (WG 1K) - Reviewer |
| Karin Geleijns | Neurologist | Neurological symptoms (WG 1H) – WG member |
| Ellen Siegers-Bennink | Pain consultant | Pain (WG 1I) – WG member |
| Tanneke Snijders-Groenendijk | Medical social care provider | Fatigue (WG 1K) – WG member |
| Mattijs Alsem | Paediatric rehabilitation specialist | Fatigue (WG 1K) – WG member |
| Liesbeth Ruijgrok | Hospital pharmacist | Refractory symptom treatment (WG 2) – WG member |
| Laurent Favié | Hospital pharmacist | Refractory symptom treatment (WG 2) – WG member |
| Cindy Joosen | Nurse practitioner specialized in paediatric palliative care | Refractory symptom treatment (WG 2) – WG member  Dyspnoea (WG 1C) – Reviewer  Death Rattle (WG 1J) - Reviewer |
| Linda Corel | Paediatrician, intensivist | Refractory symptom treatment (WG 2) – WG member  Dyspnoea (WG 1C) – Reviewer  Haematological symptoms (WG 1D) – Reviewer  Coughing (WG 1E) - Reviewer  Death Rattle (WG 1J) - Reviewer |
| Ilse Zaal-Schuller | Physician intellectual disabilities | Refractory symptom treatment (WG 2) – WG member |
| Hilda Mekelenkamp | Paediatric nurse | Refractory symptom treatment (WG 2) – WG member |
| Suzanne van de Vathorst | Professor medical ethics | Refractory symptom treatment (WG 2) – WG member |
| **WG reviewers** | | |
| Bas Oude Ophuis | Child and youth psychiatrist | Anxiety and Depression (WG 1A) – Reviewer |
| Lisette ‘t Hart-Kerkhoffs | Child and youth psychiatrist | Delirium (WG 1B) – Reviewer |
| Carla Juffermans | General practitioner, physician palliative care | Dyspnoea (WG 1C) – Reviewer  Haematological symptoms (WG 1D) – Reviewer  Coughing (WG 1E) - Reviewer |
| Emmy Räkers | General practitioner, physician intellectual disabilities | Neurological symptoms (WG 1H) – Reviewer |
| Henriette Sjouwke | Physician palliative care | Neurological symptoms (WG 1H) – Reviewer |
| Tom de Leeuw | Paediatric anaesthesiologist | Pain (WG 1I) - Reviewer |
| Jennifer van Dijk | Psychologist | Pain (WG 1I) - Reviewer |
| Anne Weenink | Medical pedagogical care provider | Pain (WG 1I) – Reviewer  Refractory symptom treatment (WG 2) – Reviewer |
| Elise van de Putte | Paediatrician - social paediatrics | Pain (WG 1I) – Reviewer  Fatigue (WG 1K) - Reviewer |
| Hanneke Heinen | Medical pedagogical care provider | Fatigue (WG 1K) - Reviewer |

**Patient representative panel**

| Name | Role |
| --- | --- |
| Marguerite Gorter-Stam | Parent |
| Suzan Mulder | Parent |
| Elisabeth Bruinja | Parent |
| Mark Mooij | Parent |
| José Bakker | Parent |
| Irma van Leeuwen | Parent |
| Jelke van Hoorn | Parent |
| Petra den Hollander | Parent |
| Marie-José Pulles | Parent |

# Appendix B. Working structure for guideline development


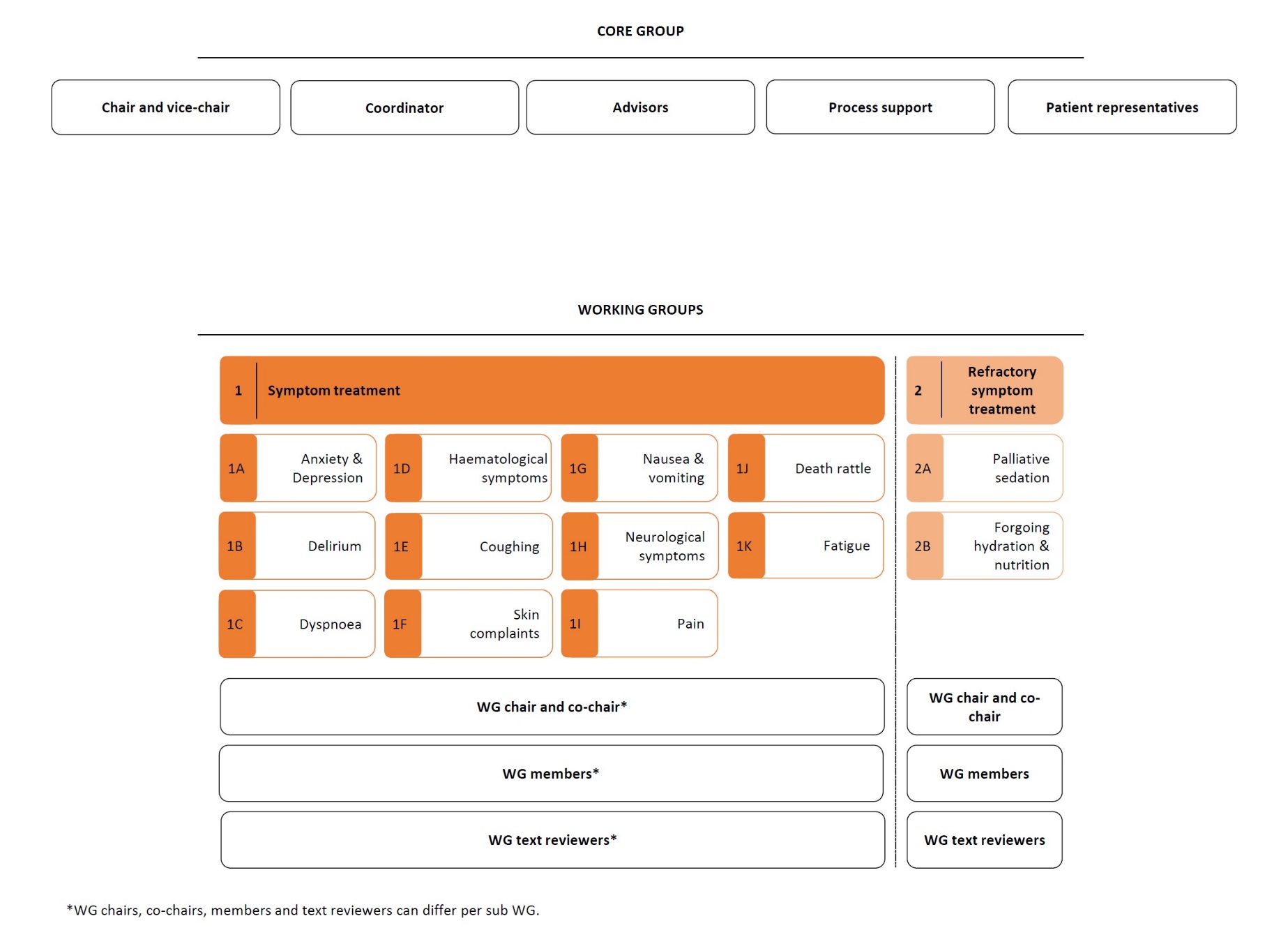


# Appendix C. Guideline development process


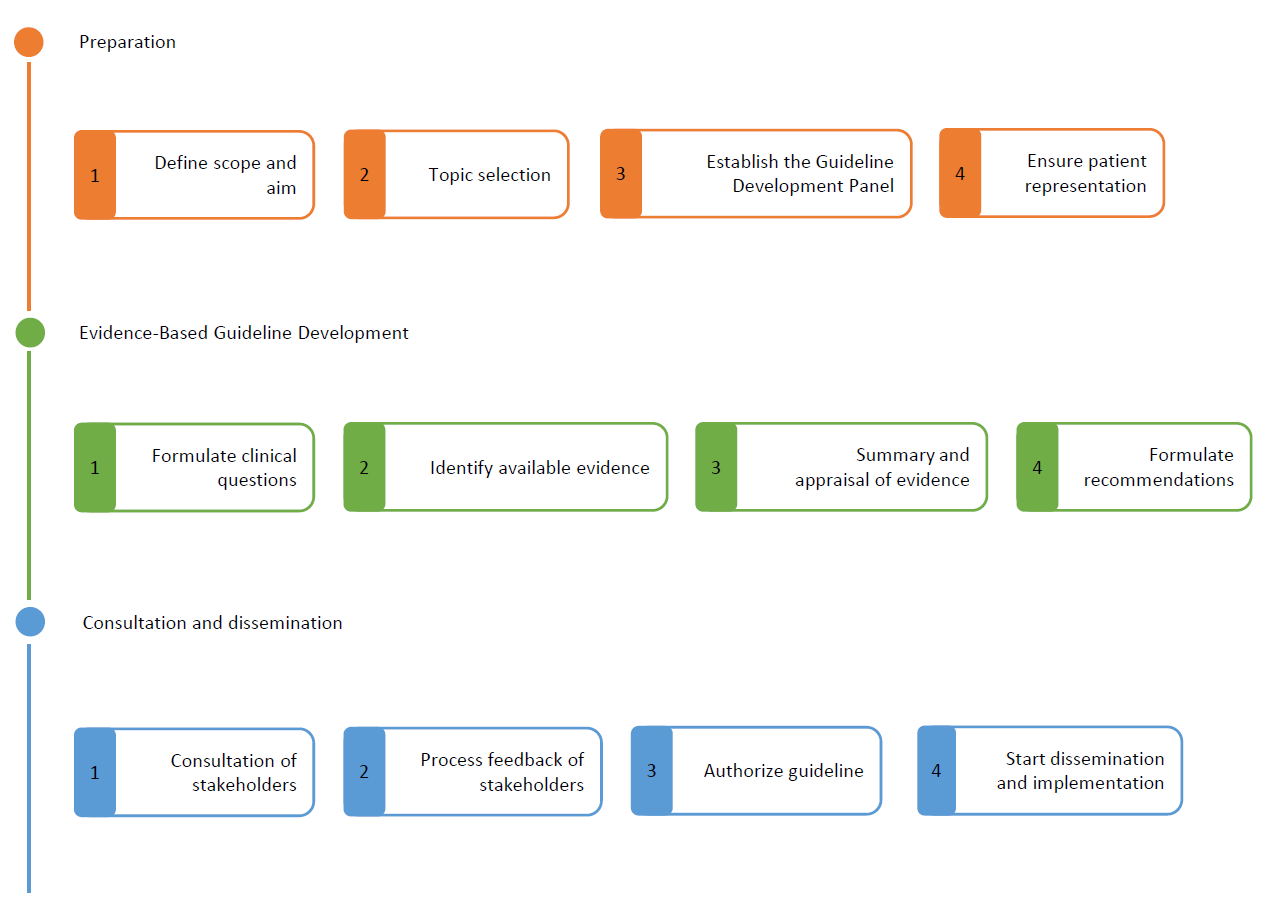


# Appendix D. Clinical questions

WG 1 Symptom treatment

*Sub-WG 1A Anxiety and Depression*

1. What is most effective non pharmacological intervention for anxiety and depression in children aged 0 to 18 years in the palliative phase?

1. What is most effective pharmacological intervention for anxiety and depression in children aged 0 to 18 years in the palliative phase?

*Sub-WG 1B Delirium*

1. What is most effective non pharmacological intervention for delirium in children aged 0 to 18 years in the palliative phase?
2. What is most effective pharmacological intervention for delirium in children aged 0 to 18 years in the palliative phase?

*Sub-WG 1C Dyspnoea*

1. What is most effective non pharmacological intervention for dyspnoea in children aged 0 to 18 years in the palliative phase?
2. What is most effective pharmacological intervention for dyspnoea in children aged 0 to 18 years in the palliative phase?

*Sub-WG 1D Haematological symptoms*

1. What the most effective pharmacological interventions for anaemia in children aged 0 to 18 in the palliative phase?
2. What is the most effective pharmacological intervention for thrombocytopenia in children aged 0 to 18 in the palliative phase?
3. What is the most effective pharmacological intervention for haemorrhages in children aged 0 to 18 in the palliative phase?
4. What is the most effective pharmacological intervention for thrombosis in children aged 0 to 18 in the palliative phase?

*Sub-WG 1E Coughing*

1. What is most effective non pharmacological intervention for coughing in children aged 0 to 18 years in the palliative phase?
2. What is most effective pharmacological intervention for coughing in children aged 0 to 18 years in the palliative phase?

*Sub-WG 1F Skin Complaints*

1. What is most effective non pharmacological intervention for skin complaints (itching, pressure ulcers and wounds) in children aged 0 to 18 years in the palliative phase?
2. What is most effective pharmacological intervention for skin complaints (itching, pressure ulcers and wounds) in children aged 0 to 18 years in the palliative phase?

*Sub-WG 1G Nausea and Vomiting*

1. What is most effective non pharmacological intervention for nausea and vomiting in children aged 0 to 18 years in the palliative phase?
2. What is most effective pharmacological intervention for nausea and vomiting in children aged 0 to 18 years in the palliative phase?

*Sub-WG 1H Neurological symptoms*

1. What is most effective non pharmacological intervention for neurological symptoms (epilepsy, spasticity, dyskinesia syndromes, loss of neurological function) in children aged 0 to 18 years in the palliative phase?
2. What is most effective pharmacological intervention for neurological symptoms (epilepsy, spasticity, dyskinesia syndromes, loss of neurological function) in children aged 0 to 18 years in the palliative phase?

*Sub-WG 1I Pain*

1. What is most effective non pharmacological intervention for pain in children aged 0 to 18 years in the palliative phase?
2. What is most effective pharmacological intervention for pain in children aged 0 to 18 years in the palliative phase?

*Sub-WG 1J Death rattle*

1. What is most effective non pharmacological intervention for death rattle in children aged 0 to 18 years in the palliative phase?
2. What is most effective pharmacological intervention for death rattle in children aged 0 to 18 years in the palliative phase?

*Sub-WG 1K Fatigue*

1. What is most effective non pharmacological intervention for fatigue in children aged 0 to 18 years in the palliative phase?
2. What is most effective pharmacological intervention for fatigue in children aged 0 to 18 years in the palliative phase?

WG2 Refractory symptoms

*Sub-WG 2A Palliative sedation*

1. What is the effect of palliative sedation with other medication than midazolam (possibly in combination with morphine) in children aged 0 to 18 years in the terminal phase on quality of life and lifespan?
2. What is the effect of palliative sedation with other medication than midazolam (possibly in combination with morphine) in children with multiple severe disabilities aged 0 to 18 years in the terminal phase on quality of life and lifespan?

*Sub-WG 2B Forgoing hydration & nutrition*

1. What is the effect of hydration and nutrition deprivation in children aged 0 to 18 years in the terminal phase on quality of life, life span and quality of life of parents?

# Appendix E. Search strategies

**Identification of evidence**

Search strategy

*Search A*

Search date October 5, 2018.

Databases OVID Medline, OVID PreMedline, Cochrane Library (CDSR & CENTRAL)

Search limits Publication date: 2010-present.

Language: English and Dutch.

Study design: Randomized Controlled Trials (RCTs), Clinical Controlled Trials (CCTs), and Systematic Reviews (of RCTs and/or CCTs).

*Search B*

Search date 24-01-2020

Databases PubMed (Medline)

Search limits Publication date: 2018 – present.

Language: English and Dutch.

Study design: Randomized Controlled Trials (RCTs), Clinical Controlled Trials (CCTs), and Systematic Reviews (of RCTs and/or CCTs).

Search strings

*Search A*

| Name researcher: | Joan Vlaayen |
| --- | --- |
| Search date: | 05-10-2018 |
| **Medline (OVID)** | |
| Search 1:  Intervention | 1. exp Palliative Care/ (49362) or 2. palliat*.tw. (58051) or 3. advanced disease*.tw. (15975) or 4. (end-stage disease* or end stage disease* or end-stage illness or end stage).tw. (52291) or 5. Terminally Ill/ (6207) or 6. Terminal Care/ (25841) or 7. (terminal* adj6 care*).tw. (3554) or 8. ((terminal* adj6 ill*) or terminal-stage* or dying or (close adj6 death)).tw. (37618) or 9. (terminal* adj6 disease*).tw. (3457) or 10. (end adj6 life).tw. (19644) or 11. hospice*.tw. (9908) |
| Search 2:  Population | 1. exp Infant/ (1075702) or 2. exp Child/ (1789770) or 3. Adolescent/ (1887787) or 4. (minors* or perinat* or postnat* or kid or kids or neonat* or newborn* or infan* or child* or adoles* or paediatric* or pediatric* or baby* or babies or toddler* or teen* or juvenil* or boy* or girl* or underag* or youth* or kindergar* or puber* or pubescen* or schools or nursery school* or preschool* or primary school* or secondary school* or elementary school* or high school* or highschool* or school age* or schoolage*).mp. (3978287) |
| Search 3:  Study design | 1. randomized controlled trial.pt. (468961) or 2. controlled clinical trial.pt. (92654) or 3. randomi?ed.ab. (442505) or 4. placebo.ab. (175141) or 5. clinical trials as topic.sh. (184906) or 6. randomly.ab. (255868) or 7. trial.ti. (163102) NOT 8. (exp animals/ not humans.sh. (4501441)) |
| Search 4:  Study design | 1. meta-analysis.mp,pt. or review.pt. or search:.tw. (2504080) |
| Combined: | Search 1 AND Search 2 AND (SEARCH 3 OR SEARCH 4) |
| Total records: | 2045 records |
| **PreMedline (OVID)** | |
| Search 1:  Intervention | 1. palliat*.tw. (7228) or 2. advanced disease*.tw. (1855) or 3. (end-stage disease* or end stage disease* or end-stage illness or end stage).tw. (6487) or 4. (terminal* adj6 care*).tw. (243) or 5. ((terminal* adj6 ill*) or terminal-stage* or dying or (close adj6 death)).tw. (3125) or 6. (terminal* adj6 disease*).tw. (237) or 7. (end adj6 life).tw. (2679) or 8. hospice*.tw. (944) |
| Search 2:  Population | 1. (minors* or perinat* or postnat* or kid or kids or neonat* or newborn* or infan* or child* or adoles* or paediatric* or pediatric* or baby* or babies or toddler* or teen* or juvenil* or boy* or girl* or underag* or youth* or kindergar* or puber* or pubescen* or schools or nursery school* or preschool* or primary school* or secondary school* or elementary school* or high school* or highschool* or school age* or schoolage*).mp. (195151) |
| Search 3:  Study design | 1. randomized controlled trial.pt. (277) or 2. controlled clinical trial.pt. (20) or 3. randomi?ed.ab. (51918) or 4. placebo.ab. (13994) or 5. randomly.ab. (36549) or 6. trial.ti. (20815) |
| Search 4:  Study design | 1. meta-analysis.mp,pt. or review.pt. or search:.tw. (170831) |
| Combined: | Search 1 AND Search 2 AND (SEARCH 3 OR SEARCH 4) |
| Total records: | 301 records |
| **Cochrane library (Cochrane Database of Systematic Reviews and CENTRAL)** | |
| Search 1:  Intervention | 1. MeSH descriptor: [Palliative Care] explode all trees (1454) OR 2. palliat*:ti,ab (4487) OR 3. advanced disease*:ti,ab (15208) OR 4. (end-stage disease* or end stage disease* or end-stage illness or end stage):ti,ab (8120) OR 5. MeSH descriptor: [Terminally Ill] explode all trees (80) OR 6. MeSH descriptor: [Terminal Care] explode all trees (407) OR 7. (terminal* NEAR/6 care*):ti,ab (110) OR 8. ((terminal* NEAR/6 ill*) or terminal-stage* or dying or (close NEAR/6 death)):ti,ab (1290) OR 9. (terminal* NEAR/6 disease*):ti,ab (143) OR 10. (end NEAR/6 life) (1966) OR 11. hospice*:ti,ab (529) |
| Search 2:  Population | 1. MeSH descriptor: [Infant] explode all trees (15033) OR 2. MeSH descriptor: [Child] explode all trees (1416) OR 3. MeSH descriptor: [Adolescent] explode all trees (97673) OR 4. (minors* or perinat* or postnat* or kid or kids or neonat* or newborn* or infan* or child* or adoles* or paediatric* or pediatric* or baby* or babies or toddler* or teen* or juvenil* or boy* or girl* or underag* or youth* or kindergar* or puber* or pubescen* or schools or nursery school* or preschool* or primary school* or secondary school* or elementary school* or high school* or highschool* or school age* or schoolage*):ti,ab (150225) |
| Combined: | Search 1 AND Search 2 |
| Total records: | Cochrane Database of Systematic reviews: 246 reviews; CENTRAL: 1496 |

*Search B*

| Name researcher | Kim van Teunenbroek |
| --- | --- |
| Search date: | 24-01-2020 |
| **PubMed (Medline)** | |
| Search 1:  Intervention | palliative care OR Care, Palliative OR Palliative Treatment OR Palliative Treatments OR Treatment, Palliative OR Therapy, Palliative OR Palliative Therapy OR Palliative Medicine OR Medicine, Palliative OR OR pediatric palliative care OR children's hospice OR Hospice care OR Terminal care OR “end life” |
| Search 2:  Population | infant OR infan* OR newborn OR newborn* OR new-born* OR baby OR baby* OR babies OR neonat* OR perinat* OR postnat* OR child OR child* OR schoolchild* OR schoolchild OR school child OR school child* OR kid OR kids OR toddler* OR adolescent OR adoles* OR teen* OR boy* OR girl* OR minors OR minors* OR underag* OR under ag* OR juvenil* OR youth* OR kindergar* OR puberty OR puber* OR pubescen* OR prepubescen* OR prepuberty* OR pediatrics OR pediatric* OR paediatric* OR peadiatric* OR schools OR nursery school* OR preschool* OR pre school* OR primary school* OR secondary school* OR elementary school* OR elementary school OR high school* OR highschool* OR school age OR schoolage OR school age* OR schoolage* OR infancy OR schools, nursery OR infant, newborn |
| Search 3: study design | (systematic review[tiab] OR review literature[mh] OR review[tiab] OR reviews[tiab] OR review[pt] OR systematic literature review[tiab] OR systematic literature review[tiab]) OR (meta analysis[pt] OR meta analysis[mh] OR meta analysis[tiab] OR metaanalysis[tiab] OR meta analyses[tiab]) OR (guidelin*[tiab]) |
| Search 4:  Study design | (randomized controlled trial [pt] OR controlled clinical trial [pt] OR randomized [tiab] OR placebo [tiab] OR drug therapy [sh] OR randomly [tiab] OR trial [tiab] OR groups [tiab]) NOT (animals [mh] NOT humans [mh]) |
| Combined: | Search 1 AND Search 2 AND (Search 3 OR Search 4) |
| Total records: | 990 |

**Identification of additional literature: guidelines**

Search strategy

Search date 24-01-2020

Databases Guideline International Network (GIN), IPOG, NICE, NVK en Pallialine.

Search limits Publication date: 2010-present;

Language: English and Dutch only.

Search strings

| Name researcher: | Kim van Teunenbroek |
| --- | --- |
| Search date: | 24-01-2020 |
| **Systematic search in GIN** | |
| Search 1:  Intervention | palliative care OR pediatric palliative care OR children's hospice |
| Search 2:  Population | child OR child* OR children* |
| Combined: | Search 1 AND Search 2 |
| Total records | 362 |
| **Focused search in databases of NICE, IPOG, NVK and Pallialine** | |
| Search NICE database | Guidelines on care for children, n = 1 |
| Search IPOG database | Guidelines on palliative care for children, n = 2 |
| Search NVK database | Guidelines on care for children, n = 2 |
| Search Pallialine database | Guidelines on palliative care for adults, n = 11 |
| Referencing | Guidelines on palliative care for children, n = 1  Guidelines on care for children, n = 6  Guidelines on palliative care for adults, n = 4 |
| Total records: | 27 |

# Appendix F. Inclusion criteria

**Identification of evidence**

| Patient  population | - Children with a life threatening or life limiting conditions according to the definition of the World Health Organisation (WHO)* aged 0 to 18 years old. This includes children with:   - acute life-threatening conditions from which recovery may or may not be possible,   - chronic life-threatening conditions that may be cured or controlled for a long period but also may cause death,   - progressive life-threatening conditions for which no curative treatment is available,   - severe neurologic conditions that are not progressive but may cause deterioration and death. - Patient population consisting of more than 10 patients. - 75% of the patient population consists of children aged 0 to 18 years old. |
| --- | --- |
| Intervention | All palliative interventions on:   - Treatment of anxiety and depression, delirium, dyspnoea, haematological symptoms, coughing, skin complaints, nausea and vomiting, neurological symptoms, pain, death rattle, and fatigue. - Treatment of refractory symptoms (palliative sedation and forgoing hydration & nutrition). |
| Study design | - Randomized Controlled Trials (RCTs) or Controlled Clinical Trials (CCTs). - Systematic reviews (of RCTs and/or CCTs). |
| Study characteristics | - English or Dutch language. - Minimum of two authors. - Abstract is available. |
| *World Health Organization. Integrating palliative care and symptom relief into paediatrics: a WHO guide for health-care planners, implementers, and managers. 2018 | |

**Identification of additional literature: guidelines**

| Patient population and study design | - Children with a life threatening or life limiting conditions according to the definition of the World Health Organisation (WHO)* aged 0 to 18 years old. This includes children with:   - acute life-threatening conditions from which recovery may or may not be possible,   - chronic life-threatening conditions that may be cured or controlled for a long period but also may cause death,   - progressive life-threatening conditions for which no curative treatment is available,   - severe neurologic conditions that are not progressive but may cause deterioration and death. - Guidelines on palliative care for adults with separate recommendations on palliative care for children. |
| --- | --- |
| Subjects | Guidelines on:   - Treatment of anxiety and depression, delirium, dyspnoea, haematological symptoms, coughing, skin complaints, nausea and vomiting, neurological symptoms, pain, death rattle, and fatigue. - Treatment of refractory symptoms (palliative sedation and forgoing hydration & nutrition). |
| Exceptions | - When paediatric palliative care guidelines on (refractory)symptom treatment were not available or inadequate, guidelines on symptom treatment in general paediatrics or adult palliative care** were included. |
| *World Health Organization. Integrating palliative care and symptom relief into paediatrics: a WHO guide for health-care planners, implementers, and managers. 2018  **Guidelines are only included if the recommendations were considered relevant for children in the palliative phase | |

# Appendix G. Criteria for appraisal of evidence and strength of recommendations.

| **Grade of Recommendation**  **Conclusions of evidence according to GRADE** | **Strong recommendation**  **to do**  Benefits >>> risk & harms | **Moderate**  **recommendation**  **to do**  Benefits > or = risk & harms | **Recommendation**  **not to do**  No benefit / Potentially harm |
| --- | --- | --- | --- |
| **High quality of evidence**  Consistent evidence from well performed and high-quality studies or systematic reviews (low risk of bias, direct, consistent, precise). | Strong recommendation based on high quality evidence | Moderate recommendation based on high quality evidence | Recommendation not to do based on high quality evidence |
| **Moderate quality of evidence**  Evidence from studies or systematic reviews with few important limitations. | Strong recommendation based on moderate quality evidence | Moderate recommendation based on moderate quality evidence | Recommendation not to do based on moderate quality evidence |
| **Low to very low quality of evidence**  Evidence from studies with serious flaws, only expert opinion, or standards of care. | Strong recommendation based on expert opinion | Moderate recommendation based on (very) low quality evidence  Diverging expert opinions | Recommendation not to do based on expert opinion |
|  | **Wording in recommendations:** | |  |
|  | We strongly recommend … | We moderately recommend … | We do not recommend … |

Gibbons RJ, Smith S, Antman E. American College of Cardiology/American Heart Association clinical practice guidelines: Part I: where do they come from? Circulation. 2003; 107(23): 2979-86.

# Appendix H. Flowchart of the study selection process


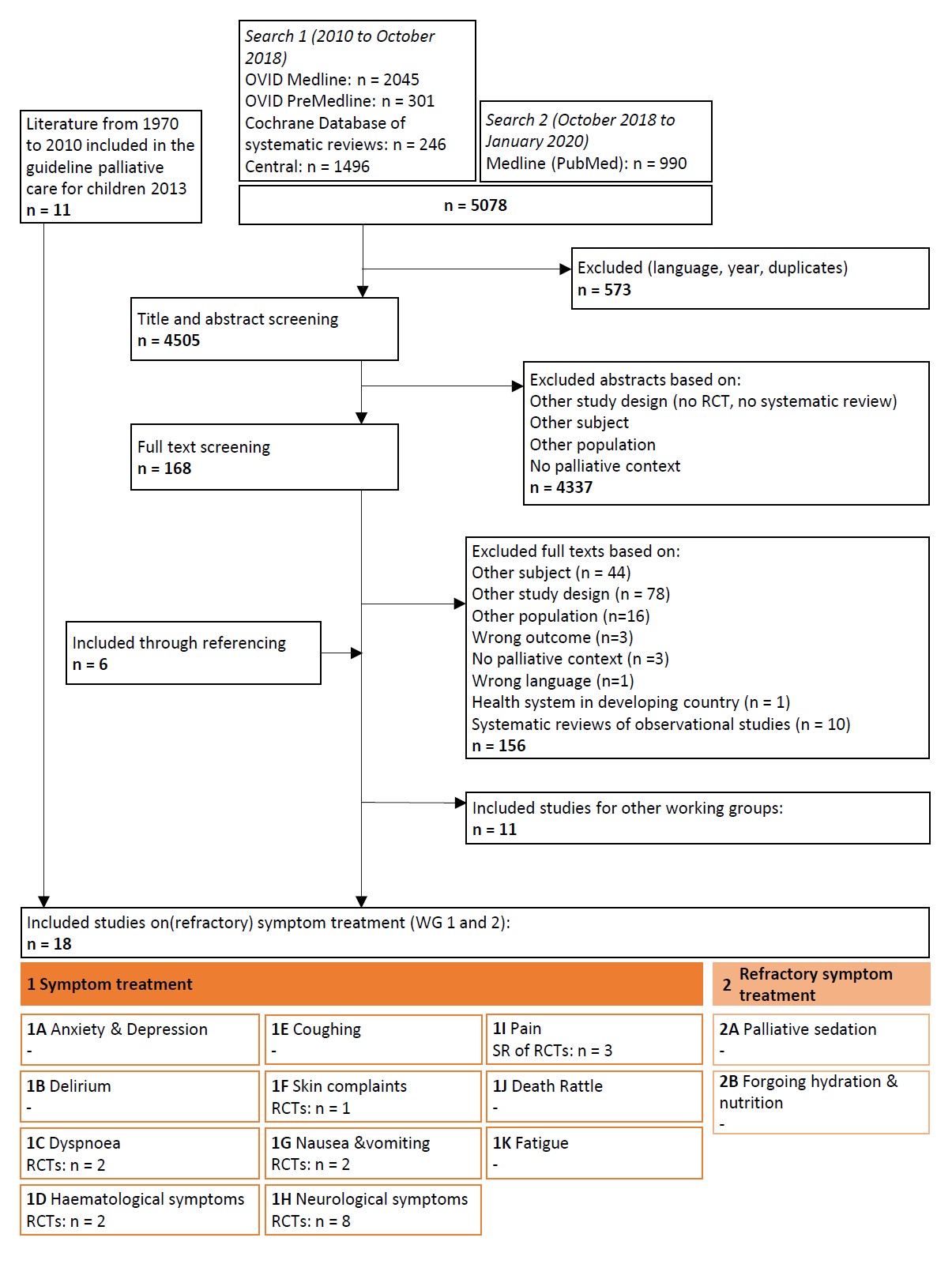


# Appendix I. Results of the systematic literature search: included studies

**Identification of evidence**

WG1 Symptom treatment

*Sub-WG 1C Dyspnoea*

| **Year** | **Bibliography** | **Study design** |
| --- | --- | --- |
| 2014 | ***Lima C et al.*** Effects of noninvasive ventilation on treadmill 6-min walk distance and regional chest wall volumes in cystic fibrosis: randomized controlled trial. Respir Med. 2014;108(10):1460-8. | RCT |
| 2001 | ***De jong W et al.*** Inspiratory muscle training in patients with cystic fibrosis. Respir Med. 2001;95(1):31-6. | RCT |

*Sub-WG 1D Haematological symptoms*

| **Year** | **Bibliography** | **Study design** |
| --- | --- | --- |
| 2002 | ***Buyukpamukcu M et al.*** Is epoetin alfa a treatment option for chemotherapy-related anemia in children? Medical and Pediatric Oncology. 2002;39(4):455-8. | RCT |
| 2006 | ***Razouk BI et al.*** Double-blind, placebo-controlled study of quality of life, hematologic end points, and safety of weekly epoetin alfa in children with cancer receiving myelosuppressive chemotherapy. J Clin Oncol. 2006;24(22):3583-9. | RCT |

*Sub-WG 1F Skin Complaints*

| **Year** | **Bibliography** | **Study design** |
| --- | --- | --- |
| 2005 | ***Maxwell LG et al.*** The Effects of a Small-Dose Naloxone Infusion on Opioid-Induced Side Effects and Analgesia in Children and Adolescents Treated with Intravenous Patient-Controlled Analgesia: A Double-Blind, Prospective, Randomized, Controlled Study. Anesthesia & Analgesia. 2005;100(4):953-8. | RCT |

*Sub-WG 1G Nausea and Vomiting*

| **Year** | **Bibliography** | **Study design** |
| --- | --- | --- |
| 1994 | ***Jacknow DS et al.*** Hypnosis in the prevention of chemotherapy-related nausea and vomiting in children: a prospective study. J Dev Behav Pediatr. 1994;15(4):258-64. | RCT |
| 1996 | ***Brock P et al.*** An increased loading dose of ondansetron: a north European, double-blind randomised study in children, comparing 5 mg/m2 with 10 mg/m2. Eur J Cancer. 1996;32(10):1744-8. | RCT |
| 1999 | ***Parker RI et al***. Randomized, double-blind, crossover, placebo-controlled trial of intravenous ondansetron for the prevention of intrathecal chemotherapy-induced vomiting in children. J Pediatr Hematol Oncol. 2001;23(9):578-81. | RCT |
| 1994 | ***Orchard PJ et al.*** A prospective randomized trial of the anti-emetic efficacy of ondansetron and granisetron during bone marrow transplantation. Biol Blood Marrow Transplant. 1999;5(6):386-93 | RCT |
| 1998 | ***Kóseoglu V et al.*** Comparison of the efficacy and side-effects of ondansetron and metoclopramide-diphenhydramine administered to control nausea and vomiting in children treated with antineoplastic chemotherapy: a prospective randomized study. Eur J Pediatr. 1998;157(10):806-10 | RCT |
| 2001 | ***Aksoylar S et al.*** Comparison of tropisetron and granisetron in the control of nausea and vomiting in children receiving combined cancer chemotherapy. Pediatr Hematol Oncol. 2001;18(6):397-406. | RCT |
| 2009 | ***Gore L et al.*** Aprepitant in adolescent patients for prevention of chemotherapy-induced nausea and vomiting: a randomized, double-blind, placebo-controlled study of efficacy and tolerability. Pediatr Blood Cancer. 2009;52(2):242-7. | RCT |
| 2007 | ***Riad, W. et al.*** Effect of midazolam, dexamethasone and their combination on the prevention of nausea and vomiting following strabismus repair in children. Eur J Anaesthesiol. 2007;24(8):697-701. | RCT |

*Sub-WG 1H Neurological symptoms*

| **Year** | **Bibliography** | **Study design** |
| --- | --- | --- |
| 2010 | ***Olesch CA et al.*** Repeat botulinum toxin-A injections in the upper limb of children with hemiplegia: a randomized controlled trial. Dev Med Child Neurol. 2010;52(1):79-86. | RCT |
| 2014 | ***Copeland I et al.*** Botulinum toxin A for nonambulatory children with cerebral palsy: a double blind randomized controlled trial. J Pediatr. 2014;165(1):140-6 e4. | RCT |

*Sub-WG 1J Pain*

| **Year** | **Bibliography** | **Study design** |
| --- | --- | --- |
| 2015 | ***Eccleston C et al.*** Psychological interventions for parents of children and adolescents with chronic illness. Cochrane Database Syst Rev. 2015(4):CD009660. | Systematic review of RCTs |
| 2015 | ***Beecham E et al.*** Pharmacological interventions for pain in children and adolescents with life‐limiting conditions. Cochrane Database of Systematic Reviews. 2015(3):CD010750. | Systematic review of RCTs |
| 2011 | ***Wiffen PJ et al.*** Opioids for cancer‐related pain in children and adolescents. Cochrane Database of Systematic Reviews 2017 7): The Journal of Clinical Endocrinology and Metabolism 2011;96(2):355–64. | Systematic review of RCTs |

**Additional literature**

Textbooks

| **Year** | **Bibliography** | **Study design** |
| --- | --- | --- |
| 2012 | ***Goldman A et al.*** Oxford Textbook of Palliative Care for Children. 2nd ed. Oxford: Oxford University Press; 2012. | Textbook paediatric palliative care |
| 2011 | ***Wolfe J et al.*** Textbook of Interdisciplinary Pediatric Palliative Care: Saunders; 2011. | Textbook paediatric palliative care |

Guidelines

| **Year** | **Bibliography** | **Study design** |
| --- | --- | --- |
| 2022 | ***Nederlandse vereniging voor kindergeneeskunde.*** Erytrocytentransfusies bij kinderen & neonaten met kanker 2022 (updated 2022, June 29). | Guideline paediatric palliative care |
| 2022 | ***Nederlandse Vereniging voor Kindergeneeskunde.*** Trombocytentransfusies bij kinderen met kanker 2022 (updated 2022, June 29). | Guideline paediatric palliative care |
| 2019 | ***Anderson A-K et al.*** Artificial nutrition and hydration for children and young people towards end of life: consensus guidelines across four specialist paediatric palliative care centres. BMJ Support Palliat Care. 2019; 11:92-100. | Guideline paediatric palliative care |
| 2016 | ***National Institute for Health and Care Excellence.*** End of life care for infants, children and young people with life-limiting conditions: planning and management. London: NICE; 2016 (updated 2019, July 25). | Guideline paediatric palliative care |
| 2016 | ***Flank J et al.*** Guideline for the Treatment of Breakthrough and the Prevention of Refractory Chemotherapy-Induced Nausea and Vomiting in Children With Cancer. Pediatr Blood Cancer. 2016;63(7):1144-51 | Guideline paediatric palliative care |
| 2014 | ***Dupuis LL et al.*** Guideline for the prevention and treatment of anticipatory nausea and vomiting due to chemotherapy in pediatric cancer patients. Pediatr Blood Cancer. 2014;61(8):1506-12. | Guideline paediatric palliative care |
| 2021 | ***Nederlandse Vereniging voor Psychiatrie.*** Multidisciplinaire richtlijn pediatrisch delier (PD) en emergence delier (ED) 2021 (updated 2021, November 11). | Guideline general paediatrics |
| 2020 | ***Nederlandse Vereniging voor Neurologie.*** Epilepsie. 2020. | Guideline general paediatrics |
| 2019 | ***National Institute for Health Care and Excellence.*** Depression in Children and Young People: identification and mangement. London: NICE; 2019. | Guideline general paediatrics |
| 2019 | ***National Institute for Health and Care Excellence.*** Epilepsies: diagnosis and management London: NICE; 2012 (updated 2021, May 12). | Guideline general paediatrics |
| 2019 | ***Federatie Medisch Specialisten.*** Bloedtransfusiebeleid. 2019 (updated 2020, October 15). | Guideline general paediatrics |
| 2019 | ***Nederlandse Vereniging voor Kindergeneeskunde.*** Nederlandse Vereniging voor Kindergeneeskunde. Somatisch onvoldoende verklaarde lichamelijke klachten (SOLK) bij kinderen. 2019 (updated 2019, March 3) | Guideline general paediatrics |
| 2016 | ***Bolier L et al****.* JGZ-Richtlijn Angst. Utrecht: Trimbos-Instituut; 2016 (updated 2016) | Guideline general paediatrics |
| 2016 | ***Oud M et al.*** JGZ-richtlijn Depressie. Utrecht: Trimbos-instituut; 2016 (updated 2016) | Guideline general paediatrics |
| 2012 | ***National Institute for Health and Care Excellence.*** Spasticity in under 19s: Management. London: NICE; 2012 (updated 2016, November 29) | Guideline general paediatrics |
| 2012 | ***Nederlands Centrum Jeugdgezondheid.*** Huidafwijkingen, taakomschrijving en richtlijn voor de preventie, signalering, diagnostiek, begeleiding, behandeling en verwijzing. 2012 (updated 2012, May). | Guideline general paediatrics |
| 2010 | ***Joanna Briggs Institute.*** Effectiveness of non-pharmacological pain management in relieving chronic pain for children and adolescents. Best Practice: evidence-based information sheets for health professionals. 2010;14(17):1-4. | Guideline general paediatrics |
| 2022 | ***Integraal Kankercentrum Nederland.*** Richtlijn Jeuk in de palliatieve fase. 2022 (updated 2022, February 21). | Guideline adult palliative care |
| 2022 | ***Integraal Kankercentrum Nederland.*** Richtlijn palliatieve sedatie. 2022 (updated 2022, June 16). | Guideline adult palliative care |
| 2021 | ***Verpleegkundigen & Verzorgenden Nederland.*** Richtlijn Decubitus. 2021. | Guideline adult palliative care |
| 2019 | ***Integraal Kankercentrum Nederland.*** Vermoeidheid bij kanker in de palliatieve fase (3.0). 2019 (updated 2019, May 9). | Guideline adult palliative care |
| 2018 | ***Verpleegkundigen & Verzorgenden Nederland.*** Smetten (Intertrigo) preventie en behandeling. 2018 (updated 2018, September). | Guideline adult palliative care |
| 2016 | ***Nederlandse Vereniging van Revalidatieartsen.*** Cerebrale en/of spinale spasticiteit: VRA; 2016 (updated 2016, January 1) | Guideline adult palliative care |
| 2015 | ***Integraal Kankercentrum Nederland.*** Dyspneu in de palliatieve fase (3.0). 2015 (updated 2015, December 22). | Guideline adult palliative care |
| 2015 | ***National Institute for Health and Care Excellence.*** Care of dying adults in the last days of life. London: NICE; 2015 (updated 2015, December 16) | Guideline adult palliative care |
| 2014 | ***Integraal Kankercentrum Nederland.*** Misselijkheid en braken (4.0). 2014 (updated 2014, June 16). | Guideline adult palliative care |
| 2010 | ***Integraal Kankercentrum Nederland.*** Oncologische Ulcera 2010 (updated 2010, August 11). | Guideline adult palliative care |
| 2010 | ***Integraal Kankercentrum Nederland.*** Hoesten (2.0). 2010 (updated 2010, June 18). | Guideline adult palliative care |
| 2010 | ***Integraal Kankercentrum Nederland.*** Zorg in de stervensfase (1.0). 2010. | Guideline adult palliative care |

# Appendix J. Evidence tables

## *Sub-WG 1C Dyspnoea*

| **Non pharmacological treatment of dyspnoea** | | | | |
| --- | --- | --- | --- | --- |
| ***Lima C et al.*** Effects of non-invasive ventilation on treadmill 6-min walk distance and regional chest wall volumes in cystic fibrosis: Randomized controlled trial. Respir Med 2014;108:1460–1468 | | | | |
| **Study characteristics** | **Patient characteristics** | **Intervention / Control** | **Outcomes / Results** | **Comments**  **Risk of bias** |
| Type of study:  open randomized controlled crossover clinical  trial  Setting:  1Centre, Brazil  Duration:  No follow-up. All outcomes are measured 30 minutes before TWT and directly after TWT.  Study years:  Not reported  Protocol published in register: (*clinicaltrials.gov / WHO register*)  Not reported | Number and type of participants:  *An open randomized controlled cross-over trial was conducted. Participants acted as their own control*  13 children and adolescents with Cystic Fibrosis (clinically stable, with no history of hospitalization for respiratory failure in last 3 months.   - Intervention group: 13   Start: Assessment of CF with NIV, N = 6  Start: Assessment of CF without NIV, N = 7   - Control group: 13   Start: Assessment of CF with NIV, N = 6  Start: Assessment of CF without NIV, N = 7  Age:   - Intervention group: Mean: 10.7, Range 7-15 yrs. - Control group: Mean: 10.7, Range 7-15 yrs.   Sex:   - Intervention group:   Start: Assessment of CF with NIV  M: n = 3; F: n = 3  Start: Assessment of CF without NIV  M: n =5, F: n = 2   - Control group:   Start: Assessment of CF with NIV  M: n = 3; F: n = 3  Start: Assessment of CF without NIV  M: n =5, F: n = 2 | *Open randomized controlled cross-over trial*  Procedure:  1.Baseline Optoelectronic plethysmography (OEP) which assesses variations in compartmental chest wall volume and ventilator pattern – duration = 3 min  2. Baseline Spirometry which assesses pulmonary function – duration = 5 min  3. Resting time – 30 min  4. Treadmill Walking Test (TWT) with or without Non-invasive Ventilation (depending on randomization)  5. OEP after, duration = 3 min  6. Spirometry after, duration = 5 min  24/48 rest  Same procedure, only the treadmill walking test will be without or with ventilation (depends on start procedure)  Type of intervention:  Non-invasive ventilation (NIV) in walk distance (WD) in the treadmill walking test (TWT)  Before the test patients are submitted to NIV on a BiLevel mode for 30 minutes.  Treadmill test initiated with a speed of 2.5km/h every 30s the patient was asked if the speed could be increased, maintained or decreased. Speed could not exceed 7 km/h.  Type of control:  No use of Noninvasive ventilation (NIV) in walk distance (WD) in the treadmill walking test (TWT) | Outcome definitions:  **Primary outcome**   - Walk distance (WD) in the treadmill walking test (TWT), meter   **secondary outcomes**  *Cardiorespiratory variables*   - peripheral O2 saturation (SpO2) - heart rate (HR) - respiratory rate (RR), - Score on the Borg dyspnoea scale (BDS), score ranging from 0 (none) to 10 (maximum), higher score indicating higher level of dyspnoea.   *pulmonary function variables*   - forced expiratory volume in the first second (FEV1), - forced vital capacity (FVC) - forced expiratory flow of 25%e75% of FVC (FEF 25e75)   *Variables resulting from OEP analysis*   - minute volume (MV) - tidal volume (Vt), - pulmonary rib cage volume (Vrcp), - abdominal rib cage volume (Vrca) - abdominal volume (Vab), - inspiratory time (Ti), - expiratory time (Te), - total ventilatory cycle time (Ttot) - duty cycle (Ti/Ttot) - frequency/tidal volume ratio (RR/Vt)   Results (per outcome)  **Walking distance (intervention vs control)**  Mean (SD) 415.38m (77.52) vs 386.92m (84.89), P = 0.039.  *Cardiorespiratory variables*  There was no significant difference between intervention and control group immediately after the TWT for all cardio respiratory outcomes (SpO2, HR, RR and BS)  *Pulmonary function variables*  Intervention vs Control   - no significant difference in FEV1 (%)/FVC (ml/%)/ FEF25-75 (ml/% between groups   Before vs After TWT  Intervention group:   - Significant increase in FEV1 (ml) after TWT, p = 0.036 - no significant difference FEV1 (%)/FVC (ml/%)/ FEF25-75 (ml/%) before and after TWT   Control group:   - no significant difference in pulmonary function variables FEV1 (ml/%)/FVC (ml/%)/ FEF25-75 (ml/%) before and after TWT   Variables resulting from OEP analysis (MV, Vt, Vrcp, vrca, vab, ti, te, Ttot, Ti/Ttot, RR/VT)  Intervention vs control   - no significant difference in MV, Vt, Vrcp, vrca, vab, ti, te, Ttot, Ti/Ttot, RR/VT between groups   Before and after TWT  Intervention group:   - Significant increase in MV after TWT, p =0.013 - Significant increase in Vt after TWT, p = 0.005 - Significant increase in Vrcp after TWT, p =, 0,011 - no significant difference in vrca, vab, ti, te, Ttot, Ti/Ttot, RR/VT before and after TWT   Control group:   - no significant difference in MV, Vt, Vrcp, vrca, vab, ti, te, Ttot, Ti/Ttot, RR/VT | Strengths:  Well performed study  Limitations:  No conflict of interest  Only for CF during activities  **Risk of bias**  A. Selection bias:  low risk  Reason:  A randomized plan was compiled using the website randomization.com, applying a generator of random-permuted blocks to define the order in wich patients would execute the treadmill walking test (with or without NIV). Allocation concealment very likely because of use computer  B. Attrition bias:  Low risk  Reason:  No loss to follow-up    C. Performance bias  High risk  Reason: Researchers and patient were not blinded (not possible)  D. Detection bias  Unclear  Reason:  Blinding of outcome assesors was not reported  **Main conclusions**  The pulmonary impairment in cystic fibrosis patients can  increase the ventilatory demand even in performing them  Activities of daily living. |

No evidence table is available for the study of ‘De jong W et al. Inspiratory muscle training in patients with cystic fibrosis. RESPIRATORY MEDICINE (2001) 95, 31–362’.

## *Sub-WG 1D Haematological symptoms*

| **Pharmacological treatment for Anaemia** | | | | |
| --- | --- | --- | --- | --- |
| ***Buyukpamukcu M et al.*** Is Epoetin Alfa a treatment option for chemotherapy-related anaemia in children? Med Pediatr Oncol 2002;29 (4):455-8 | | | | |
| **Study characteristics** | **Patient characteristics** | **Intervention / Control** | **Outcomes / Results** | **Comments**  **Risk of bias** |
| Type of study:  RCT  Setting:  1 centre, Turkey  Duration:  Intervention duration was 2 months  Study years:  Not reported  Protocol published in register:  Not reported | Number and type of participants:  Total of 34 children with cancer- or chemotherapy related anaemia.   - *Intervention group:* N = 17 - *Control group:* N = 17   Age:  Median: 5yr, Range 1-16 yr.  Sex:  M: 20 (58,8%), F: 14 (41,2%)  Other:  Number and percentage of Cancer Treatments included in the study.   - Platinum-based chemotherapy: N = 15, 44,1% - Nonplatinum-based chemotherapy: N = 19, 55,9% - Local regional radiotherapy: N = 13, 38,2% - Cranial and/or spinal radiotherapy: N = 7, 20,6% | All patients  Intervention duration was 8 weeks  Physical examinations, blood counts and blood pressure measurement were performed weekly.  Transfusions were administered if Hb levels dropped below 6g/dL.  No Iron supplementation or granulocyte colony-stimulating factor was given to the patients during the study period  Type of intervention:  Serum erythropoietin was measured at the beginning and end of the study.  Epoetin Alfa was administered at a dose of 150IU/kg, 3 times a week subcutaneously for 8 weeks  Type of control:  Serum erythropoietin (EPO) was measured at the beginning of the study | Outcome definitions:  **Hematologic parameters:** Neutrophil counts, thrombocyte counts, serum EPO levels  **Haemoglobin level:** Mean Hb levels g/dL  **Red Blood Cell (RBC) transfusion requirements:** Number of transfusions required  **Safety:** Occurrence of complications (hypertension)  Results (per outcome)  **Hematologic parameters:**   - *Neutrophil counts:* no significant difference in comparison to control group - *Thrombocyte counts*; no significant difference in comparison to control group - *Serum EPO levels:* No significant difference between the Epoetin Alfa and control groups regarding serum EPO levels   **Haemoglobin levels (g/dL):**  *Mean haemoglobin level at study entry (intervention vs. control):*  8.5 g/dL vs 8.48 g/dL, P = NS  *Mean haemoglobin level at Study end (intervention vs. control):*  10.21 g/dL vs 8.41 g/dL, p = 0.027  *Mean Hb over the course of the study*   - *Intervention group:* Significant increase in the Epoetin Alfa group from 8.50 to 10.21 g/dL, p = 0.086 - *Control group:* No significant increase in the Epoetin Alfa group from 8.48 to 8.41 g/dL   **RBC transfusion requirements**  *Number of transfusions required (intervention vs. control):*  1 (5.9%) vs 8 (47.0%), p = 0.08  **Safety**  1 patient in the intervention developed hypertension after 2 weeks of Epoetin Alfa treatment. Epoetin alfa was continued after 1 week without further complications. | Strengths:  -  Limitations:  Small group size  **Risk of bias**  A. Selection bias:  Unclear  Reason: patients were randomly assigned to either the Epoetin Alfa group or control group. Allocation concealment was not reported in the study  B. Attrition bias:  Unclear  Reason: Loss of follow-up/dropout was not reported in the study  C. Performance bias  Unclear  Reason: Blinding of patients and personnel was not reported in the study  D. Detection bias  Unclear  Reason: Blinding of outcome assessors was not reported in the study |

| **Pharmacological treatment for Anaemia: Epoetin Alfa** | | | | |
| --- | --- | --- | --- | --- |
| ***Razouk BI et al.*** Double-Blind, Placebo-Controlled Study of Quality of Life, Hematologic End Points, and Safety of Weekly Epoetin Alfa in Children With Cancer Receiving Myelosuppressive Chemotherapy. J Clin Oncol 2006; 24:3583-3589. | | | | |
| **Study characteristics** | **Patient characteristics** | **Intervention / Control** | **Outcomes / Results** | **Comments**  **Risk of bias** |
| Type of study:  Double-Blind Placebo-Controlled RCT  Setting:  27 sites, USA  Duration:  Study visits occurred ever 3 / 4 weeks.  Final follow-up 4 months after the beginning of intervention.  Study years:  2000-2003  Protocol published in register:  Protocol published in ClinicaltTrials.gov | Number and type of participants:  Total of 224 anaemic paediatric patients who received myelosupressive chemotherapy for nonmyeloid malignancies (excluding brain tumours).   - *Intervention group:* n = 111 - *Control group:* n = 111   Age:   - *Intervention group:*   Mean (SD): 12.4 (3.6), Range 5-18   - *Control group:*   Mean (SD): 10.8 (4.0), Range 5-18  Sex:   - *Intervention group:*   M: 63 (56.8%), F: 48 (43.2%)   - *Control group:*   M: 58 (52.3%), F: 53 (47.7%)  Other: Tumour Type:   - *Intervention group:*   Solid Tumour: 41 (36.9%)  Hodgkin’s disease 16 (14.4%)  ALL: 40 (36.0%)  Non-Hodgkin’s lymphoma 14 (12.6%)   - *Control group:*   Solid Tumour: 57 (51.4%)  Hodgkin’s disease 11 (9.9%)  ALL: 35 (31.5%)  Non-Hodgkin’s lymphoma 8 (7.2%) | Type of intervention:  EPO was administered intravenously once per week, starting a dose of 600 units/kg and was increased to 900 units/kg if Hb had not increased by 1 g/dL or more from baseline by first follow-up visit. Red Blood Cell (RBC) transfusion was suggested when Hb was 7 g/dL or less.  Type of control:  Placebo was administered intravenously once per week. RBC transfusion was suggested when Hb was 7 g/dL or less. | Outcome definitions:  **HRQOL – Health related quality of life**   - PedsQL- GCS: QoL was measured using a 100-point scale by assessing physical, emotional, social and school functioning. Higher scores indicate higher QoL - PEDsQL3.0 Cancer Module: was measured using a 100-point scale assessing pain/hurt, nausea, procedural anxiety, treatment anxiety, worry, cognitive problems and communication. Higher scores indicate higher QoL - Parent QoL was measured using parent versions of PedsQL- GCS and PEDsQL3.0 Cancer Module. 100-point scale. Higher scores indicate higher QoL   **Haemoglobin level:** Mean Hb change from baseline to end/study in g/dL  **Blood transfusion:** Number of patients who required blood transfusions; Median time first transfusion  **Safety:** Occurrence of adverse events (hypertension)  Results (per outcome)  **HRQOL – Health related quality of life**  *Total PedsQL-GCS scores at final visit (intervention vs control):*   - Mean (SD) :74.9 (15.22) vs. 75.5 (15.74 - Group difference: -0.61 (95%CI -4.62 – 3.39), p = 0.823   *Mean (SD) of PEDsQL3.0 Cancer Modules at final visit (intervention vs control):*  Pain/hurt:   - Mean (SD): 73.1 (23.71) vs 75.7 (24.70). - Group difference: -2.64 (95%CI, -8.87 – 3.58), p = 0.215   Nausea:   - Mean (SD) 68.8 (20.11) vs. 72.0 (20.96). - Group difference: -3.19 (95%CI -8.51 – 2.13), p = 502   Procedural anxiety:   - Mean (SD): 75.2 (23.94) vs 76.4 (24.87). - Group difference: -1.16 (95%CI -7.47 – 5.15), p =0.940   Treatment anxiety:   - Mean (SD): 87.0 (15.07) vs 89.4 (15.66). - Group difference: -2.35 (95%CI -6.31 – 1.62), p=0.673   Worry:   - Mean (SD): 74.7 (22.21) vs 77.8 (23.18). - Group difference: -3.09 (95%CI -8.93 – 2.76), p = 0.360   Cognitive problems:   - Mean (SD) 81.8 (16.60) vs 80.4 (17.40). - Group difference 1.38 (95%CI -3.05 – 5.82), p=0.476   Perceived physical appearance:   - Mean (SD): 84.9 (16.82) vs 84.2 (17.46). - Group difference: 0.79 (95%CI -3.69 – 5.26), p=0.977   Communication:   - Mean (SD): 86.6 (16.58) vs 85.5 (17.30. - Group difference: 1.13 (95%CI -3.24 – 5.50), p=0.359   **Haemoglobin level**  Hb change from baseline to end/study (intervention vs control)   - Mean (SD): 1.3 (2.38) vs 1.0 (1.90) - Group difference; 0.37 (95%CI -0.11 – 0.84), p = 0.002   **Blood transfusions**  Number (%) of patients (intervention vs control): 72 (64.9%) vs 86 (77.5%)  Median time first transfusion (intervention vs control): 15 vs 14.5 days, p=0.254).  After 4 weeks patients were more likely to remain transfusion-free.  **Haemoglobin levels and quality of life**  A significant correlation was found between change in Hb level and change in quality-of-life score in the intervention group (r = 0.242; p = 0.018. In the placebo group the correlation was not significant (r = 0.86, p = 0.430).  **Safety**  Hypertension was reported in 2 (1.8%) patients in the intervention group and 1 (0.9%) patient in the placebo group.  At least one thrombotic vascular event (intervention vs control): 22.3% vs 22.7%)  Serious adverse event rates were similar in intervention and control (68.8% vs. 74.5%)  Serious adverse events in intervention group (experienced by more than 5% of the patients) included fever (11.6%), infection (6.3%),  Serious adverse events in control group (experienced by more than 5% of the patients) included infection (12.7%), fever (10.0%) and mucositis (5.5%).  Four patients died during the study, but no deaths were considered related to the study treatment. | Strengths:  Large-scale placebo-controlled study.  Limitations:  Inadequate utilization of iron supplementation in this study may have impaired the response to Epoetin Alfa. Investigators used clinical judgment to identify patients with iron-deficiency anaemia and exclude them from the study, but patients with a low iron level could enrol if the investigator thought it did not contribute to the anaemia.  **Risk of bias**  A. Selection bias:  Unclear  Reason: patients were randomly assigned to either intervention or control group. Patients were randomly assigned in a 1:1 ratio in groups of four patients. Allocation concealment was not reported.  B. Attrition bias:  Low risk  Reason: Loss of follow-up/dropout was less than 10%    C. Performance bias  Unclear  Reason: Blinding of patients and personnel was not reported in the study  D. Detection bias  Unclear  Reason: Blinding of outcome assessors was not reported in the study |

## *Sub-WG 1F Skin Complaints*

| **Pharmacological treatment of itching (pruritus)** | | | | |
| --- | --- | --- | --- | --- |
| ***Maxwell LG et al.*** The effects of a Small-Dose Naloxone Infusion on Opioid-Induced Side Effects and Analgesia in Children and Adolescents Treated with Intravenous Patient-Controlled Analgesia: A Double-Blind, Prospective, Randomized, Controlled Study. Anesth Analg 2005; 100:953–8 | | | | |
| **Study characteristics** | **Patient characteristics** | **Intervention / Control** | **Outcomes / Results** | **Comments**  **Risk of bias** |
| Type of study:  Double-Blind, Prospective, RCT  Setting:  1 centre, USA  Duration:  Pain and opioid induced side effects were monitored every 4h during the first 24h after surgery  Study years:  Not reported  Protocol published in register:  Not reported | Number and type of participants:  Total 46 pediatric patients, with acute, moderate to severe, postoperative pain. Surgical procedures included major orthopaedic, neurosurgical, or pectus excavatum surgery/   - Intervention group: n = 20 - Control group: n = 26   Age:   - Intervention group:   Mean (SD): 13.7 (2.7), Range 6-18   - Control group:   Mean (SD): 13.7 (2.3), Range 6-18  Sex:   - Intervention group:   M: 10 (50.0%), F: 10 (50.0%)   - Control group:   M: 11 (42.3%), F: 15 (57.7%)  There were no differences in the demographic data between the groups. | After surgery all patients were started on intravenous pump cassette which contained 100g of morphine sulfate in 100ml normal saline (1mg/mL). The following routine settings were established:   - Initial dose of up to 100µg/kg or more and - Maintenance basal infusion rate of 20 µg · kg^-1^ · h^-1^, - Demand dose of 20µg/kg, Lockout time interval of 8min - Maximum of five doses per hour.   Type of intervention:  The intervention group received 0.25 µg · kg^-1^ · h^-1^ of naloxone by continuous infusion. The naloxone was administered by a continuous infusion pump ‘piggy-backed’ into the patient’s catheter. The naloxone solution was prepared in the pharmacy by mixing 2mg of naloxone in 250mL of 0.9% saline (final concentration = 8 µg/mL).  Type of control:  The placebo group received only saline by the infusion pump. The study solutions were prepared by the pharmacist and diluted in saline to produce equal volumes to ensure proper blinding. | Outcome definitions:  Incidence and severity of pruritus  Incidence and severity of nausea  Incidence and severity of vomiting  Incidence of respiratory depression  Mean (SD) pain scores at rest  Mean (SD) pain scores with activity  Results (per outcome) – (Placebo vs intervention)  Incidence and severity of pruritus: 77% vs 20%, p < 0.05.  Incidence and severity of nausea: 70% vs 35%, p <0.05.  Incidence and severity of vomiting: 46% vs 25%, not significant  Incidence of respiratory depression: 0  Mean (SD) pain scores at rest: 4 (2) vs 3 (2), not significant  Mean (SD) pain scores with activity 6 (2) vs 6 (2), not significant | Strengths:  Double-blinded, prospective, randomized placebo-controlled study.  Limitations:  Only one concentration of naloxone was evaluated  Some side effects associated with opioid administration (urinary retention, constipation) could not be evaluated.  **Risk of bias**  A. Selection bias:  Low risk  Reason: Patients were randomly assigned by the hospital’s investigational drug pharmacy, using computer-generated random numbers. Patient, patient’s family, anaesthesiologist, pediatric pain service, nursing staff and observers all unaware of randomization.  B. Attrition bias:  Low risk  Reason: Outcome was assessed for 100% of the intervention group and 89% of the placebo group (dropout, n = 3)    C. Performance bias  Low risk  Reason: Participants and personnel were blinded from knowledge of which intervention was received.  D. Detection bias  Unclear  Reason: Blinding of outcome assessors was not reported in the study |

## *Sub-WG 1G Nausea and Vomiting*

| **Non pharmacological treatment of nausea and vomiting - Self-hypnosis** | | | | |
| --- | --- | --- | --- | --- |
| ***Jacknow DS et al.*** Hypnosis in the prevention of chemotherapy-related nausea and vomiting in children: a prospective study. J Dev Behav Pediatr 1994;15(4):258-64 | | | | |
| **Study characteristics** | **Patient characteristics** | **Intervention / Control** | **Outcomes / Results** | **Comments**  **Risk of bias** |
| Type of study:  Prospective, single-blind RCT  Setting:  2 centres, USA  Duration:  Study outcomes were assessed during first two courses of chemotherapy and 1 to 2 months and 4 to 6 months after diagnosis  Study years:  October 1990 – January 1992  Protocol published in register:  Not reported | Number and type of participants:  Total of 20 newly diagnosed children with cancer.   - Intervention group: 10 - Control group: 10   Age:   - Intervention group:   Mean: 11.9, Range: 6-15 yr.   - Control group:   Mean: 12.2, Range 7-18 yr.  Sex:   - Intervention group:   M: 5 (50%), F: 5 (50%)   - Control group:   M: 5 (50%), F: 5 (50%)  Duration of chemotherapy (course 1)   - Intervention group:   Mean: 3.5 days, Range (1-6 days)   - Control group:   Mean: 2.7 days, Range (1-5 days)  Duration of chemotherapy (course 2)   - Intervention group:   Mean: 2.6 days, Range (1-6 days)   - Control group:   Mean: 1.8 days, Range (1-5 days)  No significant differences between groups for all variables mentioned above.  Diagnosis   - Intervention group:   Leukaemia: 20%, Hodgkin’s lymphoma: 40%, Solid tumours: 40%   - Control group:   Leukaemia: 30%, Hodgkin’s lymphoma: 40%, Solid tumours: 30% | Type of intervention:  Children were taught self-hypnosis by a therapist in two to three sessions during the initial course of chemotherapy, using standard hypnotic techniques.  Hypnosis procedure was geared to the developmental level of the child, emphasis was placed on active involvement of imagination.  Sessions were 45 minutes long. Children were told to practice twice daily.  Children used the same anti-emetics as the control group but received no standard doses. Anti-emetics were only used if necessary.  Type of control:  Children in the control group received an equivalent amount of individual time consisting of informal conversation with the therapist.  A single therapist provided all hypnosis training and individualized time.  Patients in the control group were al on standard anti-emetic regimen:   - First line anti-emetics (until April 1991), thiethylperazine/ chloropromazine (until April 1991), with diphenhydramine. - First line anti-emetics (from May 1991): Ondansentron - Second line ant-emetics, metoclopramide with diphenhydramine   Patients received a dose of antiemetic medication at time 0 of chemotherapy, at 4 to hours of chemotherapy and sometimes at 8 to 12 hours of chemotherapy. Thereafter, anti-emetics were delivered every 4 to 6 hours if necessary. | Outcome definitions:  **Use of anti-emetic medication** Supplemental anti-emetic usage.  Medical records were reviewed daily for antiemetic medication usage. Standard doses given to the control group were subtracted from the total medication usage, leaving only p.r.n (pro re nata) antiemetic usage as the outcome variable  **Mean nausea and vomiting score**  Patient and parent reported nausea and vomiting were assessed at a standard time each day during the chemotherapy course using to instruments   - *Severity of nausea:* This was assessed using a graphic rating scale (five faces with expressions ranging from smiling to frowning) - *Frequency of vomiting and/or retching:* This was assessed using a 9-point Likert scale ranging from ‘none’ to ‘all the time’   As patient and parents report on all nausea and vomiting measures were highly correlated (r = 0.72 to r = 0.93; P <0.001) only patient scores were used.  Correlations for nausea and vomiting scores were high within each course of chemotherapy (r = 0.73 to 0.76, p = 0.001). Therefore, nausea and vomiting variables at each course of chemotherapy were standardized and combined into a single score for data analysis.  **Mean anticipatory nausea and vomiting** (assessed at ½ months and 4/6 months after diagnosis.  Three components of nausea were assessed   - *Severity of nausea* - *Frequency of nausea* - *Time of onset of nausea before chemotherapy*   Correlations between the three components of anticipatory symptoms were 0.78 to 0.97n (p < 0.001) Therefore the three scores were standardized and summed into an index of severity of anticipatory nausea. To eliminate negative numbers a constant of 2 was added to the scores.  **Mean anticipatory vomiting** (assessed at 1/2 months and 4/6 months after diagnosis.  Two components of vomiting were assessed   - Frequency of vomiting - Time of onset before vomiting   Because of small sample at 1/2 months and 4/6 months statistical analysis was not performed.  Results (per outcome)  **Use of anti-emetic medication**  The intervention group used significantly less supplemental anti-emetic medication   - *Course 1 of chemotherapy (intervention vs control):*   Mean (SD): 0.17 (0.33) vs 1.01 (1.33), p <0.04   - *Course 2 of chemotherapy (intervention vs control):*   Mean (SD): 0.34 (0.93) vs 2.10 (2.66), p<0.02  **Mean nausea and vomiting score**   - *Course 1 of chemotherapy (intervention vs control):*   Mean (SD): 1.79 (1.77) vs 3.21(2.01), p = NS   - *Course 2 of chemotherapy (intervention vs control):*   Mean (SD): 1.82 (2.01) vs 3.18 (1.81), p = NS  **Anticipatory nausea**   - *1 to 2 months post diagnosis (intervention vs control):*   Mean (SD): 0.82 (2.60) vs 3.17 (2.60), p<0.013   - *4 to 6 months post diagnosis (intervention vs. control):*   Mean (SD): 1.69 (3.64) vs 2.54 (2.47), p = NS  **Anticipatory vomiting**  Two patients in the control group experienced anticipatory vomiting vs zero patients in the intervention group | Strengths:  Limitations:   - Differences in supplemental anti-emetic medication usage could have been affected by the potential difference in expectation regarding antiemetic use. Patients in the intervention group may have believed they had failed if they requested antiemetic medication. - Relatively low number of patients included in the study - Possibility of selection bias because subjects were matched on age and emetogenicity of chemotherapy.   **Risk of bias**  A. Selection bias:  Unclear  Reason: Subjects underwent stratified random assignment. Patients were matched on age and emetogenicity of their chemotherapeutic regimens. Allocation concealment was not reported  B. Attrition bias:  Low risk  Reason: Outcomes of all patients included in the study were assessed    C. Performance bias  High risk  Reason: Both participants and personnel were not blinded from knowledge of which intervention was received  D. Detection bias  Unclear  Reason: Blinding of outcome assessors was not reported |

| **Pharmacological treatment of nausea and vomiting - low dose ondansetron vs high dose ondansetron** | | | | |
| --- | --- | --- | --- | --- |
| ***Brock P et al. An*** increased loading dose of ondansetron: a north european, double-blind randomised study in children, comparing 5 mg/m2 with 10 mg/m2. Eur J Cancer 1996 Sep;32A(10):1744-8 | | | | |
| **Study characteristics** | **Patient characteristics** | **Intervention / Control** | **Outcomes / Results** | **Comments**  **Risk of bias** |
| Type of study:  Double-blind RCT  Setting:  18 oncology units in Belgium, the Netherlands, Denmark, Sweden and Finland.  Duration:  Follow-up during the whole chemotherapy course.  Study years:  November 1992 – June 1994  Protocol published in register:  Not reported | Number and type of participants:  A total of 187 children who had not received prior chemotherapy and who were scheduled to receive highly emetogenic chemotherapy.  *Number of patients at baseline*  Group 1: 93, Group 2: 94  *Number of patients with outcome measured*  Outcome was measured in 158 children. 27 children were excluded due to protocol violation, 2 dropouts.  Group 1: 79, Group 2: 79   - *Number of patients receiving cisplatin chemotherapy*   Group 1: 14, Group 2: 17   - *Number of patients receiving ifosfamide*   Group 1: 14, Group 2: 14   - *Number of patients with treatment failures*   Group 3: 15, Group 4: 19  Age (at baseline):   - Group 1 - 5mg/m^2^ ondansetron: Mean: 8.4 yrs., Range: 2 – 16.7 yrs. - Group 2- 10mg/m^2^ ondansetron: Mean 8.5 yrs., 1.9 – 16.3 yrs.   Sex (at baseline):   - Group 1 - 5mg/m^2^ ondansetron: M: 50 (54%), F: 43 (46%) - Group 2- 10mg/m^2^ ondansetron:   M: 52 (55%), F: 42 (45%)  Mean surface area (m^2^) (at baseline)   - Group 1 - 5mg/m^2^ ondansetron: 1.1 m^2^ - Group 2- 10mg/m^2^ ondansetron:   1.1 m^2^ | Group 1: 5mg/m^2^ ondansetron  Intravenous intake: The initial intravenous loading-dose of ondansetron 5mg/m^2^ (maximum 8 mg) was administered immediately prior to chemotherapy as a 15 min infusion. Two additional intravenous doses of ondansetron were administered 8 and 16 h after the initial dose.  Oral intake: on subsequent days when chemotherapy was given, ondansetron was administered orally three times a day at a dose according to the surface area of the child: 4mg < 1 m^2^, 8 > 1m^2^. The first intake was given 24h after the start of chemotherapy and it was continued for 3 days after the last day of chemotherapy or 5 days if nausea or vomiting persisted.  Group 2: 10mg/m^2^ ondansetron  Initial intravenous loading-dose of ondansetron was 10mg/m^2^ (maximum of 16mg).  The rest of the procedure regarding intravenous and oral intake were similar to group 1.  *Treatment failures*  Only patients were included that were considered treatment failures: Patients suffered more than five emetic episodes in any 24-h period during their first course of chemotherapy, patients received rescue medication and/or there was any change in anti-emetic drug treatment.  Group 3 treatment failures 10mg/m^2^ dexamethasone + 5mg/m^2^ ondansetron  Patients were given dexamethasone at a dose of 10mg/m^2^ (maximum 16 mg) as an intravenous infusion over 15 mg, 30 in prior the chemotherapy, in addition to ondansetron. Loading-dose of ondansetron was the same as in the first course of chemotherapy, 5mg/m^2^.  Group 4 treatment failure - 10mg/m^2^ dexamethasone + 10mg/m^2^ ondansetron  Patients were given dexamethasone at a dose of 10mg/m^2^ (maximum 16 mg) as an intravenous infusion over 15 mg, 30 in prior the chemotherapy, in addition to ondansetron. Loading-dose of ondansetron was the same as in the first course of chemotherapy, 10 mg/m^2^ | Outcome definitions:  **Anti-emetic efficacy (first 24 hr)**  Anti-emetic efficacy of the two loading doses of ondansetron was analysed during the first 24h of chemotherapy by comparing   - the percentage of complete or major responders - mean number of emetic episodes - grade of nausea   *Categories*   - Complete/none: No emetic episode/not feeling sick at all - Major/mild: 1-2 emetic episodes/feeling sick   *Emetic episode (vomiting/retching):* A single vomit or retch or any number of continuous vomits or retches. Each emetic episode was separated by the absence of vomiting or retching for at least 1 minute.  *Categories for emetic efficacy:*   - Complete response: No emetic episode - Major response: 1-2 emetic episodes - Minor response: 3-5 emetic episodes - Failure: more than 5 emetic episodes   *Nausea:* feeling of wanting to be sick without retching.   - None: not feeling sick at all - Mild: feeling sick - Severe: feeling very sick   *Appetite*  *Grading of appetite:* better than usual, as usual, worse than usual  Results (per outcome)  *All patients*  **Anti-emetic efficacy**  Anti-emetic efficacy over the first 24h of chemotherapy.   - Percentage of patients with two or fewer emetic episodes (group 1 vs group 2): 71% vs 72%, p = NS. - Percentage of patients with no or mild nausea (group 1 vs group 2): 90% vs 86%, p = NS. - Percentage of patients with usual or better appetite: 44-45%   Anti-emetic efficacy on the worst day   - Percentage of patients with two or fewer emetic episodes (group 1 vs group 2): 61% vs 60%, p = NS. - Percentage of patients with no or mild nausea (group 1 vs group 2): 80% vs 70%, p = NS. - Percentage of patients with usual or better appetite: 27-28%   *Cisplatin Chemotherapy*  Anti-emetic efficacy over the first 24h of chemotherapy.   - Percentage of patients with two or fewer emetic episodes (group 1 vs group 2): 50% vs 53%, p = NS. - Percentage of patients with no or mild nausea (group 1 vs group 2): 100% vs 86%, p = NS.   *Ifosfamide*   - Percentage of patients with two or fewer emetic episodes (group 1 vs group 2): 79% vs 64%, p = NS. - Percentage of patients with no or mild nausea (group 1 vs group 2): 78% vs 77%, p = NS   *Treatment failures*  **Anti-emetic efficacy**   - Percentage of patients with two or fewer emetic episodes (group 3 vs group 4): 60% vs 60%, - Percentage of patients with no or mild nausea (group 3 vs group 4): 60% vs 60%, - Percentage of patients with usual or better appetite (group 3 vs group 4): 60% vs 72%. | Strengths:  Limitations:  Definition of the worst day is not reported in the article.  Good control of emesis and nausea was defined as patients having 2 or less emetic episodes and patients reporting none to mild patients. However, it is not reported where this definition of good control is based on.  **Risk of bias**  A. Selection bias:  Low risk  Reason: Patients were randomized according to randomisation code.  B. Attrition bias:  High risk  Reason: Outcome was measured in 160 children. 27 children were excluded due to protocol violation. Outcome was assessed for more than 90% in each treatment arm.    C. Performance bias  Low risk  Reason: the anti-emetic loading dose of ondansetron was blinded to the clinicians, the patients, the parents and the nurses.  D. Detection bias  unclear  Reason: not reported if outcome assessors were blinded |

| **Pharmacological treatment of nausea and vomiting - low dose ondansetron vs high dose ondansentron vs placebo** | | | | |
| --- | --- | --- | --- | --- |
| ***Parker RI et al***. Randomized, double-blind, crossover, placebo-controlled trial of intravenous ondansetron for the prevention of intrathecal chemotherapy-induced vomiting in children. Biol Blood Marrow Transplant 1999;5(6):386-93 | | | | |
| **Study characteristics** | **Patient characteristics** | **Intervention / Control** | **Outcomes / Results** | **Comments**  **Risk of bias** |
| Type of study:  Randomized, Double-Blind Crossover, Placebo-controlled study  Setting:  1 centre, USA  Duration:  24h after treatment  Study years:  Not reported  Protocol published in register:  Not reported | Number and type of participants:  A total of 26 children with newly diagnosed acute lymphoid or nonlymphoid leukaemia.  Each patient acted as their own control.   - Intervention group: 26 - Control group: 26   Age:   - Intervention group:   Mean: 6yr, Range: 2-17 yr.   - Control group:   Mean: 6yr, Range: 2-17 yr.  Sex:   - Intervention group:   M: 12 (46.2%), F: 14 (53.8%)   - Control group:   M: 12 (46.2%), F: 14 (53.8%)  Total intrathecal treatments:  146 intrathecal treatments; mean 5.62/patient; range 3-7   - 51 placebo treatments - 47 low dose ondansetron - 48 high dose ondansetron   Total vomiting episodes:  52 vomiting episodes; mean 2.0/patient; range: 0-7 patient | Each patient acted as his or her control; treatments (placebo, low-dose ondansetron, high-dose ondansetron) were administered in random order for up to 6 intrathecal treatments. During the first three treatments, each patient would receive each of the interventions one time.  Intervention 1: Low dose ondansetron  Ondansetron at 0.15 mg/kg (low dose) by a 15-minute intravenous infusion 30 minutes before undergoing a lumbar puncture for the administration of intrathecal chemotherapy.  Patients who had two or more vomiting episodes after the intrathecal chemotherapy would then receive antiemetic therapy with diphenhydramine HCl, Prochlorperazine, or trimethobenzamide HCl.  Intervention 2: High dose ondansetron:  Ondansetron at 0.45 mg/kg (high dose). Procedure is the similar to the procedure in the other groups  Placebo  Patients received normal saline (placebo). Procedure is the similar to the procedure in the other groups | Outcomes  **Treatments with vomiting episodes in 24h**   - Percentage of treatments with vomiting episodes - RR of vomiting   Results (per outcome)  **Treatments with vomiting episodes in 24-h**  N (%) of patients: 23 (88.5%)  Percentage of treatments with vomiting (vs placebo)   - Total: 35.6% vs 62.7%, - Low dose ondansetron 27.7% vs 62.7%, p<0.001 - High dose ondansetron: 14.6% vs 62.7%, p<0.001 - Any dose ondansetron: 21.1% vs 62.7%, p<0.001   Percentage of treatments with vomiting (vs low dose ondansetron)   - High dose ondansetron: 14.6% vs 27.7 %, p<0.1   RR of vomiting in the placebo group   - Placebo vs low dose ondansetron = 2.3 - Placebo vs high dose ondansetron = 4.3 - Placebo vs any dose ondansetron = 3.0   Reduction of RR by pre-administrating ondansetron: 65.7%  **Treatments with ≥ 2 vomiting episodes**  N (%) of patients: 17 (65%)  Percentage of treatments with vomiting (vs placebo)   - Total: 21.2% vs 43.1%, - Low dose ondansetron 12.8% vs 43.1%, p<0.001 - High dose ondansetron: 6.3% vs 43.1%, p<0.001 - Any dose ondansetron: 9.5% vs 43.1%, p<0.001   Percentage of treatments with vomiting (vs low dose ondansetron)   - High dose ondansetron: 6.3% vs 12.8 %, p<0.3   RR of vomiting in the placebo group   - Placebo vs low dose ondansetron = 3.4 - Placebo vs high dose ondansetron = 6.8 - Placebo vs any dose ondansetron = 4.5   Reduction of RR by pre-administrating ondansetron: 77.5%  **Treatments with ≥ 4 vomiting episodes**  Percentage of treatments with vomiting (vs placebo)   - Total: 20.3% vs 25.5%, - Low dose ondansetron 4.3% vs 25.5%, p<0.005 - High dose ondansetron: 0.0% vs 25.5%, p<0.001 - Any dose ondansetron: 2.1% vs 25.5%, p<0.001   Percentage of treatments with vomiting (vs low dose ondansetron)   - High dose ondansetron: 0.0% vs 4.3 %, p<0.1   RR of vomiting in the placebo group   - Placebo vs low dose ondansetron = 5.8 - Placebo vs high dose ondansetron - Placebo vs any dose ondansetron = 12.1   Reduction of RR by pre-administrating ondansetron: 91.6%  **Incidence of vomiting (10 y or older vs younger than 10 y):**  19.0% vs 38.4%, p < 0.05 | Strengths:  Limitations:  Nausea is not studied  Small study population  **Risk of bias**  A. Selection bias:  Unclear  Reason: Patients were randomly assigned to receive one of three interventions in a double-blinded fashion. allocation concealment was not reported  B. Attrition bias:  Low risk  Reason: One child was withdrawn from the study. Outcomes were assessed for more than 90% of the study population    C. Performance bias  Low risk  Reason: Participants and personnel were blinded from knowledge of the intervention received, as the study was double-blinded.  D. Detection bias  Unclear  Reason: Blinding of outcome assessors was not reported |

| **Pharmacological treatment of nausea and vomiting - granisetron vs ondansetron** | | | | |
| --- | --- | --- | --- | --- |
| ***Orchard PJ et al.*** A prospective randomized trial of the anti-emetic efficacy of ondansetron and granisetron during bone marrow transplantation. J Dev Behav Pediatr 1994;15(4):258-64 | | | | |
| **Study characteristics** | **Patient characteristics** | **Intervention / Control** | **Outcomes / Results** | **Comments**  **Risk of bias** |
| Type of study:  Prospective randomized trial  Setting:  1 centre, USA  Duration:  Outcomes were measured from the first day of the preparative regimen through day 2 (0 – 48h)  Study years:  Not reported  Protocol published in register:  Not reported | Number and type of participants:  A total of 187 patients 2-65 years of age undergoing hematopoietic cell transplantation, patients were not being treated with anti-emetic medications and were not having a history of recent emetic episodes preceding conditioning therapy.   - Granisetron: 90 - Ondansetron: 97   Age:   - Granisetron:   Median: 41 yrs., Range: 3-62 yr.  N (%) <18 yrs.: 23 (26%)  N (% ≥ 18 yrs.: 67 (74%)   - Ondansetron:   Median: 36 yrs., Range: 5-62  N (%) <18 yrs.: 28 (29%)  N (% ≥ 18 yrs.: 69 (71%)  Sex:   - Granisetron:   M: 54 (60%), F: 36 (40%)   - Control group:   M: 53 (55%), F: 44 (45%)  Type of transplant   - Granisetron:   Autologous N (%): 34 (38%)  Allogeneic N (%): 24 (27%)  Unrelated N (%): 32 (35%)   - Control group:   Autologous N (%): 34 (35%)  Allogeneic N (%): 27 (28%)  Unrelated N (%): 36 (37%) | Granisetron  A single intravenous dose of granisetron was given before the start of chemotherapy or total body irradiation (TBI) followed by intermittent intravenous dosing of granisetron every 12 hours.  Patients received a placebo consisting of a continuous infusion of 5% dextrose.   - *Patients < 18 yrs.:* Patients received a 10µg/kg/dose every 12 hours. - *Patients ≥ 18 yrs.:* Patients received a 7.5µg/kg/dose (0.5mg for a 70 kg patient) every 12 hours.   Ondansetron  Patients received an initial loading dose of ondansetron before the start of the first dose of chemotherapy or TBI, followed by continuous infusion.  A placebo consisting of an intermittent dose of 5% dextrose was administered every 12 hours.   - *Patients < 18 yrs.:* Patients received a 0.15 mg/kg load along with a 0.03mg/kg/h drip rounded to the nearest 0.1 mg - *Patients ≥ 18 yrs.:* Patients received an 8 mg load followed by a 0.015 mg/kg/h drip rounded to the nearest 0.5mg/h, amounting to 24 mg/day for a 70kg individual.   All patients  Received dexamethasone   - *Patients < 18 yrs.:* 10 mg/m^2^/day - *Patients ≥ 18 yr:*10mg/day   For breakthrough nausea and vomiting additional medications were available on request, lorazepam, prochlorperazine or promethazine. | Outcome definitions:  **Emetic episodes**  Expulsion of stomach contents separated by 1 minute from a previous episode  **Retching**  Non-productive emptying of stomach contents. A series of retches lasting <5 minutes was considered one emetic episode.  **Nausea**  A visual analogue scale (smiling or frowning faces) was used to determine severity of nausea, score ranging from 0 (no nausea to 5 (worst nausea ever experienced), higher score indicating higher severity of nausea  **Control of emesis**   - Complete control: no emetic episodes - Major control: one to two emetic episodes in 24 hours - Minor control: three to five emetic episodes in 24h - Treatment failure: more than five emetic episodes in 24hrs, administration of more than two doses of rescue drugs per day.   Results (per outcome)  **mean (9%%CI) emetic episodes per day**   - *Granisetron vs ondansetron:* 0.73 (95%CI 0.55-1.91) vs 0.86 (95%CI 0.67-1.05), p = 0.32   - *Age <18 yrs.*: 0.54 (95%CI 0.27-0.81) vs 0.87 (95%CI 0.63-1.11), p = 0.08   - *age ≥ 18 yrs.:* 0.80 (95%CI 0.57-1.03) vs 0.86 (95%CI 0.63-1.09), p = 0.71 - *Female vs Male:*   0.97 (95%CI 0.63-1.30) vs 0.69 (95%CI 0.52-0.86), p = 0.08   - *Age <18 yrs. vs age ≥ 18 yrs.:*   0.82 (95%CI 0.47-1.17) vs 0.88 (95%CI 0.59-1.16), p = 0.71   - *TBI vs Chemotherapy alone:*   0.73 (95%CI 0.73 (0.56-0.89) vs 1.06 (95% CI 0.77-1.34), p = 0.04  **Nausea score, mean (9%%CI)**   - *Granisetron vs ondansetron:* 1.17 (95%CI 1.00-1.34) vs 1.29 (95%CI 1.12-1.45), p = 0.32   - *Age <18 yrs.*: 0.82 (95%CI 0.55-1.09) vs 1.14 (95%CI 0.90-1.38), p = 0.09   - *age ≥ 18 yr1.29* (95%CI 1.09-1.49) vs 0.1.36 (95%CI 1.15-1.56), p = 0.65 - *Female vs Male:*   1.63 (95%CI 1.34-1.92) vs 01.31 (95%CI 1.06-1.26, p <0.01   - *Age <18 yrs. vs age ≥ 18 yrs.:*   1.33 (95%CI 1.03-1.63)) vs 1.6 (95%CI 1.36-1.84), p = 0.05   - *TBI vs Chemotherapy alone:*   1.14 (95%CI 1.00-1.29)) vs 1.33 (95% CI 1.07-1.60), p = 0.2  **Control of emesis (granisetron vs. ondansetron)**  Percentage of days with complete control of emesis: 63% vs 61%, p = 0.68  Percentage of days with major control of emesis: 27% vs 27%  Percentage of days with minor control of emesis: 7% vs 8%  Percentage of days with treatment failure: 3% vs 4%  **Safety**  Both drugs were well tolerated. In one case granisetron was discontinued because of headaches. | Strengths:  In addition to the randomization between granisetron and ondansetron a stratification was performed based on age,  Limitations:  **Risk of bias**  A. Selection bias:  Unclear  Reason: The study was designed as a double-blind, randomized trial, in which patients received either granisetron or ondansetron 30 minutes before initiation of the ablative regimen. Allocation concealment was not reported.  B. Attrition bias  Low risk  Reason: Outcome was assessed for 100% of the population in each treatment arm.    C. Performance bias  Low risk  Reason: The study was designed in a double-blind fashion  D. Detection bias  Unclear  Reason: Blinding of outcome assessors was unclear. |

| **Pharmacological treatment of nausea and vomiting - Ondansetron vs metoclopramide** | | | | |
| --- | --- | --- | --- | --- |
| ***Kóseoglu V et al.*** Comparison of the efficacy and side-effects of ondansetron and metoclopramide-diphenhydramine administered to control nausea and vomiting in children treated with antineoplastic chemotherapy: a prospective randomized study. Eur J Pediatr 1998 Oct;157(10):806-10 | | | | |
| **Study characteristics** | **Patient characteristics** | **Intervention / Control** | **Outcomes / Results** | **Comments**  **Risk of bias** |
| Type of study:  A prospective randomized study  Setting:  1 centre, turkey  Duration:  24-hour follow-up, every day until 5 days after chemotherapy.  Study years:  Not reported  Protocol published in register:  Not reported | Number and type of participants:  A total of 15 patients diagnosed with a malignant disease excluding CNS involvement, gastro-intestinal tract obstruction or any accompanying disease were evaluated.  A total of 64 chemotherapy courses were given to the patients.  Age:  Mean age: 7.6 yrs.  Sex:  M: 9 (60%), F: 6 (40%)  Other  There was differentiated between therapies that included cisplatin.   - Ondansetron: 9 chemotherapy courses with cisplatin, 23 chemotherapy courses non- cisplatin - Metoclopramide: 9 chemotherapy courses with cisplatin, 23 chemotherapy courses non-cisplatin | Ondansetron  Ondansetron was administered at a dose of 5mg/m^2^ intravenously (maximum 8mg) 15 min before the chemotherapy and was continued orally (4mg/m2 per day) twice a day for 5 days.  Metoclopramide  Metoclopramide (1mg/kg) was administered intravenously 30 min before the chemotherapy and continued orally (0.14 mg/kg per day) four times a day for 5 days. To prevent side effects, diphenhydramine (5mg/kg per day) was given orally for 5 days. | Outcome definitions:  **Vomiting attack**  A rejection or refusal of the content of the stomach. A vomiting attack recurring 1 min after the previous one, was accepted as a separate attack.  **Vomiting efficacy**   - Complete efficacy: No vomiting attack in the 24h follow up period, it was accepted as a complete efficacy. - Major efficacy: 1-2 vomiting attacks - Minor efficacy: 3-5 vomiting attacks - No efficacy: ≥ 5 vomiting attacks   **Nausea**  No nausea,  Mild nausea: without interfering with daily activities  Moderate nausea: moderately interfering with daily activities  Serious nausea: seriously interfering with daily activities.  Results (per outcome)  **Vomiting attack efficacy first 24h (ondansetron vs metoclopramide)**  *Cisplatin*  N with complete efficacy: 5 vs. 1, p < 0.05  N with major efficacy: 3 vs 1, p = ns  N with minor efficacy: 1 vs 3, p = ns  N with no efficacy: 0 vs 4, p = ns  *Non-cisplatin*  N with complete efficacy: 21 vs. 17, p < 0.05  N with major efficacy: 2 vs 1, p = ns  N with minor efficacy: 0 vs 1, p = ns  N with no efficacy: 0 vs 4, p = ns  **Vomiting attack in 2^nd^ -5^th^ day after chemotherapy (ondansetron vs metoclopramide**  N of courses in which there was a vomiting attack:  *Cisplatin*: 4 vs 8, p <0.05  *Non-cisplatin:* 2 vs 6, p = ns  **Nausea**  *Cisplatin*  N with no nausea: 7 vs. 0, p < 0.05  N with mild nausea: 1 vs 2, p = ns  N with moderate nausea: 1 vs 2, p = ns  N with serious nausea: 0 vs 5, p = ns  *Non-cisplatin*  N with no nausea: 22 vs. 19, p < 0.05  N with mild nausea: 1 vs 1, p = ns  N with moderate nausea: 0 vs 2, p = ns  N with serious nausea: 5 vs 1, p = ns  **Safety**  Side effects metoclopramide: extrapyramidal symptoms  Side effects ondansetron: headache | Strengths:  Limitations:  The study did not elaborate on the process of assigning patients to the ondansetron/metoclopramide group. It is expected that patients received a different medication each chemotherapy course, however this is not reported in the paper.  Small study population  **Risk of bias**  A. Selection bias:  High risk  Reason: The study did not report on how randomization took place.  B. Attrition bias:  Low risk  Reason: Outcome was assessed for all patients and chemotherapy courses.    C. Performance bias  High risk  Reason: Blinding from knowledge of which intervention was received was not reported in the study  D. Detection bias  Unclear  Reason: Blinding of outcome assessors was not reported. |

| **Pharmacological treatment of nausea and vomiting -Tropisetron vs Granisetron** | | | | |
| --- | --- | --- | --- | --- |
| ***Aksoylar S et al.*** Comparison of tropisetron and granisetron in the control of nausea and vomiting in children receiving combined cancer chemotherapy. Pediatr Hematol Oncol 2001 Sep;18(6):397-406. | | | | |
| **Study characteristics** | **Patient characteristics** | **Intervention / Control** | **Outcomes / Results** | **Comments**  **Risk of bias** |
| Type of study:  Prospective randomised study  Setting:  1 centre, turkey  Duration:  24-hour follow-up after chemotherapy  Study years:  Not reported  Protocol published in register: | Number and type of participants:  A total of 51 children receiving highly emetogenic chemotherapy were studied in 133 chemotherapy cycles. Emetogenic chemotherapy cycles were randomised (1:1) to receive either tropisetron or granisetron as an antiemetic agent.  Age:  Median: 6.5, Range: 1-17.  12 (23.5%) children were < 2 yrs. old  Sex:  M: 32 (62.7%), F: 19 (37.3%)  Diagnosis:  Lymphoblastic leukaemia: 43%  Lymphoma: 18%  Rhabdomyosarcoma: 8%  Acute myeloblastic leukaemia: 8%  Neuroblastoma: 6%  PNET and Ewing sarcoma: 6%  Wilm’s tumour: 4%  Germ cell therapy: 4%  Other: 3%  Chemotherapy:  Highly emetogenic chemotherapy (grade 3):  84/133 chemotherapy cycles (63%)  Very highly emetogenic chemotherapy (grade 4):  49/133 chemotherapy cycles (37%)  There was no significant difference of patient characteristics between tropisetron/granisetron groups. | Tropisetron:  A single daily dose of tropisetron of 0.2 mg/kg/day (max 5 mg) was given intravenously in saline, 30 min before cytotoxic drug administration. Tropisetron was administered each day the children received chemotherapy. No concomitant antiemetic therapy was given to the patients.  Granisetron  A single daily dose of granisetron 40 µg/kg/day (max 3 mg) was given intravenously in saline, 30 min before cytotoxic drug administration. Granisetron was administered each day the children received chemotherapy. No concomitant antiemetic therapy was given to the patients. | Outcome definitions:  **Vomiting efficacy**  A single episode of vomiting was defined as 1 event. 1 vomit is 1 emetic episode  Complete control: No emetic episode within 24hr  Partial control: 1-4 episodes within 24hr  Failure: > 4 emetic episodes within 24 hr  **Nausea**  Nausea continuing for 1 hour was defined as a single episode of nausea, regardless of severity.  Complete control: No episode of nausea within 24 hr  Partial control: 1-4 episodes of nausea within 24hr  Failure: > 4 episodes of nausea  **Overall response**  Complete control: no vomiting, no nausea  Partial control: 1-4 emetic episodes and/or 1-4 episodes of nausea  Failure >4 emetic episodes and/or >4 episodes of nausea  Results (per outcome)  **Acute Nausea and vomiting**  *Acute vomiting (tropisetron vs granisetron)*  Complete control: 74% vs 88%, p = 0.04  Partial control: 20% vs 12%  Failure: 6% vs 0%  *Acute nausea (tropisetron vs granisetron)*  Complete control: 56% vs 82%, p = 0.002  Partial control: 38% vs 18%  Failure: 6% vs 0%  **Overall response on the worst day (tropisetron vs granisetron)**  Complete control: 29% vs 55%, p = 0.007  Partial control: 62% vs 40%  Failure: 9% vs 5%  *Grade 3 chemotherapy (tropisetron vs granisetron)*  Complete control: 28% vs 67%, p = 0.002  Partial control: 64% vs 29%  Failure: 8% vs 4%  *Grade 4 chemotherapy (tropisetron vs granisetron)*  Complete control: 30% vs 32%, p = 0.7  Partial control: 60% vs 64%  Failure: 11% vs 4%  *Body weight < 25 ((tropisetron vs granisetron)*  Complete control: 45% vs 63%, p = 0.14  Partial control: 48% vs 37%  Failure: 7% vs 0%  *Body weight > 25 (tropisetron vs granisetron)*  Complete control: 18% vs 47%, p = 0.02  Partial control: 71% vs 44%  Failure: 11% vs 9%  **Adverse events**  Adverse events were reported in 9 (6%) of the chemotherapy cycles (p = NS)   - Headache (n = 6) - Constipation (n=2) | Strengths:  It was studied whether the efficacy of both tropisetron and granisetron was different depending on the emogenicity of the chemotherapy and body weight.  Limitations:  Definition of ‘worst day chemotherapy’ was not given.  **Risk of bias**  A. Selection bias:  Unclear  Reason: Patients receiving highly and very highly emetogenic chemotherapy cycles were randomised (1:1) to reive either tropisetron or granisetron as an antiemetic agent.  Allocation concealment was not reported  B. Attrition bias:  Low risk  Reason: Outcome was assessed for all patients and chemotherapy courses    C. Performance bias  High risk  Reason: Blinding from knowledge of which intervention was received was not reported in the study  D. Detection bias  Unclear  Reason: Blinding of outcome assessors was unclear |

| **Pharmacological treatment of nausea and vomiting - Aprepipant + Dexamethasone + ondansetron vs Dexamethasone + ondansetron** | | | | |
| --- | --- | --- | --- | --- |
| ***Gore L et al.*** Aprepitant in adolescent patients for prevention of chemotherapy-induced nausea and vomiting: a randomized, double-blind, placebo-controlled study of efficacy and tolerability. Pediatr Blood Cancer 2009; 52:242–247 | | | | |
| **Study characteristics** | **Patient characteristics** | **Intervention / Control** | **Outcomes / Results** | **Comments**  **Risk of bias** |
| Type of study:  Randomized, double-blind, placebo-controlled study  Setting:  12 sites, USA  Duration:  Outcomes were measured for 5 days after first chemotherapy infusion. 6-8 days patients were followed up in a clinic visit.  Study years:  April 2004 – September 2004  Protocol published in register: | Number and type of participants:  Total of 46 children with cancer   - Intervention group: 28 + 4 additional patients who received open-label aprepitant. - Control group: 18   Age:   - Intervention group:   Mean (SD): 15 (1.73), Range: 12-19 yr.   - Control group:   Mean (SD): 15 (1.91), Range: 11-17  Sex:   - Intervention group:   M: 24 (75%), F: 8 (25%)   - Control group:   M: 12 (66.6%), F: 6 (33.3%)  Most common diagnosis:   - Intervention group:   Bone sarcoma: 53.1%   - Control group:   Bone sarcoma 83.3%  There was no significant difference of patient characteristics between intervention/control groups. | Intervention - Aprepipant + Dexamethasone + ondansetron  Day 1:  Aprepitant 125 mg was administered 1 hr before chemotherapy.  Dexamethasone 8mg and ondansetron (0.15mg/kg x 3 doses) started 30 minutes before chemotherapy  Day 2: Dexamethasone 4mg, ondansetron 0.15 mg/kg x 3 doses), aprepitant 80mg  Day 3: Dexamethasone 4mg, aprepitant 80mg  Day 4: dexamethasone 4mg  Control – Dexamethasone + ondansetron  Day 1:  Placebo was administered 1 hr before chemotherapy.  Dexamethasone 16 mg and ondansetron (0.15mg/kg x 3 doses) started 30 minutes before chemotherapy  Day 2: Dexamethasone 8mg, ondansetron 0.15 mg/kg x 3 doses)  Day 3: Dexamethasone 8mg  Day 4: dexamethasone 8mg | Outcome definitions:  **Safety and Tolerability** Adverse events  **Efficacy:** Complete response: no vomiting and no use of rescue therapy  **Pharmacokinetics**  Results (per outcome):  **Adverse events (intervention vs control)**  *>1 clinical adverse event:* 27 (84.4%) vs 13 (172.2%)  *Drug related clinical adverse events (i.e., hiccups):* 7 (21.9%) vs 1 (5.6%)  *Serious clinical adverse events (i.e., neutropenia):* 10 (31.3%) vs 3 (16.7%)  *>1 laboratory adverse event (neutropenia, hypokalaemia, leukopenia):* 6 (18.8%) vs 6 (33.3%)  *No deaths, no discontinuation due to adverse events, no serious drug-related adverse events, no drug-related laboratory adverse events*  **Vomiting efficacy (intervention (n=28) vs control (n=18))**  *Proportion of patients with complete response*  Acute (0-24 hr):  60.7% (95%CI 40.6% - 78.5%) vs 38.9% (95%CI 17.3% - 64.3%)  Delayed (24-120 hr):  35.7% (95%CI 18.6% - 55.9%) vs 5.6% (95%CI 0.1% - 27.3%)  Overall phase (0-120 hr):  28.6% (95%CI 13.2% - 48.7%) vs 5.6% (95%CI 0.1% - 27.3%)  *Proportion of patients with no vomiting*  Acute (0-24 hr):  64.3% (95%CI 44.1% - 81.4%) vs 44.4% (95%CI 21.5 % - 69.2%)  Delayed (24-120 hr):  39.3% (95%CI 21.5% - 59.4%) vs 5.6% (95%CI 0.1% - 27.3%)  Overall phase (0-120 hr):  32.1% (95%CI 15.9% - 52.4%) vs 5.6% (95%CI 0.1% - 27.3%)  *Proportion of patients with no use of rescue therapy*  Acute (0-24 hr):  71.4% (95%CI 51.3% - 86.8%) vs 61.1 (95%CI 35.7% - 82.7%)  Delayed (24-120 hr):  50.0% (95%CI 30.6% - 69.4%) vs 27.8% (95%CI 9.7 % - 53.53%)  Overall phase (0-120 hr):  42.9% (95%CI 24.9% - 62.8%) vs 22.2% (95%CI 6.4% - 17.6%)  No nausea (Overall phase)  44.4% (95%CI 25.5% - 64.7%) vs 17.6% (95%CI 3.8% - 43.46%)  Although overlap of the exact 95% Cis was noted for all CR endpoints, response rates were numerically higher for the intervention group.  **Pharmacokinetics**  Pharmacokinetic parameters in 17 adolescent cancer patients were compared with data from 12 healthy adult subjects from a previous study of the same 3-day aprepitant dosing regimen as the current study.  Geometric mean ratio (Adolescent patients/healthy adults)  AUC0-24hr (ng/hr/ml): 0.81 (95%CI 0.63-1.06)  CMax (ng/ml): 0.78 (95%CI 0.61-1.00)  C24 hr (ng/ml): 0.83 (95%CI 0.57-1.20)  C48 hr (ng/ml): 0.67 (95%CI 0.38-1.19)  C72hr (ng/ml): 0.61 (95%CI 0.33-1.13)  The 90% CIs for the GMRs (adolescent/adult) forAUC0–24 h,  Cmax, C24 h, C48 h, and C72 h containe1.0, which suggested that age did not affect these parameters | Strengths:  Limitations:  Lack of statistical significance due to a small sample size.  **Risk of bias**  A. Selection bias:  Unclear  Reason: Eligible patients were randomized 2:1 to receive either aprepitant triple therapy or the placebo-controlled regimen  B. Attrition bias:  Low risk  Reason: Outcome was assessed for all patients and chemotherapy courses    C. Performance bias  Low risk  Reason: In 4 (intervention group) of the 50 patients, patients and personnel were not blinded. However, in the analysis on vomiting/nausea efficacy these patients were not included. For the rest of the study population both patients and personnel were blinded.  D. Detection bias  Unclear  Reason: Blinding of outcome assessors was unclear |

| **Pharmacological treatment of nausea and vomiting - Midazolam vs Dexamethasone vs Midazolam + dexamethasone vs placebo** | | | | |
| --- | --- | --- | --- | --- |
| ***Riad, W. et al.*** Effect of midazolam, dexamethasone and their combination on the prevention of nausea and vomiting following strabismus repair in children. European Journal of Anaesthesiology 2007; 24: 697-701 | | | | |
| **Study characteristics** | **Patient characteristics** | **Intervention / Control** | **Outcomes / Results** | **Comments**  **Risk of bias** |
| Type of study:  Prospective randomized and double-blind study  Setting:  Single centre, Saudi Arabia  Duration:  Episodes of nausea, and retching and vomiting were recorded during the first 24h after surgery  Study years:  2006/2007, no exact data mentioned  Protocol published in register:  Not reported | Number and type of participants:  Total of 100 children who were scheduled to undergo elective strabismus surgery   - Intervention group 1: 25 children - Intervention group 2: 25 children - Intervention group 3: 25 children - Control group: 25 children   Age:   - Intervention group 1:   Mean/SD: 7.2 (2.4), Range: 4-12 yr.   - Intervention group 2:   Mean/SD: 8.3 (3.6), Range: 4-12 yr.   - Intervention group 3:   Mean/SD: 8.3 (3.9), Range: 4-12 yr.   - Control group: - Mean/SD: 6.7 (2.9), Range: 4-12 yr.   Sex:   - Intervention group 1: M: 15 (60%), F: 10 (40%) - Intervention group 2: M: 12 (48%), F: 13 (52%) - Intervention group 3: M 11 (44%). F: 14 (56%) - Control group: M 14 (56%), F: 11 (44%)   There was no statistically significant difference between groups with regard to age, weight, sex, duration of surgery, n of operated muscles and occurrence of oculocardiac reflex.  Other:  Recovery time in minutes (SD)   - Intervention group 1: 17 minutes (1.7) - Intervention group 2: 24 minutes (1.9) - Intervention group 3: 23 minutes (2.5) - Control group: 15 minutes (2.1)   Recovery time was significantly delayed for intervention group 2 and 3. | Type of intervention:   - Intervention group 1: midazolam 50µgkg^-1^ - Intervention group 2: dexamethasone 0.5mgkg^-1^ (maximum dose, 8mg) - Intervention group 3: combination of midazolam 50µgkg^-1^ and dexamethasone 0.5mgkg^-1^ (maximum dose, 8mg)   Type of control:  Placebo | Outcome definitions:   - Post-operative nausea: subjective feeling that was reported by the patients   Post-operative vomiting: forceful expulsion of liquid or solid gastric contents  Results (per outcome):  *Incidence post-operative nausea*   - Group 1 – midazolam: N = 3 (12%), p < 0.001 compared with placebo - Group 2 – dexamethasone: N=8 (32%), p < 0.01 compared with placebo - Group 3 – Midazolam + dexamethasone N = 0 (0%), p<0.001 compared with placebo - Placebo: N=12 (48%)   *Incidence post-operative vomiting*   - Group 1 – midazolam: N = 0 (0%), p < 0.001 compared with placebo, p < 0.05 compared with dexamethasone - Group 2 – dexamethasone: N = 8 (32%), p < 0.001 compared with placebo - Group 3 – Midazolam + dexamethasone N = 0 (0%), p<0.001 compared with placebo, p < 0.05 compared with dexamethasone - Placebo: N=13 (52%) | Strengths:  Double-blinded, randomized study  Limitations:  Limited information on effect the interventions, 95% confidence intervals not reported. Difference between midazolam and midazolam/dexamethasone is unclear.  **Risk of bias**  A. Selection bias:  Low risk  Reason: patients were randomly divided into one of four groups. Randomization was performed using a table of random numbers and sealed envelopes.  B. Attrition bias:  Low risk  Reason: All patients were followed-up 24 hours after surgery  C. Performance bias  Low risk  Reason: the children and all personnel involved with patient care were unaware of the content of the syringes.  D. Detection bias  Unclear  Reason: unclear if outcome assessors were blinded from knowledge of which intervention was received |

## *Sub-WG 1H Neurological symptoms*

| **Pharmacological treatment for spasticity** | | | | |
| --- | --- | --- | --- | --- |
| ***Olesch CA et al.*** Repeat botulinum toxin-A injections in the upper limb of children with hemiplegia: a randomized controlled trial, Developmental Medicine and Child Neurology, 52, 79-86, 2010 | | | | |
| **Study characteristics** | **Patient characteristics** | **Intervention / Control** | **Outcomes / Results** | **Comments**  **Risk of bias** |
| Type of study:  RCT  Setting:  Single-center, Melbourne,  Australia  Duration:  Outcomes were assessed at baseline, 6 weeks after injection and 16 weeks before the next infection, and after 12 months  Study years:  *June 2001-April 2005*  Protocol published in register: (*clinicaltrials.gov / WHO register*):  not mentioned | Number and type of participants:  *N=22, children with congenital hemiplegic Cerebal Palsy with spasticity affecting upper-limb but no fixed contracture. Aged between 1year 6mths and 5years-old*   - Intervention group: 11 - Control group: 11   Age:  *(mean, median, range)*   - Intervention group:   Mean 3:8 (y:mo) SD 1:0, Range: 1:10 y:mo – 4:10 y:mo   - Control group:   Mean 3:8, SD 0:10, Range: 1:10 y:mo – 4:10 y:mo  Sex:  *(N (%))*   - Intervention group: M: 10 (90.9%), F: 1 (9.1%)90.9% - Control group: M: 9 (81.8%), F: 2(18.2%)   Other:  *At baseline there was a clinically relevant differences in QUEST-scores and spasticity in the fore-arm pronators (p-values not mentioned)* | Type of intervention:  Children received three series of Botulinum Toxin A injections in 16-week cycles in addition to twice-weekly OT. Occupational therapist and physician determined which muscle groups should be targeted. The same muscle groups were targeted each injection cycle. Total dose was dependent on body weight.  Generic OT protocol (not further specified, but available on request) was developed and individualized for each child: twice weekly programme for 6 weeks after injection. First two weeks of intense therapy by study therapist and after this with same intensity by community therapist. Therapists were not blinded.  Therapy based on goal-directed approach. Part of the therapy consisted of home-based activities. The adherence to this home-based program was not recorded.  Type of control:  Same OT program as mentioned above at a comparable time point as the group with injection. | Outcome definitions:  **Primary outcome:**  Parental perception of treatment efficacy (in terms of goal achievement): Assessed by:   - Canadian Occuppational Performance Measure (COPM), semi-structured interview. rating of occupational performance difficulties - Goal Attainment Scale (GAS): and setting of individualized goals.   **Secondary outcomes:**  Level of spasticity: Assessed by an occupational therapist using the Modified Tardieu Scale (MTS). The occupational therapist was blinded for allocation.  Motor performance: Assessed by using the Quality of Upper Extremity Skills Test (QUEST) and Peabody Development Motor Scales -Fine motor (PDMS-FM). The QUEST and PDMS-FM were videotaped and scored later by a blinded rater.  Results (per outcome)  **Parental perception of treatment efficacy:** *COPM performance scores in the intervention group were improved at 12 months.*   - Mean (SD) at 12 months (control vs intervention): 1.7 (0.6) vs 2.5 (1.0) - Difference between groups: -0.8 (95% CI -1.5 to 0.0), p = 0.047   *Satisfaction of COPM not significantly improved in the intervention group at 12 months:*   - Mean (SD) at 12 months (control vs intervention): 1.7 (0.9) vs 2.5 (1.1) - Difference between groups (-0.8 (95%CI -1.7 to 0.1), p = 0.090   *GAS T-scores were improved at 12 months in the intervention group*   - Mean (SD) at 12 months (control vs intervention): 48.8 (9.6) vs 5.8 (6.6) - Difference between groups -6.9 (95% -13.8 to -0.1), p = 0.047.   **Level of spasticity (measured by Modified Tardieu Scale)**  *Level of spasticity at intervention cycle 3 was lower in children treated with BONT-A (intervention group) with regard to:*  *Forearm pronators:*   - Mean (SD) scores at cycle 3 (control vs intervention): 72.2 (28.7) vs 22.7 (3.2 (7.2) - Difference between groups: 50.0 (95%CI 2.4 – 77.6), p = 0.009)   *Wrist flexors:*   - Mean (SD) scores at cycle 3 (control vs intervention): 24.1 (28.5) vs 3.2 (7.2) - Difference between groups: 20.9 (95%CI 2.4 – 39.4), p = 0.029   *Level of spasticity was not significantly lower in children with BONT-A (intervention group) with regard to:*  *Elbow flexors:*   - Mean (SD) scores at cycle 3 (control vs intervention): 77.3 (56.2) 34.5 (48.0) - Difference between groups: 42.7 (95%CI -3.8 – 89.2)), p = 0.070   **Motor performance (measured by QUEST)**  *Quest summary scores and PDMS-FM were not different between the two groups.*  *QUEST*   - Mean (SD) total QUEST Score at cycle 3 (control vs intervention): 72.9 (11.5) vs 79.6 (8.0) - Difference between groups: -6.7 (-15.5 to 17.6), p = 0.833   *PDMS-FM*   - Mean (SD) Score at cycle 3 (control vs intervention): 537.6 (37.2) vs 542.6 (36.2) - Difference between groups: -5.0 (-37.6 to 27.6), p = 0.753   **Adverse events**  Three adverse events were reported: Maculopapular rash (n = 1), weakness of the index finger (n = 1), prolonged weakness in finger flexors (n = 1) | Strengths:  -Single centre study  -Both groups received, although individualized, the same cycle of OT programme  Limitations:  -Too small sample size: They did not reach the sample size needed to detect large of moderate effects.  -OT was partly based om home-based activities of which the adherence was not recorded.  -  **Risk of bias**  A. Selection bias:  low risk  Reason: low risk, allocation sequence remained concealed from the investigator enrolling participants until after the interventions were assigned.  B. Attrition bias:  low risk.  Reason: No patients were lost to follow-up.    C. Performance bias  High risk  Parents and treating OT were not blinded.  D. Detection bias  Unclear  Parents who scored the primary outcomes were not blinded.  The Occupational therapist who scored spasticity and the person who rated motor performance were blinded |

| **Pharmacological treatment for spasticity** | | | | |
| --- | --- | --- | --- | --- |
| ***Copeland I et al.*** Botulinum toxin A for nonambulatory children with cerebral palsy: a double blind randomized controlled trial. J Pediatr 2014; 165:140-6 | | | | |
| **Study characteristics** | **Patient characteristics** | **Intervention / Control** | **Outcomes / Results** | **Comments**  **Risk of bias** |
| Type of study:  RCT, double blind  Setting:  Single centre, Australia  Duration:  Canadian Occupational Performance Measure (COPM) at 4 (immediate effect) and 16 (retention) weeks post intervention.  Study years:  *Not reported*  Protocol published in register: Australia New Zealand Clinical Trials Registry: N12609000360213, PMID 22873758 | Number and type of participants:  *Total of 41 nonambulant children with cerebral palsy at GMFCS levels IV or V, aged 2-16 years, with spasticity in the upper and/or lower limbs causing discomfort and/or increased burden of care Stratification to primary goal areas (upper or lower limb) prior to randomized allocation.*  *Exclusion criteria: weight < 10 kg, medical contraindication to BoNT-A.*   - Intervention group: 23 children - Control group: 18 children   Age:   - Intervention group:   Mean/SD: 7y1m (3y7m), Range NA   - Control group:   Mean/SD: 7y5m (3y9m), Range NA  Sex:   - Intervention group: M: 16 (70%), F: 7 (30%) - Control group: M: 11 (61%), F: 7 (39%)   There were no differences observed between groups on baseline measures regarding GMFCS or MACS level classification, or baseline questionnaire score.  Other:  *Predominant goal area:*  Intervention group: upper limbs 12 (52.2%), lower limbs 11 (47.8%)  Control group: upper limbs 9 (50%), lower limbs 9 (50%) | Type of intervention:  Intramuscular botulinum toxin A (BoNT-A), 0.5-4 units botox/kg/muscle group, maximum dose 12 U botox/kg/body weight (or total 400 units).  Following injections each participant received a block of occupational therapy or physical therapy, which commenced within 2 weeks. Dose of therapy between groups was similar.  Type of control:  Intramuscular sham.  Following sham procedure. Each participant received a block of occupational or physical therapy, within 2 weeks. Treatment regimens were determined prior to randomization based on individual ease of care and comfort goals. Dose of therapy between groups was similar. | Outcome definitions:  **Primary outcomes**  Parental perception of treatment efficacy:  Parent reported change in performance and satisfaction in areas of concern for care and comfort. This was assessed by the Canadian Occupational Performance Measure (COPM)  Positive value indicates improvement of COPM scores for the intervention group in comparison to the control. More than 2 points change is clinically meaningful.  **Secondary outcomes**  For secondary measures of efficacy, the following questionnaires were uses:  CPCHILD - Caregiver Priorities and Child Health Index of Life with Disabilities: Positive value indicates improvement in score  CCHQ - Care and Comfort Hypertonicity Questionnaire: Positive value indicates improvement in score  CPQOL-child - Cerebral Palsy Quality of Life Questionnaire for children: Positive value indicates improvement in score  PPP - Pediatric Pain Profile: Reduction in score indicate improvement in pain  **Adverse events** were measured at 2, 4, 16 weeks.  Results (per outcome):  **Primary outcomes**  *COPM performance*  Estimated mean difference (EMD) between groups (baseline - 4 weeks): 2.2 (95% CI 0.9-3.5; p= .001.  EMD between groups (baseline – 16 weeks): 1.2 (95%CI -0.0 – 2.5); p= NS  Effect was not sustained at 16 weeks.  *COPM satisfaction*  EMD between groups (baseline - 4 weeks): 2.3, (95%CI 0.6-3.9), p= .007.  EMD between groups (baseline – 16 weeks): 1.8 (95% CI 0.2-3.5); p= .03.  **Secondary outcomes**  A significant between groups difference was only observed at 16 weeks for outcome of health status using CPCHILD scores.  *CPCHILD*  EMD between groups (baseline - 4 weeks): 3.7 (95%CI -2.6 – 9.9; p=. NS.  EMD between groups (baseline – 16 weeks): 6.8 (95%CI 1.8 – 11.8); p= .008  *CCHQ*  EMD between groups (baseline - 4 weeks): 3.7 (95%CI -0.9 – 0.2; p=. NS.  EMD between groups (baseline – 16 weeks): -0.3 (95%CI -0.9 – 0.2); p= NS  *CPQOL-Child*  EMD between groups (baseline - 4 weeks): 3.7 (95%CI -0.5 - 8.0); p=. NS.  EMD between groups (baseline – 16 weeks): 2.0 (95%CI -2.9 – 6.8); p= NS  *PPP*  EMD between groups (baseline - 4 weeks): -0.7 (95%CI -15.6 – 14.1)); p=. NS.  EMD between groups (baseline – 16 weeks): 4.5(95%CI -9.5 – 18.5) p= NS  **Adverse events (AE):**  All adverse events (mild, moderate and serious) significantly increased compared with the control group (p = 0.02). When Mild AEs were excluded, no significant difference for moderate and serious AEs were found. | Strengths:  Double-blinded, randomized study  Limitations:  Information on potential difference between previously prescribed oral or intrathecal medication is lacking. Outcomes are parent reported.  Reported EMD and p- values were in abstract and results section are not corresponding.  **Risk of bias**  A. Selection bias:  low risk  Reason: there was random allocation of patients into groups and allocation concealment  B. Attrition bias:  low risk  Reason: no children withdrew from the study. PPP results were reported for 18 children as not all children reported pain at baseline.    C. Performance bias  low risk  Reason: the participants and personnel were blinded from knowledge of which intervention was received  D. Detection bias  low risk  Reason: outcome assessors were blinded from knowledge of which intervention was received |

## *Sub-WG 1I Pain*

| **Non pharmacological treatment of pain** | | | |
| --- | --- | --- | --- |
| ***Eccleston C et al.*** Psychological interventions for parents of children and adolescents with chronic illness. Cochrane Database of Systematic Reviews 2015 4): | | | |
| **Study characteristics** | **Patient characteristics** | **Outcomes / Results** | **Comments**  **Risk of bias** |
| Type of study:  Systematic review of RCTs  Included studies  47 RCTs  Searched databases  CENTRAL, MEDLINE, EMBASE, PsychINFO  Inclusion criteria  *Participants*   - Parents had to be referred to in the title or abstract of each study - The parent had to be the primary caregiver of the child - Children had to have one or more of the chronic illnesses: Asthma, Cancer, Diabetes Mellitus, Gynaecological disorder, inflammatory bowel diseases (IBD), Painful condition (i.e., headache), skin diseases, traumatic brain injury. - Children had to be in the age range: 3 months – 19 yrs. - 10 or more participants in each condition at the end of the treatment assessment.   *Intervention*   - Intervention had to be psychological in at least 1 treatment arm. - design = RCT, - 1 or more parents had to be treated with the intervention - Parents or child had to complete assessments at baseline and at a point in time after/during intervention   *Comparison groups*   - Active treatment group - Treatment-as-usual group - Waiting list control | Number and type of participants:  parents of children with chronic illness such as painful conditions (i.e., including but not exclusively limited to arthritis, back pain, complex regional pain syndrome (CRPS), fibromyalgia, headache, idiopathic pain conditions, irritable bowel syndrome (IBS), recurrent abdominal pain) cancer; diabetes; asthma; traumatic brain injury  Age:  Not reported  Sex:  Not reported  Type of intervention and control  *Intervention:*  Four classes of psychological therapies were tested.   - Cognitive Behavioural Therapy (CBT) – includes a range of strategies with the goals of modifying social/environmental and behavioural factors that may exacerbate or cause symptoms. - Family Therapy (FT) – focus on altering patterns of interactions between family members - Problem-Solving Therapy – didactic instruction in problem-solving, followed by in-session modelling, behavioural rehearsal and performance feedback. - Multi-systemic Therapy – intensive family-community based intervention based on social ecological model and family systems theory. MST targets the child, their family and the school.   *Control:*   - Active treatment group (16 studies) - Treatment-as-usual group (17 studies) - Waiting list control (10 studies) - Three comparator arms (4 studies) | Outcome definitions:  Primary outcomes: 1) Parenting behaviour, 2) Parent mental health  Secondary outcomes: 1) Child behaviour/disability, 2) Child mental health, 3) Child illness-related symptoms, family function and adverse events.  Results (per outcome)  Individual conditions across all psychological therapies.  *Effect of all psychological interventions on parents of children with cancer*.  **Parenting behaviour – post treatment**  Included: 836 (I = 405/C = 431) parents of children from 5 studies  Effect: Psychological had a small beneficial effect for parenting behaviour. SMD is -0.20, 95% CI -0.36 to -0.04, p = 0.01  GRADE level (risk of bias): Very low, Majority of studies have unclear or high risk of bias  **Parenting behaviour – Follow-up**  Included: 789 (I = 399/C=420) parents of children from 5 studies  Effect: Effect was not maintained at follow-up, z = 1.39, p=0.16  GRADE level (risk of bias): Very low, Majority of studies have unclear or high risk of bias  **Parent mental health – post-treatment**  Included: 1010 (I = 494/ C = 516) parents of children from 9 studies  Effect: There was no effect of psychological therapies on parent  mental health post-treatment (Z = 1.86, p = 0.06)  GRADE level (risk of bias): Very low, Majority of studies have unclear or high risk of bias  **Parent mental health – follow-up**  Included: 819 (I = 386, C = 403) parents of children from 6 studies  Effect: Psychological therapies had a small beneficial  effect for improving parent mental health (SMD = -0.18, 95%CI -0.32 to -0.04, Z = 2.58, p = 0.01  GRADE level (risk of bias): Very low, Majority of studies have unclear or high risk of bias  **Child symptoms – post treatment**  Included: 1 study  Effect: no conclusions could be drawn.  Individual psychological therapies across all conditions  *Cognitive behavioural therapy*  **Parenting behaviour – Post treatment**  Included: 166 (I = 86, C = 80) parents of children from 4 studies  Effect: Overall effect of CBT on parenting behaviour was not beneficial (z = 0.08. p = 0.94)  GRADE level (risk of bias)  **Parenting behaviour – follow-up**  Included: 85 (I = 42, C = 43) parents of children from 2 studies  Effect: Overall effect of CBT on parenting behaviour was not beneficial (z = 0.56. p = 0.58)  **Parent mental health – post treatment**  Included: 325 (I = 175, C = 150) parents of children from 7 studies  Effect: No effect of CBT on parent mental health was identified (z = 0.66. p = 0.51)  **Parent mental health – follow-up**  Included: 115 (I = 67, C = 48) parents of children from 2 studies  Effect: No effect of CBT on parent mental health was identified (z = 1.26. p = 0.21)  **Child behaviour/disability – post-treatment**  Included: 487 (I = 247, C = 240) children from 8 studies  Effect: No effect of CBT was identified (z = 1.34. p = 0.18)  **Child behaviour/disability – follow-up**  Included: 289 (I = 150, C = 139) children from 3 studies  Effect: No effect of CBT was identified (z = 0.95. p = 0.34)  **Child mental health – post-treatment**  Included: 439 (I = 232, C = 207) children from 5 studies  Effect: No effect of CBT was identified (z = 0.21 p = 0.83)  **Child mental health – follow-up**  Included: 257 (I = 130, C= 127) children from 2 studies  Effect: No effect of CBT was identified (z = 0.27. p = 0.78)  **Child symptoms – post-treatment**  Included: 754 (I = 396, C = 358) children from 12 studies  Effect: Overall effect of CBT was beneficial (SMD = -0.32, 95%CI -0.53 to -0.11, p <0.01  **Child symptoms– follow-up**  Included: 475 (I = 253, C = 219) children from 7 studies  Effect: No effect of CBT was identified (z = 1.70. p = 0.09)  **Family functioning – post-treatment**  Included: 211 (I = 114, C= 97) children from 3 studies  Effect: No effect of CBT was identified (z = 0.40 p = 0.69)  **Family functioning – follow-up**  Included: 107 (I = 60, C = 47) children from 2 studies  Effect: No effect of CBT was identified (z = 0.61. p = 0.54)  *Family therapy*  **Parent mental health – post treatment**  Included: 131 (I = 74, C = 57) parents of children from 3 studies  Effect: No effect of FT on parent mental health was identified (z = 0.16. p = 0.88)  **Parent mental health – follow-up**  Included: Only 1 study drawn  Effect: No conclusions could be drawn  **Child behaviour/disability – post-treatment**  Included: 107 (I = 53, C = 54) children from 2 studies  Effect: Overall effect of FT was not beneficial for children with chronic condition (z = 1.44. p = 0.15)  **Child symptoms – post-treatment**  Included: 259 (I = 134, C = 125) children from 5 studies  Effect: No beneficial effect was found, SMD -0.32 (-0.53 to -0.11) z = 0.35. p = 0.73) (z = 0.35. p = 0.73)  **Child symptoms– follow-up**  Included: 96(I = 48, C = 48) children from 2 studies  Effect: No beneficial effect was found (z = 0.12. p = 0.91)  **Family functioning**  Included: 132 (I = 63, C = 69) children from 2 studies  Effect: No effect of FT was identified (z = 0.45, p = 0.65)  *Problem solving therapy*  **Parenting behaviour – Post treatment**  Included: 832 (I = 405, C = 427) parents of children from 5 studies  Effect: Small beneficial effect of PST on parenting behaviour (SMD -0.25, 95% CI -0.39 to -0.11, z = 3.59. p <0.01)  **Parenting behaviour – follow-up**  Included: 748 (I = 366, C = 382) parents of children from 4 studies  Effect: Effect was not maintained (z = 0.1.75. p = 0.08)  **Parent mental health – post treatment**  Included: 907 (I = 438, C = 469) parents of children from 7 studies  Effect: Small beneficial effect of PST on parent mental health (SMD -0.24, 95% CI -0.42 to -0.05, z = 2.50. p = 0.01)  **Parent mental health – follow-up**  Included: 778 (I = 379, C = 399) parents of children from 5 studies  Effect: Small beneficial effect of PST on parent mental health (SMD -0.19, 95% CI -0.34 to -0.04, z = 2.55. p = 0.01)  **Child behaviour/disability – post-treatment**  Included: 260 (I = 130, C= 130) children from 5 studies  Effect: No effect of PST was identified (z = 1.21. p = 0.22)  **Child behaviour/disability – follow-up**  Included: only 1 study included  Effect: No conclusions could be drawn  **Child symptoms – post-treatment**  Included: 216 (I = 105, C = 111) children from 2 studies  Effect: No beneficial effect of PST (z = 1.41, p = 0.59)  **Child symptoms– follow-up**  Included: only 1 study included  Effect: No conclusions could be drawn  **Family functioning – post-treatment**  Included: 183 (I = 90, C = 93) children from 3 studies  Effect: No effect of PST was identified (z = 0.54 p = 0.59)  *Multisystem therapy*  **Child behaviour/disability – post-treatment**  Included: 313 I = 158, C = 155) children from 2studies  Effect: No effect of MST was found at reducing child behaviour/disability (z = 0.99, p = 0.32)  **Child behaviour/disability – follow-up**  Included: only 1 study included  Effect: No conclusions could be drawn  **Child mental health– post-treatment**  Included: only 1 study included  Effect: No conclusions could be drawn  **Child mental health– follow-up**  Included: only 1 study included  Effect: No conclusions could be drawn  **Child symptoms – post-treatment**  Included: 455 (I = 230, C = 225) children from 4 studies  Effect: No beneficial effect of MST (z = 1.52, p = 0.13)  **Child symptoms– follow-up**  Included: 247(I = 123, C= 124) children from 2 studies  Effect: No beneficial effect of MST (z = 1.47, p = 0.14) | Strengths:  Large number of studies included  Outcomes are assessed per condition and per psychological therapy  Limitations:  Definitions of primary and secondary outcomes are not reported  **Risk of bias**  Selection bias:  Low risk: 24/47 studies  High risk: 0/47 studies  Unclear: 23/47 studies  Detection bias:  Low risk: 20/47 studies  High risk: 27/47 studies  Unclear: 0/47 studies  Attrition bias:  Low risk: 15/47 studies  High risk: 10/47 studies  Unclear: 23/47 studies  Reporting bias:  Low risk: 18/47 studies  High risk: 15/47 studies  Unclear:14/47 studies  CBT – child symptoms |

| **Pharmacological treatment of pain** | | | |
| --- | --- | --- | --- |
| ***Beecham E et al.*** Pharmacological interventions for pain in children and adolescents with life-limiting conditions. Cochrane Database of Systematic Reviews 2015 3(13) | | | |
| **Study characteristics** | **Population** | **Main outcomes / Results** | **Conclusions**  **Risk of bias** |
| Type of study:  Systematic review of RCTs  Included studies  9 studies (10 articles)  Searched databases  CENTRAL, MEDLINE, EMBASE, PsycINFO, CINAHL  Selection criteria  Inclusion:   - randomised controlled trials (RCTs) (including cluster RCTs and cross-over trials), quasi-randomised studies, n of 1 study, studies that are not randomised but include a clearly defined comparator group, and time series analyses that have investigated pharmacological treatments for pain associated with LLC in Children or Young people   Exclusion:   - not relevant topic area, Adults only, not life limiting, no pain outcomes) | Number and type of participants:  379 children and young people with life-limiting conditions (LLC)  Age:  Range: 0 – 18 years (see result section for specific range per treatment group)  Sex:  *(N (%)) unknown*  Type of intervention and control  *Intervention:*  Pharmacological intervention given at any dose for any time period. Pharmacological intervention could be developed specifically to treat pain and could act as an adjuvant meaning that treatment was not primarly developed to treat pain but has pain relieving properties.  *Control:*  Other pharmacological interventions, psychological interventions, placebo, alternative dosing regimens or routes of administration | Main outcomes  **Primary outcomes**   - Pain control: measured by changes in pain intensity scales or changes in physiological parameters - Safety: Adverse events   **Secondary outcomes**   - Changes in physical and psychological functioning and well-being measured by scales assessing quality of life and well-being quality of care.   Results  ***Patients with cerebral palsy***  *Intrathecal baclofen vs placebo or normal therapy*  *Total participants*: 21 children with CP aged 7 to 17   - N = 4 (Bonouvrie 2011) - N = 17 (Hoving, 2007; Hoving 2009)   *Intervention vs control*   - Intrathecal baclofen vs placebo (Bonouvrie, 2011) - Intrathecal baclofen vs therapy as normal (Hoving, 2007   *Pain outcomes*   - Pain measured using Visual Analogue Scale (0-10): Significant decrease of pain after administration of intrathecal baclofen in the intervention group compared to standard therapy in the control group. Mean Difference: 4.20, 95%CI 2.1 to 6.25 (Hoving, 2009) - Pain measured using VAS (0-10) at 6-month follow-up: Significant decrease of pain in the intervention group as compared to placebo. Mean difference: 4.20, 95%CI 2.15 to 6.25 (Hoving, 2007) - Bodily pain or discomfort measured using Child Health Questionnaire-parent form at 6-months follow-up: Decrease of pain in the intervention group. Mean difference 26.60, 95%CI 2.61 to 50.59 (Hoving, 2007).   Pain measured using VAS: Decrease of pain with 2.6 points in the intervention groups. Pain scores increased in the placebo group (Bonouvrie, 2011)  *Safety outcomes*  Number and type of adverse effects   - Nine adverse effects in 8 of 17 participant, mostly related to Cerebrospinal Fluid (CSF leakage) (Hoving, 2007) - Fourteen of 17 patients experienced a total of 28 procedure or device related adverse events, mostly related to swelling at pump site (Hoving, 2009) - 2 of 4 patients experienced CSF leakage which in discontinuation of trial in one patient (Bonouvrie, 2011)   Most common adverse effect   - Most common adverse effect irrespective of treatment arm was related to CSF leakage, respectively 2 patients (Bonouvrie, 2011) and 3 patients (Hoving, 2007).   *Botulinum toxin A or Botulinum toxin A and occupational therapy vs placebo or occupational therapy alone*  *Total participants*: 84 children with CP aged 2 to 16   - N = 41 (Copeland, 2014) - N = 43 (Russo, 2007)   *Intervention vs control*   - Botulinum Toxin A vs. placebo (Copeland, 2014) - Botulinum Toxin A with Occupational Therapy (OT) vs. OT only (Russo, 2007)   *Pain outcomes*   - Pain measured using the Pediatric Pain Profile at 1 month follow-up: No significant difference in pain scores between intervention and control group. Mean Difference -2.67, 95% CI -10.18 to 4.84 (Copeland, 2014) - Pain measured using the Pediatric Pain Profile at 4-month follow-up: No significant difference in pain scores between intervention and control group. Mean Difference 2.59, 95% CI -3.75 to 8.93 (Copeland, 2014) - Pain measured using VAS at 3-month follow-up (2 participants in each group): No significant difference in pain scores between intervention and control group. OR 1.05, 95%CI 0.13 to 8.24 (Russo, 2007) - Pain measured using VAS at 6-month follow-up (1 participants in each group): No significant difference in pain scores between intervention and control group. OR 1.05, 95% CI 0.06to 17.95 (Russo, 2007)   *Safety outcomes*  Number and type of participants with adverse events (intervention vs control)   - 1 participant with epilepsy and hospital admission vs 2 participants with hospital admission due to epilepsy (Russo, 2007) - 3 participants with systemic drooling, decreased vocalization or drooling vs 1 participant (Copeland, 2014)   Number and type of adverse effects (intervention vs control)   - 22 adverse effects (feeling unwell) vs 0 adverse effects (Russo, 2007) - 23 patients with moderate or mild adverse effects (Copeland, 2014)   Most common adverse effect  Most common reported effect were seizures and respiratory symptoms  ***Patients with Osteogenesis imperfecta***  *Oral alendronate vs placebo*  *Total participants*: 159 children with OI aged 3 to 19   - N = 20 (Seikaly, 2005) - N = 139 (Ward, 2011)   *Intervention vs control*   - Oral alendronate vs placebo   *Pain outcomes*   - Pain measured by number of pain-free days per month at 12-month follow-up: Significant decrease of pain in the intervention group. Mean difference, MD-3.63, 95%CI -5.17 to -2.09 (Seikaly, 2005) - Pain measured by number of days with analgesic use for skeletal pains at 12-month follow up: Significant decrease of analgesic use in the intervention group. Mean Difference, -2.00, 95% CI -3.57 to -0.43 (Seikaly, 2005) - Pain measured by number of patients with bone pain at 24-month follow-up: In the intervention group fewer patients experienced pain in comparison to placebo (37%, 38/102 vs. 57%, 17/30). This effect was not statistically significant. OR, 0.45, 95% CI 0.20 to 1.04 (Ward, 2011) - Pain measured by number of days per week that patients experienced bone pain at 24-month follow-up: No significant difference the intervention group at baseline and follow-up (Ward, 2011).   *Safety outcomes*  Number and type of participants with adverse events (intervention vs control)   - 2 participants vs 1 participant. This resulted in withdrawal from the study (Ward, 2011)   Number and type of adverse effects   - 2 of 20 participants with abdominal discomfort (Seikaly, 2005) - 50% of 139 participants experienced gastrointestinal symptoms. No difference in treatment arm (Ward, 2011)   Most common adverse effect  Most common reported effects were gastrointestinal symptoms.  *Oral risedronate vs placebo*  *Total participants*: unknown (bishop, 2013)  *Intervention vs control:* Oral risedronate vs placebo  *Pain outcomes*  Pain was considered an adverse event and was measured using pain scales: When pain was reported as an adverse event there was no significant difference between the intervention of control group in the number of participants experiencing pain. OR 1.54,95% CI 0.52 to 4.56 (Bishop, 2013). No difference in pain scales was measured (discussion of Bishop, 2013)  *Safety outcomes*  Number of participants with adverse events (intervention vs control)  No significant difference in number of adverse events between intervention and control group. OR 0.46, 95% CI 0.09 to 2.24 (Bishop, 2013)  *Intravenous pamidronate vs no treatment*  *Total participants*: Total participants 18 (Letocha, 2013)  *Intervention vs control:* Intravenous pamidronate vs placebo  *Pain outcomes*  Pain measured by a 4-point self-reported pain scale (from 4 = no pain to 1 = intractable pain): No differences in self-reported bone pain were found. Mean difference: -0.11, 95% CI -0.83 to 0.61 (Letocha, 2005)  *Safety outcomes*  All participants experienced acute phase reactions upon the first infusion cycle of pamidronate. What these reactions were are not described; no other complications were noted (Letocha, 2005) | **Conclusions**  unable to determine the effects of pharmacological interventions for pain for  CYP with LLCs.  **Additional remarks**  Strengths: --  Limitations:  The National Institute for Health Research (NIHR)  **Risk of bias**  *Intrathecal baclofen vs placebo/normal therapy*  *Selection bias:* Low in 2/3 and unclear in 1/3  *Attrition bias*: low in 1/3, unclear in 1/3 and high in 1/3.  *Performance bias:* low in 1/3, unclear in 1/3 and high in 1/3.  *Detection bias:* low in 1/3, unclear in 1/3 and high in 1/3  *Botulinum toxin A or Botulinum toxin A and occupational therapy vs placebo or OT only*  Selection bias: Low in 1/2 and unclear in 1/2  Attrition bias low in 2/2  Performance bias: low in 1/2 and high in 1/2; Detection bias: low in 1/2 and high in 1/2.  *Oral alendronate vs placebo*  Selection bias: Low in 1/2 and unclear in 1/2; Attrition bias: high in 1/2 and unclear in 1/2; Performance bias: low in 2/2.  Detection bias: low in 1/2 and unclear in 1/2.  *Oral risedronate vs placebo*  Selection bias: Low; Attrition bias: low; Performance bias: low; Detection bias: low  *Intravenous pamidronate vs no treatment*  election bias: Unclear; Attrition bias: low; Performance bias high; Detection bias: high |

| **Pharmacological treatment of pain** | | | |
| --- | --- | --- | --- |
| ***Wiffen PJ et al.*** Opioids for cancer‐related pain in children and adolescents. Cochrane Database of Systematic Reviews 2017 7): The Journal of Clinical Endocrinology and Metabolism 2011;96(2):355–64. | | | |
| **Study characteristics** | **Population** | **Outcome definitions / Main results** | **Conclusions**  **Risk of bias** |
| Type of study:  Systematic review of RCTs  Included studies  0  Searched databases  Cochrane Central Register of Controlled Trials (CENTRAL); MEDLINE (via Ovid); Embase (via Ovid)  Selection criteria  Inclusion criteria:   - Type of studies: Randomised controlled trials (RCTs), with or without blinding, and participant or observer reported outcomes. - Type of participants: infants, children, and adolescents aged from birth to 17 years, who have (one or more) cancer and experience pain directly related to the condition. - Type of interventions: studies reporting interventions prescribing any opioid drug (alone or in combination) for the relief of cancer pain; by any route, in any dose, with comparison to a placebo or any active comparator. - Type of outcome measure: studies reporting pain assessments. For example, measuring pain intensity and pain - relief assessed using validated tools such as numerical rating scale (NRS), visual analogue scale (VAS), Faces Pain Scale – Revised (FPS-R), Colour Analogue Scale (CAS), or any other validated rating scale.   Exclusion criteria:  studies of perioperative pain, short-term infection pain, short-term injury or trauma pain, acute pain, functional abdominal pain, burn pain, and musculoskeletal pains, headache and migraine, sickle cell disease acute crisis pain, mucositis, or any other chronic non-cancer related pain. | Number and type of participants:  0  Age:  Not applicable  Sex:  Not applicable  Other:  Not applicable | Outcome definitions   - Participant-reported pain relief of 30% or greater. - Participant-reported pain relief of 50% or greater. - GIC much or very much improved   Main results  There were no randomised controlled trials (RCTs) identified for inclusion. | **Conclusions**  We identified no randomised controlled trials (RCTs), to support or refute the use of opioids to treat cancer pain in children and adolescents.  **Additional remarks**  Strengths: --  Limitations:  National Institute for Health Research (NIHR), UK.  **Risk of bias**  Not applicable |

# Appendix K. Summary of findings tables, appraisal of evidence, and conclusions of evidence

## *Sub-WG 1C Dyspnoea*

### Non invasive ventilation

| **Non-invasive ventilation** | | | | | |
| --- | --- | --- | --- | --- | --- |
| Studies | Type of participants | | Total no. of participants (intervention vs control) | Type of intervention vs control | Outcome and Effect size |
| **Degree of dyspnoea,** measured by modified Borg Scale or Medical Research Council (MRC) Dyspnoea Scale, higher score indicating higher degree of dyspnoea | | | | | |
| 1) Lima, 2014 | 1) children and young adolescents with CF aged 7-15 years | | 1) 13 (13 vs 13)  Open randomized controlled cross-over trial. Participants acted as their own control. | 1) 6-min Treadmill Walking Test (TWT) with non-invasive Ventilation vs 6-min Treadmill Walking test without non-invasive ventilation | **1) Modified Borg Scale score**  No significant difference in scores between intervention and control group was found. |
| **Grade assessment** | | | | | |
| Study design: | +4 | 1 Randomized Controlled Trial | | | |
| Study limitations | -1 | Some limitations - Selection bias: Low; Attrition: bias low; Performance bias: high; Detection bias: unclear | | | |
| Consistency: | 0 | No important inconsistency. Only 1 study performed. | | | |
| Directness: | 0 | Results are direct. Outcomes generalizable. | | | |
| Precision: | -2 | Some imprecisions due to small sample size. Only 1 study performed | | | |
| Publication bias: | 0 | Unlikely | | | |
| Effect size: | 0 | No large magnitude of effect | | | |
| Dose-response: | 0 | Unclear dose-response relationship. | | | |
| Plausible confounding: | 0 | No plausible confounding | | | |
| **Quality of evidence:** |  | **⊕⊖⊖⊖ VERY LOW** | | | |
| **Conclusion:** |  | **There is very low quality of evidence there is no significant effect of walking with non-invasive ventilation in children with Cystic Fibrosis on degree of dyspnoea as compared to walking without non-invasive ventilation.** | | | |

| **Non-invasive ventilation** | | | | | |
| --- | --- | --- | --- | --- | --- |
| Studies | Type of participants | | Total no. of participants (intervention vs control) | Type of intervention vs control | Outcome and Effect size |
| **Exercise capacity** | | | | | |
| 1) Lima, 2014 | 1) children and young adolescents with CF aged 7-15 years | | 1) 13 (13 vs 13)  Open randomized controlled cross-over trial. Participants acted as their own control. | 1) 6-min Treadmill Walking Test (TWT) with non-invasive Ventilation vs 6-min Treadmill Walking test without non-invasive ventilation | **Walking distance (intervention vs control)**  Mean (SD) 415.38m (77.52) vs 386.92m (84.89), p = 0.039.  **Exercise capacity (cardiorespiratory variables)**   - Peripheral oxygen saturation (SpO_2_): No significant difference between groups - Heart Rate (HR): No significant difference between groups - Respiratory rate (RR): No significant difference between groups |
| **Grade assessment** | | | | | |
| Study design: | +4 | 1 Randomized Controlled Trial | | | |
| Study limitations | -1 | Some limitations - Selection bias: Low; Attrition: bias low; Performance bias: high; Detection bias: unclear | | | |
| Consistency: | 0 | No important inconsistency. Only 1 study performed. | | | |
| Directness: | 0 | Results are direct. Outcomes generalizable. | | | |
| Precision: | -2 | Some imprecisions due to small sample size. Only 1 study performed | | | |
| Publication bias: | 0 | Unlikely | | | |
| Effect size: | 0 | No large magnitude of effect | | | |
| Dose-response: | 0 | Unclear dose-response relationship. | | | |
| Plausible confounding: | 0 | No plausible confounding | | | |
| **Quality of evidence:** |  | **⊕⊖⊖⊖ VERY LOW** | | | |
| **Conclusion:** |  | **There is very low quality of evidence that walking with non-invasive ventilation during 6-min TWT in children with Cystic Fibrosis increases exercise capacity (walking distance) as compared to walking without non-invasive ventilation (no significant effect on peripheral oxygen saturation, heart rate, respiratory rate)** | | | |

| **Non-invasive ventilation** | | | | | |
| --- | --- | --- | --- | --- | --- |
| Studies | Type of participants | | Total no. of participants (intervention vs control) | Type of intervention vs control | Outcome and Effect size |
| **Pulmonary function** | | | | | |
| 1) Lima, 2014 | 1) children and young adolescents with CF aged 7-15 years | | 1) 13 (13 vs 13)  Open randomized controlled cross-over trial. Participants acted as their own control. | 1) 6-min Treadmill Walking Test (TWT) with non-invasive Ventilation vs 6-min Treadmill Walking test without non-invasive ventilation | **Pulmonary function variables**   - Forced expiratory volume in the first second (FEV1 in ml):   Significant increase after TWT in the intervention group, p = 0.036   - Minute Volume (MV in L):   Significant increase after TWT in the intervention group, p=0.013   - Tidal volume (Vt in L):   Significant increase after TWT in the intervention group, p=0.005   - Pulmonary rib cage volume (Vrcp in %):   Significant increase after TWT in the intervention group, p = 0.011   - Forced expiratory volume in the first second (FEV1 in %); Forced vital capacity (FVC in l and %); forced expiratory flow of FVC (FEF 25-75 in ml/s); abdominal rib cage volume (Vrca in %); abdominal volume (Vab in %); inspiratory time (Ti in s); expiratory time (Te in s) Total ventilatory cycle time (Ttot in s); duty cycle (Ttot/Ti in %)   No significant difference before and after TWT in both intervention and control group |
| **Grade assessment** | | | | | |
| Study design: | +4 | 1 Randomized Controlled Trial | | | |
| Study limitations | -1 | Some limitations - Selection bias: Low; Attrition: bias low; Performance bias: high; Detection bias: unclear | | | |
| Consistency: | 0 | No important inconsistency. Only 1 study performed. | | | |
| Directness: | 0 | Results are direct. Outcomes generalizable. | | | |
| Precision: | -2 | Some imprecisions due to small sample size. Only 1 study performed | | | |
| Publication bias: | 0 | Unlikely | | | |
| Effect size: | 0 | No large magnitude of effect | | | |
| Dose-response: | 0 | Unclear dose-response relationship. | | | |
| Plausible confounding: | 0 | No plausible confounding | | | |
| **Quality of evidence:** |  | **⊕⊖⊖⊖ VERY LOW** | | | |
| **Conclusion:** |  | **There is very low quality of evidence that walking with non-invasive ventilation in children with Cystic Fibrosis increases pulmonary function (forced expiratory volume in the first second, minute volume, tidal volume and pulmonary ribcage volume) as compared to walking without non-invasive ventilation (no significant effect on FEV1 %, FVC in ml and %, FEF25e75, Vrca, Vab, Ti, Te, Ttot, Ti/tot, RR/vT^1^).** | | | |

^1^FEV1, forced expiratory volume in the first second; FVC, forced vital capacity; FEF 25e75, forced expiratory flow of 25%e75% of FVC; Vrca, abdominal rib cage volume; Vab, abdominal volume; Ti, inspiratory time; Te, expiratory time; Ttot, total ventilatory cycle time; Ti/Ttot, duty cycle; RR/vt, Frequency/tidal volume ratio

### High intensity training

| **high intensity training** | | | | | |
| --- | --- | --- | --- | --- | --- |
| Studies | Type of participants | | Total no. of participants (intervention vs control) | Type of intervention vs control | Outcome and Effect size |
| **Degree of dyspnoea,** measured by modified Borg Scale or Medical Research Council (MRC) Dyspnoea Scale, higher score indicating higher degree of dyspnoea | | | | | |
| 1) de Jong, 2001^1^ | 1) children with CF aged 10-25 years   - Intervention: Mean (SD): 19 (5.5) years - Control: Mean (SD): 17 (5.2) years | | 1) 16 (8 vs 8) | 1) High intensity training, trained up to 40% maximal static inspiratory pressure for 6 weeks vs low intensity training, trained up to 10% maximal static inspiratory pressure for 6 weeks | **1) Change in degree of dyspnoea from baseline to post-treatment (intervention vs control)**   - Borg _max_, endurance (score at maximal work load during inspiratory muscle endurance test):   Mean (SD)_Post-treatment – baseline_ 1.3 (1.3) -1.4 (1.3) vs 1.0 (1.8) – 1.0 (1.8), p = 0.603   - Borg _max_, bicycle: Borg (score at maximal work load during bicycle test)   Mean (SD)_Post-treatment –baseline_ 4.3 (3.5)- 5.3(2.7) vs 4.5 (3.3) - 4.2 (3.3), p = 0.603   - MRC Dyspnoea scale:   Mean (SD)_Post-treatment – baseline_ 0,33 (0.82) – 0.43 (0.79) vs 0.50 (0.76) – 0.63 (1.06), p = 0.351 |
| **Grade assessment** | | | | | |
| Study design: | +4 | 1 Randomized Controlled Trial | | | |
| Study limitations | -2 | Serious limitations - Selection bias: unclear; Attrition bias: high; Performance bias: unclear; Detection bias: unclear | | | |
| Consistency: | 0 | No important inconsistency. Only 1 study performed. | | | |
| Directness: | 0 | Results are direct. Outcomes generalizable. | | | |
| Precision: | -2 | Some imprecisions due to small sample size. Only 1 study performed. | | | |
| Publication bias: | 0 | Unlikely | | | |
| Effect size: | 0 | No large magnitude of effect | | | |
| Dose-response: | 0 | Unclear dose-response relationship. | | | |
| Plausible confounding: | 0 | No plausible confounding | | | |
| **Quality of evidence:** |  | **⊕⊖⊖⊖ VERY LOW** | | | |
| **Conclusion:** |  | **There is very low quality of evidence there is no significant effect of high intensity training in children with Cystic Fibrosis on degree of dyspnoea as compared to low intensity training.** | | | |

^1^ Selection bias unclear: Patients are randomized, allocation concealment was not reported. Attrition bias = high, 12.5% (n = 1) loss to follow-up in both study arms, performance bias: unclear if researchers and participants were blinded from allocation to study arm. Detection bias: unclear, blinding of outcome assessors was not

| **High intensity training** | | | | | |
| --- | --- | --- | --- | --- | --- |
| Studies | Type of participants | | Total no. of participants (intervention vs control) | Type of intervention vs control | Outcome and Effect size |
| **Exercise capacity** | | | | | |
| 1) de Jong, 2001^1^ | 1) children with CF aged 10-25 years   - Intervention: Mean, 19 (5.5) years - Control: Mean, 17 (5.2) years | | 1) 16 (8 vs 8) | 1) High intensity training, trained up to 40% maximal static inspiratory pressure for 6 weeks vs low intensity training, trained up to 10% maximal static inspiratory pressure for 6 weeks | **Change in exercise capacity from baseline to post-treatment (intervention vs control)**   - Maximal Exercise capacity (Wmax in W):   No significant difference between groups, p = 0.166   - Maximal volume uptake (VO_2_ Max in ml kg^-1^min^-1^):   No significant difference between groups, p = 0.995   - Maximum ventilation (VEmax in L/min):   No significant difference between groups, p = 0.347   - Maximal static inspiratory pressure (Pimax in % pred.):   No significant difference between groups, p = 0.401   - Inspiratory muscle endurance (IME in %Pimax):   Significant increase of IME (%PIMAX) in the intervention group  Mean (SD)_Post-treatment – baseline_: 66 (14) - 49 (12) vs 54 (7) – 50 (5), p = 0.012 |
| **Grade assessment** | | | | | |
| Study design: | +4 | 1 Randomized Controlled Trial | | | |
| Study limitations | -2 | Serious limitations - Selection bias: unclear; Attrition bias: high; Performance bias: unclear; Detection bias: unclear | | | |
| Consistency: | 0 | No important inconsistency. Only 1 study performed. | | | |
| Directness: | 0 | Results are direct. Outcomes generalizable. | | | |
| Precision: | -2 | Some imprecisions due to small sample size. Only 1 study performed. | | | |
| Publication bias: | 0 | Unlikely | | | |
| Effect size: | 0 | No large magnitude of effect | | | |
| Dose-response: | 0 | Unclear dose-response relationship. | | | |
| Plausible confounding: | 0 | No plausible confounding | | | |
| **Quality of evidence:** |  | **⊕⊖⊖⊖ VERY LOW** | | | |
| **Conclusion:** |  | **There is very low quality of evidence that high intensity training in children with Cystic Fibrosis increases exercise capacity (inspiratory muscle endurance) as compared to low intensity training (no significant effect on Wmax, VO_2_max, VEmax, PiMax)^2^.** | | | |

^1^ Selection bias unclear: Patients are randomized, allocation concealment was not reported. Attrition bias = high, 12.5% (n = 1) loss to follow-up in both study arms, performance bias: unclear if researchers and participants were blinded from allocation to study arm. Detection bias: unclear, blinding of outcome assessors was not

^2^ Wmax, Maximal exercise capacity; VO_2_max, maximal volume uptake; VEmax, Maximum ventilation; Pimax, Maximum static inspiratory pressure

| **High intensity training** | | | | | |
| --- | --- | --- | --- | --- | --- |
| Studies | Type of participants | | Total no. of participants (intervention vs control) | Type of intervention vs control | Outcome and Effect size |
| **Pulmonary function** | | | | | |
| 2) de Jong, 2001^1^ | 2) children with CF aged 10-25 years   - Intervention: Mean (SD): 19 (5.5) years   Control: Mean (SD): 17 (5.2) years | | 2) 16 (8 vs 8) | 2) High intensity training, trained up to 40% maximal static inspiratory pressure for 6 weeks vs low intensity training, trained up to 10% maximal static inspiratory pressure for 6 weeks | **Change in pulmonary function from baseline to post-treatment (intervention vs control)**   - Forced expiratory volume in the first second (FEV1 in L):   No significant difference between groups, p = 0.822   - Forced expiratory volume in the first second (FEV1 % pred.):   No significant difference between groups, p = 0.460   - Forced vital capacity (FVC in l):   No significant difference in between groups, p = 0.999   - Forced vital capacity (FVC in % pred.):   No significant difference between groups, p = 0.789 |
| **Grade assessment** | | | | | |
| Study design: | +4 | 1 Randomized Controlled Trial | | | |
| Study limitations | -2 | Serious limitations - Selection bias: unclear; Attrition bias: high; Performance bias: unclear; Detection bias: unclear | | | |
| Consistency: | 0 | No important inconsistency. Only 1 study performed. | | | |
| Directness: | 0 | Results are direct. Outcomes generalizable. | | | |
| Precision: | -2 | Some imprecisions due to small sample size. Only 1 study performed. | | | |
| Publication bias: | 0 | Unlikely | | | |
| Effect size: | 0 | No large magnitude of effect | | | |
| Dose-response: | 0 | Unclear dose-response relationship. | | | |
| Plausible confounding: | 0 | No plausible confounding | | | |
| **Quality of evidence:** |  | **⊕⊖⊖⊖ VERY LOW** | | | |
| **Conclusion:** |  | **There is very low quality of evidence there is no significant effect of high intensity training in children with Cystic Fibrosis on pulmonary function (FEV1 in L and %, FVC in L and %) as compared to low intensity training** | | | |

^1^ Selection bias unclear: Patients are randomized, allocation concealment was not reported. Attrition bias = high, 12.5% (n = 1) loss to follow-up in both study arms, performance bias: unclear if researchers and participants were blinded from allocation to study arm. Detection bias: unclear, blinding of outcome assessors was not reported.

## *Sub-WG 1D Haematological symptoms*

### Erytropoetin (Epoetin Alfa)

| **Epoetin Alfa** | | | | | |
| --- | --- | --- | --- | --- | --- |
| Studies | Type of participants | | Total no. of participants (intervention vs control) | Type of intervention vs control | Outcome and Effect size |
| **Mean haemoglobin levels,** g/dL | | | | | |
| 1) Buyukpamukcu, 2002 | 1) Children with cancer- or chemotherapy-related anaemia aged 1 to 16 yrs. | | 1) 34 (17 vs 17) | 1) Epoetin Alfa dose of 150units /kg, administered 3 times per week for 8 weeks vs no intervention | 1) **Mean Haemoglobin level (g/dL) – intervention vs control**  *Study entry:* 8.5 g/dL vs 8.48 g/dL, P = NS  *Study end:* 10.21 g/dL vs 8.41 g/dL, p = 0.027  *Over the course of the study (from study entry to study end):*  Intervention group*:* 8.50 to 10.21 g/dL, p = 0.086  Control group: 8.48 to 8.41 g/dL, p = NS |
| 2) Razouk, 2006 | 2) Anaemic paediatric patients who received myelosupressive chemotherapy for nonmyeloid malignancies aged 5 to 18 yrs. | | 2) 222 (111 vs 111) | 2) Epoetin Alfa dose of 600units/kg to 900units/kg (if Hb had not increased by 1g/dL or more from baseline), administered intravenously 1 time per week for 16 months vs placebo administered intravenously 1 time per week for 16 months | 2) **Mean (SD) change in Haemoglobin level (g/dL) – intervention vs control**  1.3 (2.38) vs 1.0 (1.90); EMD _intervention - control_ = 0.37 (95%CI -0.11 to 0.84), p=0.129 |
| **Grade assessment** | | | | | |
| Study design: | +4 | 2 Randomized Controlled Trials | | | |
| Study limitations | -2 | Serious limitations - Selection bias: Unclear in 2/2; Attrition bias: Low in 1/2 and unclear in 1/2; Performance bias: unclear in 2/2; Detection bias: unclear in 2/2 | | | |
| Consistency: | 0 | No important inconsistency. All studies show that haemoglobin levels are higher in children receiving Epoetin Alfa | | | |
| Directness: | 0 | Results are direct. Outcomes are generalizable. | | | |
| Precision: | 0 | No important imprecision, large sample size | | | |
| Publication bias: | 0 | Unlikely | | | |
| Effect size: | 0 | No large magnitude of effect | | | |
| Dose-response: | 0 | Unclear dose-response relationship | | | |
| Plausible confounding: | 0 | No plausible confounding | | | |
| **Quality of evidence:** |  | **⊕⊕⊖⊖ LOW** | | | |
| **Conclusion:** |  | **There is low quality of evidence that there is no significant effect of Epoetin Alfa (dose starting from 450units/kg per week) on haemoglobin levels of children with cancer- or chemotherapy related anaemia as compared to no treatment or placebo. However, in one study haemoglobin levels did increase in the intervention group (no significant effect).** | | | |

| **Epoetin Alfa** | | | | | |
| --- | --- | --- | --- | --- | --- |
| Studies | Type of participants | | Total no. of participants (intervention vs control) | Type of intervention vs control | Outcome and Effect size |
| **Red Blood Cell Transfusion,** Number of patients that required Red Blood Cell transfusions | | | | | |
| 1) Buyukpamukcu, 2002 | 1) Children with cancer- or chemotherapy-related anaemia aged 1 to 16 yrs. | | 1) 34 (17 vs 17) | 1) Epoetin Alfa dose of 15units /kg, administered 3 times per week for 8 weeks vs no intervention | 1) **N (%) of patients with Red Blood Cell Transfusion - intervention vs control**  1 (5.9%) vs 8 (47%), p = 0.08 |
| 2) Razouk, 2006 | 2) Anaemic paediatric patients who received myelosupressive chemotherapy for nonmyeloid malignancies aged 5 to 18 yrs. | | 2) 222 (111 vs 111) | 2) Epoetin Alfa dose of 600units/kg to 900units/kg (if Hb had not increased by 1g/dL or more from baseline), administered intravenously 1 time per week for 16 months vs placebo administered intravenously 1 time per week for 16 months | 2)  **N (%) of patients with Red Blood Cell Transfusion - intervention vs control**  *Over the course of the study:* 72 (64.9%) vs 86 (77.5%)  *After week 4:* 38.7% (intervention) vs 22.5% (control), p = 0.10  (patients were more likely to remain transfusion free) |
| **Grade assessment** | | | | | |
| Study design: | +4 | 2 Randomized Controlled Trials | | | |
| Study limitations | -2 | Serious limitations - Selection bias: Unclear in 2/2; Attrition bias: Low in 1/2 and unclear in 1/2; Performance bias: unclear in 2/2; Detection bias: unclear in 2/2 | | | |
| Consistency: | 0 | No important inconsistency. All studies show that number of patients who required blood transfusions is lower in the Epoetin Alfa group | | | |
| Directness: | 0 | Results are direct. Outcomes are generalizable. | | | |
| Precision: | 0 | No important imprecision, large sample size | | | |
| Publication bias: | 0 | Unlikely | | | |
| Effect size: | 0 | No large magnitude of effect | | | |
| Dose-response: | 0 | Unclear dose-response relationship | | | |
| Plausible confounding: | 0 | No plausible confounding | | | |
| **Quality of evidence:** |  | **⊕⊕⊖⊖ LOW** | | | |
| **Conclusion:** |  | **There is low quality of evidence that there is no significant effect of Epoetin Alfa (dose starting from 450units/kg per week) on the number of required blood cell transfusions in children with cancer- or chemotherapy-related anaemia as compared to no treatment or placebo.** | | | |

| **Epoetin Alfa** | | | | | |
| --- | --- | --- | --- | --- | --- |
| Studies | Type of participants | | Total no. of participants (intervention vs control) | Type of intervention vs control | Outcome and Effect size |
| **Safety,** adverse effect and adverse events | | | | | |
| 1) Buyukpamukcu, 2002 | 1) Children with cancer- or chemotherapy-related anaemia aged 1 to 16 yrs. | | 1) 34 (17 vs 17) | 1) Epoetin Alfa dose of 15units /kg, administered 3 times per week for 8 weeks vs no intervention | 1) **N (%) of patients with adverse events (intervention vs control)**  Hypertension: 1 (5.8%) vs 0 (0%), p-value unknown |
| 2) Razouk, 2006 | 2) Anaemic paediatric patients who received myelosupressive chemotherapy for nonmyeloid malignancies aged 5 to 18 yrs. | | 2) 222 (111 vs 111) | 2) Epoetin Alfa dose of 600units/kg to 900units/kg (if Hb had not increased by 1g/dL or more from baseline), administered intravenously 1 time per week for 16 months vs placebo administered intravenously 1 time per week for 16 months | 2) **N (%) of patients with adverse events (intervention vs control)**  Serious adverse events rate: (68.8%) vs (74.5%)   - Most-common serious adverse events in intervention group: fever (11.6%) and infection (6.3%) - Most-common serious adverse events in control group: fever (10.0%), infection (12.7%) and mucositis (5.5%)   Hypertension: 2 (1.8%) vs 1 (0.9%)  Thrombotic vascular event ≥ 1: (22.3%) vs (22.7%)  P-values unknown, unclear whether events were related to the intervention |
| **Grade assessment** | | | | | |
| Study design: | +4 | 2 Randomized Controlled Trials | | | |
| Study limitations | -2 | Serious limitations - Selection bias: Unclear in 2/2; Attrition bias: Low in 1/2 and unclear in 1/2; Performance bias: unclear in 2/2; Detection bias: unclear in 2/2 | | | |
| Consistency: | 0 | No important inconsistency. All studies report adverse events | | | |
| Directness: | 0 | Results are direct. Outcomes are generalizable. | | | |
| Precision: | 0 | No important imprecision, large sample size | | | |
| Publication bias: | 0 | Unlikely | | | |
| Effect size: | 0 | No large magnitude of effect | | | |
| Dose-response: | 0 | Unclear dose-response relationship | | | |
| Plausible confounding: | 0 | No plausible confounding | | | |
| **Quality of evidence:** |  | **⊕⊕⊖⊖ LOW** | | | |
| **Conclusion:** |  | **There is low quality of evidence that adverse effects occured in both intervention and control group. Most common adverse effects were hypertension, fever, infection and mucositis.** | | | |

| **Epoetin Alfa** | | | | | |
| --- | --- | --- | --- | --- | --- |
| Studies | Type of participants | | Total no. of participants (intervention vs control) | Type of intervention vs control | Outcome and Effect size |
| **Health Related Quality of life** PedsQL – GCS, Range of score 0-100, Higher score indicates higher Quality of Life | | | | | |
| 1) Razouk, 2006 | 1) Children with anaemia who received myelosupressive chemotherapy for nonmyeloid malignancies aged 5 to 18 yrs. | | 1) 222 (111 vs 111) | 1) Epoetin Alfa dose of 600units/kg to 900units/kg (if Hb had not increased by 1g/dL or more from baseline), administered intravenously 1 time per week for 16 months vs placebo administered intravenously 1 time per week for 16 months | **Total mean PedsQL-GCS scores at final visit (intervention vs control):**  74.9 (15.22) vs. 75.5 (15.74); EMD _intervention - control_ = -0.61 (95%CI -4.62 to  3.39), p = 0.823  **Haemoglobin levels and quality of life**  A significant correlation was found between change in Hb level and change in quality-of-life score in the intervention group (r = 0.242, p = 0.018). In the placebo group the correlation was not significant (r = 0.86, p = 0.430) |
| **Grade assessment** | | | | | |
| Study design: | +4 | 1 Randomized Controlled Trials | | | |
| Study limitations | -1 | Serious limitations – Selection bias: unclear; Attrition bias: Low; Performance bias: Unclear; Detection bias: Unclear | | | |
| Consistency: | 0 | No important inconsistency. Only 1 study performed | | | |
| Directness: | 0 | Results are direct. Outcomes generalizable. | | | |
| Precision: | -1 | No important imprecision. Only 1 study performed | | | |
| Publication bias: | 0 | Unlikely | | | |
| Effect size: | 0 | No large magnitude of effect | | | |
| Dose-response: | 0 | Unclear dose-response relationship. | | | |
| Plausible confounding: | 0 | No plausible confounding | | | |
| **Quality of evidence:** |  | **⊕⊕⊖⊖ LOW** | | | |
| **Conclusion:** |  | **There is low quality evidence there is no significant effect of Epoetin Alfa (dose starting from 600 units/kg per week) on quality-of-life scores in children with cancer- or chemotherapy induced anaemia as compared to placebo** | | | |

## *Sub-WG 1F Skin complaints*

### Naloxone

| **Naloxone** | | | | | |
| --- | --- | --- | --- | --- | --- |
| Studies | Type of participants | | Total no. of participants (intervention vs control) | Type of intervention vs control | Outcome and Effect size |
| **Incidence of pruritus** | | | | | |
| 1) Maxwell, 2005 | 1) Children with post-operative opioid-induced side effects (pruritus,) aged 6 – 18 years. | | 1) 46 (20 vs 26) | 1) 0.25 µg · kg^-1^ · h^-1^ of naloxone by continuous infusion vs placebo, saline was administered via the infusion pump. | **Incidence of pruritus control vs. intervention:**  Percentage of patients with pruritus: 77% vs 20%, p < 0.05. |
| **Grade assessment** | | | | | |
| Study design: | +4 | 1 Randomized Controlled Trials | | | |
| Study limitations | -1 | Some limitations - Selection bias: Low; Attrition bias low; Performance bias: low; Detection bias: unclear | | | |
| Consistency: | 0 | No important inconsistency. Only 1 study performed | | | |
| Directness: | -1 | Outcomes are direct. Unclear if outcomes are generalizable to children receiving palliative care. | | | |
| Precision: | -2 | Serious imprecision due to small sample sizes. Only 1 study performed | | | |
| Publication bias: | 0 | Unlikely | | | |
| Effect size: | 0 | No large magnitude of effect | | | |
| Dose-response: | 0 | Unclear dose-response relationship | | | |
| Plausible confounding: | 0 | No plausible confounding | | | |
| **Quality of evidence:** |  | **⊕⊖⊖⊖ VERY LOW** | | | |
| **Conclusion:** |  | **There is very low quality of evidence that Naloxone infusion decreases incidence of pruritus in children with post-operative opioid-induced side effects as compared to treatment with placebo.** | | | |

## *Sub-WG 1G Nausea and vomiting*

### Self-hypnosis

| **Self-hypnosis vs standard treatment** | | | | | |
| --- | --- | --- | --- | --- | --- |
| Studies | Type of participants | | Total no. of participants (intervention vs control) | Type of intervention vs control | Outcome and Effect size |
| **Supplemental anti-emetic medication usage,** supplemental use in control group was calculated by subtracting standard dose from total anti-emetic medication usage | | | | | |
| Jacknow, 1994 | 1) Newly diagnosed children with cancer aged 6 to 18 yrs. | | 20 (10 vs. 10) | Self-hypnosis was thought in two/three sessions of 45 minutes with a therapist + anti-emetic use, if necessary, vs informal conversations with the therapist during two/three sessions of 45 minutes + standard anti-emetic regimen (i.e., thiethylperazine/chloropromazine; diphenhydramine; ondansetron) | **Supplemental anti-emetic medication usage in chemotherapy course 1 (intervention vs control)**  Mean (SD): 0.17 (0.33) vs 1.01 (1.33), p <0.04  **Supplemental anti-emetic medication usage in chemotherapy course 2 (intervention vs control)**  Mean (SD): 0.34 (0.93) vs 2.10 (2.66), p<0.02 |
| **Grade assessment** | | | | | |
| Study design: | +4 | 1 Randomized Controlled Trial | | | |
| Study limitations | -2 | Serious limitations - Selection bias: Unclear; Attrition bias: low; Performance bias: high; Detection bias: unclear | | | |
| Consistency: | 0 | No important inconsistency. Only 1 study performed | | | |
| Directness: | 0 | Results are direct. Outcomes are generalizable. | | | |
| Precision: | -2 | Important imprecision due to small sample size. Only 1 study performed | | | |
| Publication bias: | 0 | Unlikely | | | |
| Effect size: | 0 | No large magnitude of effect | | | |
| Dose-response: | 0 | Unclear dose-response relationship | | | |
| Plausible confounding: | 0 | No plausible confounding | | | |
| **Quality of evidence:** |  | **⊕⊖⊖⊖ VERY LOW** | | | |
| **Conclusion:** |  | **There is very low quality of evidence that self-hypnosis decreases supplemental anti-emetic medication usage within 24h in children with cancer receiving chemotherapy as compared to standard treatment with anti-emetics.** | | | |

| **Self-hypnosis vs standard treatment** | | | | | |
| --- | --- | --- | --- | --- | --- |
| Studies | Type of participants | | Total no. of participants (intervention vs control) | Type of intervention vs control | Outcome and Effect size |
| **Nausea and vomiting,** combined score of:  (1) the severity of nausea visual analogue scale, score ranging from 0 (smiling face) to 5 (frowning face), higher score indicating higher severity of nausea  (2) frequency of vomiting and retching, score ranging from 1 (none) to 9 (all the time), higher score indicating higher frequency of vomiting. | | | | | |
| Jacknow, 1994 | Newly diagnosed children with cancer aged 6 to 18 yrs. | | 20 (10 vs. 10) | 1Self-hypnosis was thought in two/three sessions of 45 minutes with a therapist + anti-emetic use, if necessary, vs informal conversations with the therapist during two/three sessions of 45 minutes + standard anti-emetic regimen (i.e., thiethylperazine/chloropromazine; diphenhydramine; ondansetron) | **Nausea and vomiting in chemotherapy course 1 (intervention vs control)**  Mean (SD): 1.79 (1.77) vs 3.21 (2.01), p = NS  **Nausea and vomiting in chemotherapy course 2 (intervention vs control)**  Mean (SD): 1.82 (2.01) vs 3.18 (1.81), p = NS |
| **Grade assessment** | | | | | |
| Study design: | +4 | 1 Randomized Controlled Trial | | | |
| Study limitations | -2 | Serious limitations - Selection bias: Unclear; Attrition bias: low; Performance bias: high; Detection bias: unclear | | | |
| Consistency: | 0 | No important inconsistency. Only 1 study performed | | | |
| Directness: | 0 | Results are direct. Outcomes are generalizable. | | | |
| Precision: | -2 | Important imprecision due to small sample size. Only 1 study performed | | | |
| Publication bias: | 0 | Unlikely | | | |
| Effect size: | 0 | No large magnitude of effect | | | |
| Dose-response: | 0 | Unclear dose-response relationship | | | |
| Plausible confounding: | 0 | No plausible confounding | | | |
| **Quality of evidence:** |  | **⊕⊖⊖⊖ VERY LOW** | | | |
| **Conclusion:** |  | **There is very low quality of evidence that there is no significant effect of self-hypnosis on nausea and vomiting within 24h in children with cancer receiving chemotherapy as compared to standard treatment with anti-emetics.** | | | |

| **Self-hypnosis vs standard treatment** | | | | | |
| --- | --- | --- | --- | --- | --- |
| Studies | Type of participants | | Total no. of participants (intervention vs control) | Type of intervention vs control | Outcome and Effect size |
| **Anticipatory Nausea,** combined index of (1) severity of nausea, (2) frequency and (3) time of onset before chemotherapy. A constant of 2 was added to eliminate negative numbers, higher score indicating higher frequency/severity of nausea.  **Anticipatory Vomiting,** number of patients who experienced anticipatory vomiting | | | | | |
| 1) Jacknow, 1994 | 1) Newly diagnosed children with cancer aged 6 to 18 yrs. | | 1) 20 (10 vs 10) | 1) Self-hypnosis was thought in two/three sessions of 45 minutes with a therapist + anti-emetic use, if necessary, vs informal conversations with the therapist during two/three sessions of 45 minutes + standard anti-emetic regimen (i.e., thiethylperazine/chloropromazine; diphenhydramine; ondansetron) | **Anticipatory nausea 1 to 2 months post diagnosis (intervention vs control):**  Mean (SD): 0.82 (2.60) vs 3.17 (2.60), p < 0.013  **Anticipatory nausea 4 to 6 months post diagnosis (intervention vs. control***):*  Mean (SD): 1.69 (3.64) vs 2.54 (2.47), p = NS |
| **Grade assessment** | | | | | |
| Study design: | +4 | 1 Randomized Controlled Trial | | | |
| Study limitations | -2 | Serious limitations - Selection bias: Unclear; Attrition bias: low; Performance bias: high; Detection bias: unclear | | | |
| Consistency: | 0 | No important inconsistency. Only 1 study performed | | | |
| Directness: | 0 | Results are direct. Outcomes are generalizable. | | | |
| Precision: | -2 | Important imprecision due to small sample size. Only 1 study performed | | | |
| Publication bias: | 0 | Unlikely | | | |
| Effect size: | 0 | No large magnitude of effect | | | |
| Dose-response: | 0 | Unclear dose-response relationship | | | |
| Plausible confounding: | 0 | No plausible confounding | | | |
| **Quality of evidence:** |  | **⊕⊕⊖⊖ LOW** | | | |
| **Conclusion:** |  | **There is very low quality of evidence that self-hypnosis decreases anticipatory nausea 1 to 2 months post diagnosis in children with cancer receiving chemotherapy as compared to standard treatment with anti-emetics. However, no significant effect of anticipatory nausea was found 4 to 6 months post diagnosis.** | | | |

### High dose ondansetron or low dose ondansetron vs placebo

| **high dose ondansetron or low dose ondansetron vs placebo** | | | | | |
| --- | --- | --- | --- | --- | --- |
| Studies | Type of participants | | Total no. of participants (intervention vs control) | Type of intervention vs control | Outcome and Effect size |
| Emetic episodes in 24h | | | | | |
| 1) Parker, 2001 | 1) Newly diagnosed children with cancer receiving chemotherapy (lymphoid or nonlymphoid leukaemia) aged 18 months to 15 yrs. | | 1) Total patients:  26 (each patient acted as their own control)  Total intrathecal treatments:  146 (5.6 per patient) | 1) Low dose ondansetron at 0.15 mg/kg by a 15-minute intravenous infusion vs high dose ondansetron at 0.45 mg/kg by a 14-minute intravenous infusion vs placebo of normal saline.  *Each patient acted as his/her control; treatments (low dose ondansetron, high dose ondansetron, and placebo) were administered in random order for up to 6 intrathecal treatments.* | Placebo vs Low dose ondansetron  Treatments with vomiting episodes: 62.7% vs 27.7%, p<0.001, RR = 2.3  Treatments with ≥ 2 vomiting episodes: 43.1% vs 12.8% p<0.001, RR = 3.4  Treatments with ≥ 4 vomiting episodes: 25.5% vs 4.3%, p<0.005, RR = 5.8  Placebo vs High dose ondansetron  Treatments with vomiting episodes: 62.7% vs 14.6%, p<0.001, RR =4.3  Treatments with ≥ 2 vomiting episodes: 43.1% vs 6.3%, p<0.001, RR = 6.8  Treatments with ≥ 4 vomiting episodes: 25.5% vs 0%, p<0.001  Placebo vs Any dose ondansetron  Treatments with vomiting episodes: 62.7% vs 21.1%, p<0.001, RR = 3.0, reduction of RR (after pre-administrating ondansetron) 65.7%  Treatments with ≥ 2 vomiting episodes: 43.1% vs9.1%, p<0.001, RR = 4.5, reduction of RR (after pre-administrating ondansetron) 77.5%  Treatments with ≥ 4 vomiting episodes: 25.5% vs 2.1%, p<0.001, RR = 12.1, reduction of RR (after pre-administrating ondansetron) 91.6% |
| **Grade assessment** | | | | | |
| Study design: | +4 | 1 Randomized Controlled Trial | | | |
| Study limitations | -1 | Some limitations - Selection bias: unclear; Attrition bias: low; Performance bias: low; Detection bias: unclear | | | |
| Consistency: | 0 | No important inconsistency. Only 1 study performed | | | |
| Directness: | 0 | Results are direct. Outcomes are generalizable. | | | |
| Precision: | -1 | No important imprecision. Only 1 study performed | | | |
| Publication bias: | 0 | Unlikely | | | |
| Effect size: | +1 | Large magnitude of effect | | | |
| Dose-response: | 0 | Unclear dose-response relationship | | | |
| Plausible confounding: | 0 | No plausible confounding | | | |
| **Quality of evidence:** |  | **⊕⊕⊕⊖ MODERATE** | | | |
| **Conclusion:** |  | **There is moderate quality evidence that treatment with ondansetron (low and high dose) decreases the incidence of emetic episodes within 24h in children with cancer receiving chemotherapy as compared to placebo.** | | | |

### High dose ondansetron vs low dose ondansetron

| **high dose ondansetron vs low dose ondansetron** | | | | | |
| --- | --- | --- | --- | --- | --- |
| Studies | Type of participants | | Total no. of participants (intervention vs control) | Type of intervention vs control | Outcome and Effect size |
| **Emetic episodes in 24h** | | | | | |
| 1) Brock, 1996 | 1) Newly diagnosed children with cancer receiving chemotherapy aged 2 to 16 | | 1) 158 (79 vs 79)   - Cisplatin:   31 (14 vs 17)   - Ifosfamide:   28 (14 vs 14) | 1) Low dose ondansetron, 5mg/m^2^ (maximum 8 mg/m^2^) vs high dose ondansetron, 10mg/m^2^ (maximum of 16mg/m^2^) | **1) Low dose vs high dose ondansetron**  Patients with ≤ 2 emetic episodes: 71% vs 72%, p = NS.  Patients receiving cisplatin chemotherapy with ≤ 2 emetic episodes: 50% vs 53%, p = NS  Patients receiving ifosfamide with ≤ 2 emetic episodes: 79% vs 64%, p = NS |
| 2) Parker, 2001 | 2) Newly diagnosed children with cancer receiving chemotherapy (lymphoid or nonlymphoid leukaemia) aged 18 months to 15 yrs. | | 2) Total patients:  26 (each patient acted as their own control)  Total intrathecal treatments:  146 (5.6 per patient) | 2) Low dose ondansetron at 0.15 mg/kg by a 15-minute intravenous infusion vs high dose ondansetron at 0.45 mg/kg by a 14-minute intravenous infusion  *Each patient acted as his/her control; treatments (low dose ondansetron, high dose ondansetron, and placebo) were administered in random order for up to 6 intrathecal treatments.* | **2) Low dose vs. high dose ondansetron**  Treatments with vomiting episodes: 27.7% vs 14.6%, p<0.1  Treatments with ≥ 2 vomiting episodes: 12.8% vs 6.3%, p<0.3  Treatments with ≥ 4 vomiting episodes: 4.3% vs 0.0% vs, p<0.1 |
| **Grade assessment** | | | | | |
| Study design: | +4 | 2 Randomized Controlled Trials | | | |
| Study limitations | -1 | Some limitations - Selection bias: low in 1/2, unclear in 1/2; Attrition bias: high in 1/2 and low in 1/2; Performance bias: low in 2/2; Detection bias: unclear in 2/2 | | | |
| Consistency: | 0 | No important inconsistency. | | | |
| Directness: | 0 | Results are direct. Outcomes are generalizable. | | | |
| Precision: | 0 | No important imprecision, large sample size. | | | |
| Publication bias: | 0 | Unlikely | | | |
| Effect size: | 0 | No large magnitude of effect | | | |
| Dose-response: | 0 | Unclear dose-response relationship | | | |
| Plausible confounding: | 0 | No plausible confounding | | | |
| **Quality of evidence:** |  | **⊕⊕⊕⊖ MODERATE** | | | |
| **Conclusion:** |  | **There is moderate quality of evidence that there is no significant effect of treatment with high dose ondansetron on the incidence of emetic episodes within 24h in children with cancer receiving chemotherapy as compared to treatment with low dose ondansetron.** | | | |

| **high dose ondansetron vs low dose ondansetron** | | | | | |
| --- | --- | --- | --- | --- | --- |
| Studies | Type of participants | | Total no. of participants (intervention vs control) | Type of intervention vs control | Outcome and Effect size |
| **Severity of Nausea in 24h:** None: not feeling sick at all; Mild: feeling sick; Severe: feeling very sick | | | | | |
| 1) Brock, 1996 | 1) Newly diagnosed children with cancer receiving chemotherapy aged 2 to 16 | | 1) 158 (79 vs 79)   - Cisplatin:   31 (14 vs 17)   - Ifosfamide:   28 (14 vs 14) | 1) Low dose ondansetron, 5mg/m^2^ (maximum 8 mg/m^2^) vs high dose ondansetron, 10mg/m^2^ (maximum of 16mg/m^2^) | **Low dose vs high dose ondansetron**  Patients with no or mild nausea: 90% vs 86%, P = NS  Patients receiving cisplatin chemotherapy with no or mild nausea: 100% vs 86%, p = NS  Patients receiving ifosfamide with no or mild nausea: 78% vs 77%, p = NS |
| **Grade assessment** | | | | | |
| Study design: | +4 | 1 Randomized Controlled Trial | | | |
| Study limitations | -2 | Serious limitations - Selection bias: Unclear; Attrition bias: low; Performance bias: high; Detection bias: unclear | | | |
| Consistency: | 0 | No important inconsistency. Only 1 study performed | | | |
| Directness: | 0 | Results are direct. Outcomes are generalizable. | | | |
| Precision: | -1 | No important imprecision due to large sample size. Only 1 study performed | | | |
| Publication bias: | 0 | Unlikely | | | |
| Effect size: | 0 | No large magnitude of effect | | | |
| Dose-response: | 0 | Unclear dose-response relationship | | | |
| Plausible confounding: | 0 | No plausible confounding | | | |
| **Quality of evidence:** |  | **⊕⊖⊖⊖ VERY LOW** | | | |
| **Conclusion:** |  | **There is very low quality of evidence that there is no significant effect of treatment with high dose ondansetron on nausea severity within 24h in children with cancer receiving chemotherapy as compared to treatment with low dose ondansetron.** | | | |

### High dose ondansetron + dexamethasone vs low dose ondansetron + dexamethasone

| **High dose ondansetron + dexamethasone vs low dose ondansetron + dexamethasone** | | | | | |
| --- | --- | --- | --- | --- | --- |
| Studies | Type of participants | | Total no. of participants (intervention vs control) | Type of intervention vs control | Outcome and Effect size |
| Emetic episodes in 24h  Complete response: No emetic episode; Major response: 1-2 emetic episodes; Minor response: 3-5 emetic episodes; treatment failure: more than 5 emetic episodes | | | | | |
| 1) Brock, 1996 | 1) Newly diagnosed children with cancer receiving chemotherapy aged 2 to 16 | | 1) Treatment failures  34 (15 vs 19) | 1) Treatment failures:  Low dose ondansetron, 5mg/m^2^ (maximum 8 mg/m^2^) + 10mg/m^2^ dexamethasone vs  vs high dose ondansetron, 10mg/m^2^ (maximum of 16mg/m^2^) + 10mg/m^2^ dexamethasone | **Low dose vs high dose ondansetron**  Patients that were initially treatment failures^1^ (≥ 5 emetic episodes) with ≤ 2 emetic episodes: 9 (60%) vs 15 (84%), p-value unknown |
| **Grade assessment** | | | | | |
| Study design: | +4 | 1 Randomized Controlled Trial | | | |
| Study limitations | -1 | Some limitations - Selection bias: low; Attrition bias: high; Performance bias: low; Detection bias: unclear | | | |
| Consistency: | 0 | No important inconsistency. Only 1 study performed | | | |
| Directness: | 0 | Results are direct. Outcomes are generalizable. | | | |
| Precision: | -2 | Important imprecision due to small sample size. Only 1 study performed | | | |
| Publication bias: | 0 | Unlikely | | | |
| Effect size: | 0 | No large magnitude of effect | | | |
| Dose-response: | 0 | Unclear dose-response relationship | | | |
| Plausible confounding: | 0 | No plausible confounding | | | |
| **Quality of evidence:** |  | **⊕⊖⊖⊖ VERY LOW** | | | |
| **Conclusion:** |  | **There is very low-quality evidence that treatment with high dose ondansetron and dexamethasone decreases the incidence of emetic episodes within 24h in children with cancer receiving chemotherapy that initially were treatment failures (>5 emetic episodes during chemotherapy course) as compared to treatment with low dose ondansetron and dexamethasone (unclear if significant).** | | | |

^1^ Complete response: No emetic episode; Major response: 1-2 emetic episodes; Minor response: 3-5 emetic episodes; treatment failure: more than 5 emetic episodes

| **High dose ondansetron + dexamethasone vs low dose ondansetron + dexamethasone** | | | | | |
| --- | --- | --- | --- | --- | --- |
| Studies | Type of participants | | Total no. of participants (intervention vs control) | Type of intervention vs control | Outcome and Effect size |
| Severity of Nausea in 24h, None: not feeling sick at all; Mild: feeling sick; Severe: feeling very sick | | | | | |
| 1) Brock, 1996 | 1) Newly diagnosed children with cancer receiving chemotherapy aged 2 to 16 | | 1) Treatment failures  34 (15 vs 19) | 1) Treatment failures:  Low dose ondansetron, 5mg/m^2^ (maximum 8 mg/m^2^) + 10mg/m^2^ dexamethasone vs  vs high dose ondansetron, 10mg/m^2^ (maximum of 16mg/m^2^) + 10mg/m^2^ dexamethasone | **Low dose vs high dose ondansetron**  Patients that were initially treatment failures^1^ (> 5 emetic episodes) with no or mild nausea, 60% vs 84%, p = unknown |
| **Grade assessment** | | | | | |
| Study design: | +4 | 1 Randomized Controlled Trial | | | |
| Study limitations | -1 | Some - Selection bias: low; Attrition bias: high; Performance bias: low; Detection bias: unclear | | | |
| Consistency: | 0 | No important inconsistency. Only 1 study performed | | | |
| Directness: | 0 | Results are direct. Outcomes are generalizable. | | | |
| Precision: | -2 | Important imprecision due to small sample size. Only 1 study performed | | | |
| Publication bias: | 0 | Unlikely | | | |
| Effect size: | 0 | No large magnitude of effect | | | |
| Dose-response: | 0 | Unclear dose-response relationship | | | |
| Plausible confounding: | 0 | No plausible confounding | | | |
| **Quality of evidence:** |  | **⊕⊖⊖⊖ VERY LOW** | | | |
| **Conclusion:** |  | **There is very low-quality evidence that treatment with high dose ondansetron and dexamethasone decreases nausea severity within 24h in children with cancer receiving chemotherapy that initially were treatment failures (>5 emetic episodes during chemotherapy course) as compared to treatment with low dose ondansetron and dexamethasone (unclear if significant).** | | | |

### Ondansetron vs metoclopramide

| **Ondansetron vs Metoclopramide** | | | | | |
| --- | --- | --- | --- | --- | --- |
| Studies | Type of participants | | Total no. of participants (intervention vs control) | Type of intervention vs control | Outcome and Effect size |
| Emetic episodes in 24h | | | | | |
| 1) Kóseoglu, 1998 | 1) Children diagnosed with malignant disease, mean age 7.6 | | 1) Total patients:  15 (each patient received both treatments during different chemotherapy courses)  Chemotherapy treatments  64 (32 vs 32), 4.3 courses per patient | Ondansetron, 5mg/m^2^ (maximum 8mg) was administered intravenously 15 min before the chemotherapy and was continued orally (4mg/m2 per day) twice a day for 5 days vs Metoclopramide, 1mg/kg was administered intravenously 30 min before the chemotherapy and continued orally (0.14 mg/kg per day) four times a day for 5 days. To prevent side effects, diphenhydramine (5mg/kg per day) was given orally for 5 days  *Each patient acted as their own control and received both treatments (ondansetron and metoclopramide) during different chemotherapy courses.* | **Ondansetron vs metoclopramide**  *Cisplatin chemotherapy*  Treatments with 0 emetic episodes: 5 vs. 1, p < 0.05  Treatments with 1-2 emetic episodes: 3 vs 1, p = ns  Treatments with 3-5 emetic episodes: 1 vs 3, p = ns  Treatments with ≥ 5 emetic episodes: 0 vs 4, p = ns  *Non-cisplatin chemotherapy*  Treatments with 0 emetic episodes 21 vs. 17, p < 0.05  Treatments with 1-2 emetic episodes: 2 vs 1, p = ns  Treatments with 3-5 emetic episodes: 0 vs 1, p = ns  Treatments with ≥ 5 emetic episodes: 0 vs 4, p = ns |
| **Grade assessment** | | | | | |
| Study design: | +4 | 1 Randomized Controlled Trial | | | |
| Study limitations | -2 | Serious limitations - Selection bias: high; Attrition bias: low; Performance bias: high; Detection bias: unclear | | | |
| Consistency: | 0 | No important inconsistency. Only 1 study performed | | | |
| Directness: | 0 | Results are direct. Outcomes are generalizable. | | | |
| Precision: | -2 | Important imprecision due to small sample size. Only 1 study performed | | | |
| Publication bias: | 0 | Unlikely | | | |
| Effect size: | 0 | No large magnitude of effect | | | |
| Dose-response: | 0 | Unclear dose-response relationship | | | |
| Plausible confounding: | 0 | No plausible confounding | | | |
| **Quality of evidence:** |  | **⊕⊖⊖⊖ VERY LOW** | | | |
| **Conclusion:** |  | **There is very low quality of evidence that treatment with ondansetron decreases the incidence of emetic episodes within 24h in children with cancer receiving chemotherapy as compared to treatment with metoclopramide.** | | | |

| **Ondansetron vs Metoclopramide** | | | | | |
| --- | --- | --- | --- | --- | --- |
| Studies | Type of participants | | Total no. of participants (intervention vs control) | Type of intervention vs control | Outcome and Effect size |
| Severity of Nausea in 24h, No nausea; Mild nausea: without interfering daily activities; moderate nausea: Moderately interfering daily activities; serious nausea: seriously interfering daily activities | | | | | |
| 1) Kóseoglu, 1998 | 1) Children diagnosed with malignant disease, mean age 7.6 | | 1) Total patients:  15 (each patient received both treatments during different chemotherapy courses)  Chemotherapy treatments  64 (32 vs 32), 4.3 courses per patient | Ondansetron, 5mg/m^2^ (maximum 8mg) was administered intravenously 15 min before the chemotherapy and was continued orally (4mg/m2 per day) twice a day for 5 days vs Metoclopramide, 1mg/kg was administered intravenously 30 min before the chemotherapy and continued orally (0.14 mg/kg per day) four times a day for 5 days. To prevent side effects, diphenhydramine (5mg/kg per day) was given orally for 5 days  *Each patient acted as their own control and received both treatments (ondansetron and metoclopramide) during different chemotherapy courses.* | **Ondansetron vs metoclopramide**  *Cisplatin*  Treatments with no nausea: 7 vs. 0, p < 0.05  Treatments with mild nausea: 1 vs 2, p = ns  Treatments with moderate nausea: 1 vs 2, p = ns  Treatments with serious nausea: 0 vs 5, p = ns  *Non-cisplatin*  Treatments with no nausea: 22 vs. 19, p < 0.05  Treatments with mild nausea: 1 vs 1, p = ns  Treatments with moderate nausea: 0 vs 2, p = ns  Treatments with serious nausea: 5 vs 1, p = ns |
| **Grade assessment** | | | | | |
| Study design: | +4 | 1 Randomized Controlled Trial | | | |
| Study limitations | -2 | Serious limitations - Selection bias: high; Attrition bias: low; Performance bias: high; Detection bias: unclear | | | |
| Consistency: | 0 | No important inconsistency. Only 1 study performed | | | |
| Directness: | 0 | Results are direct. Outcomes are generalizable. | | | |
| Precision: | -2 | Important imprecision due to small sample size. Only 1 study performed | | | |
| Publication bias: | 0 | Unlikely | | | |
| Effect size: | 0 | No large magnitude of effect | | | |
| Dose-response: | 0 | Unclear dose-response relationship | | | |
| Plausible confounding: | 0 | No plausible confounding | | | |
| **Quality of evidence:** |  | **⊕⊖⊖⊖ VERY LOW** | | | |
| **Conclusion:** |  | **There is very low quality of evidence that treatment with ondansetron decreases the incidence of nausea severity within 24h in children with cancer receiving chemotherapy as compared to treatment with metoclopramide.** | | | |

| **Ondansetron vs. Metoclopramide** | | | | | |
| --- | --- | --- | --- | --- | --- |
| Studies | Type of participants | | Total no. of participants (intervention vs control) | Type of intervention vs control | Outcome and Effect size |
| Safety, adverse events | | | | | |
| 1) Kóseoglu, 1998 | 1) Children diagnosed with malignant disease, mean age 7.6 | | 1) Total patients:  15 (each patient received both treatments during different chemotherapy courses)  Chemotherapy treatments  64 (32 vs 32), 4.3 courses per patient | Ondansetron, 5mg/m^2^ (maximum 8mg) was administered intravenously 15 min before the chemotherapy and was continued orally (4mg/m2 per day) twice a day for 5 days vs Metoclopramide, 1mg/kg was administered intravenously 30 min before the chemotherapy and continued orally (0.14 mg/kg per day) four times a day for 5 days. To prevent side effects, diphenhydramine (5mg/kg per day) was given orally for 5 days  *Each patient acted as their own control and received both treatments (ondansetron and metoclopramide) during different chemotherapy courses.* | Ondansetron vs metoclopramide  Number chemotherapy cycles with adverse events   - Headache: 3 vs 3, P = NS - Dizziness: 0 vs 1, p = NS - Extrapyramidal reactions 0 vs 5, p < 0.05 |
| **Grade assessment** | | | | | |
| Study design: | +4 | 1 Randomized Controlled Trial | | | |
| Study limitations | -2 | Serious limitations - Selection bias: high; Attrition bias: low; Performance bias: high; Detection bias: unclear | | | |
| Consistency: | 0 | No important inconsistency. Only 1 study performed | | | |
| Directness: | 0 | Results are direct. Outcomes are generalizable. | | | |
| Precision: | -2 | Important imprecision due to small sample size. Only 1 study performed | | | |
| Publication bias: | 0 | Unlikely | | | |
| Effect size: | 0 | No large magnitude of effect | | | |
| Dose-response: | 0 | Unclear dose-response relationship | | | |
| Plausible confounding: | 0 | No plausible confounding | | | |
| **Quality of evidence:** |  | **⊕⊖⊖⊖ VERY LOW** | | | |
| **Conclusion:** |  | **There is very low-quality evidence that treatment with ondansetron causes less extrapyramidal symptoms as adverse effects in children with cancer receiving chemotherapy as compared to metoclopramide.** | | | |

### Granisetron vs ondansetron

| **Granisetron vs Ondansetron** | | | | | |
| --- | --- | --- | --- | --- | --- |
| Studies | Type of participants | | Total no. of participants (intervention vs control) | Type of intervention vs control | Outcome and Effect size |
| Emetic episodes in 24h | | | | | |
| 1) Orchard, 1994 | 1) Patients undergoing hematopoietic cell transplantations aged 2 – 65.  *Only child outcomes are used* | | 1) Children aged <18 yrs.: 51 (23 vs 28) | 1) A single intravenous granisetron dose followed by intravenous granisetron dose of 10µg/kg/dose per 12h vs an initial loading dose of ondansetron followed by continuous infusion of a 0.15 mg/kg load along with a 0.03mg/kg/h drip rounded to the nearest 0.1 mg | **Granisetron vs ondansetron**  Mean number of Emetic episodes in 24h  Mean (95%CI): 0.54 (95%CI 0.27-0.81) vs 0.87 (95%CI 0.63-1.11), p = 0.08 |
| **Grade assessment** | | | | | |
| Study design: | +4 | 1 Randomized Controlled Trial | | | |
| Study limitations | -1 | Some limitations - Selection bias: Unclear; Attrition bias: low; Performance bias: low; Detection bias: unclear | | | |
| Consistency: | 0 | No important inconsistency. Only 1 study performed | | | |
| Directness: | 0 | Results are direct. Outcomes are generalizable. | | | |
| Precision: | -2 | Important imprecision due to small sample size. Only 1 study performed | | | |
| Publication bias: | 0 | Unlikely | | | |
| Effect size: | 0 | No large magnitude of effect | | | |
| Dose-response: | 0 | Unclear dose-response relationship | | | |
| Plausible confounding: | 0 | No plausible confounding | | | |
| **Quality of evidence:** |  | **⊕⊖⊖⊖ VERY LOW** | | | |
| **Conclusion:** |  | **There is very low-quality evidence that there is no significant effect of treatment with granisetron on the incidence of emetic episodes within 24h in children with cancer receiving chemotherapy as compared to treatment with ondansetron.** | | | |

| **Granisetron vs Ondansetron** | | | | | |
| --- | --- | --- | --- | --- | --- |
| Studies | Type of participants | | Total no. of participants (intervention vs control) | Type of intervention vs control | Outcome and Effect size |
| **Severity of Nausea in 24h,** Visual Analogue scale, score ranging from 0 to 5, higher score indicating more severe nausea | | | | | |
| 1) Orchard, 1994 | 1) Patients undergoing hematopoietic cell transplantations aged 2 – 65.  *Only child outcomes are used* | | 1) Children aged <18 yrs.: 51 (23 vs 28) | 1) A single intravenous granisetron dose followed by intravenous granisetron dose of 10µg/kg/dose per 12h vs an initial loading dose of ondansetron followed by continuous infusion of a 0.15 mg/kg load along with a 0.03mg/kg/h drip rounded to the nearest 0.1 mg | **Granisetron vs ondansetron**  Mean Nausea Score:  Mean (95%CI): 0.82 (95%CI 0.55-1.09) vs 1.14 (95%CI 0.90-1.38), p = 0.09 |
| **Grade assessment** | | | | | |
| Study design: | +4 | 1 Randomized Controlled Trial | | | |
| Study limitations | -1 | Some limitations - Selection bias: Unclear; Attrition bias: low; Performance bias: low; Detection bias: unclear | | | |
| Consistency: | 0 | No important inconsistency. Only 1 study performed | | | |
| Directness: | 0 | Results are direct. Outcomes are generalizable. | | | |
| Precision: | -2 | Important imprecision due to small sample size. Only 1 study performed | | | |
| Publication bias: | 0 | Unlikely | | | |
| Effect size: | 0 | No large magnitude of effect | | | |
| Dose-response: | 0 | Unclear dose-response relationship | | | |
| Plausible confounding: | 0 | No plausible confounding | | | |
| **Quality of evidence:** |  | **⊕⊖⊖⊖ VERY LOW** | | | |
| **Conclusion:** |  | **There is very low-quality evidence that there is no significant effect of treatment with granisetron on nausea severity within 24h in children with cancer receiving chemotherapy as compared to treatment with ondansetron.** | | | |

| **Granisetron vs Ondansetron** | | | | | |
| --- | --- | --- | --- | --- | --- |
| Studies | Type of participants | | Total no. of participants (intervention vs control) | Type of intervention vs control | Outcome and Effect size |
| **Safety,** adverse events and adverse effects | | | | | |
| 1) Orchard, 1994 | 1) Patients undergoing hematopoietic cell transplantations aged 2 – 65.  *Only child outcomes are used* | | 1) 187 children and adolescents aged 2-65 (90 vs 97) | 1) A single intravenous granisetron dose followed by intravenous granisetron dose of 10µg/kg/dose per 12h vs an initial loading dose of ondansetron followed by continuous infusion of a 0.15 mg/kg load along with a 0.03mg/kg/h drip rounded to the nearest 0.1 mg | **Granisetron vs ondansetron**  Safety (children and adults)  28 (13 with headache, 6 with diarrhoea, 4 with dizziness, 5 with joint pain) vs 19 (13 with headache, 2 with diarrhoea, 2 with dizziness, 1 with joint pain).  In one case granisetron was discontinued because of headaches. |
| **Grade assessment** | | | | | |
| Study design: | +4 | 1 Randomized Controlled Trial | | | |
| Study limitations | -1 | Some limitations - Selection bias: Unclear; Attrition bias: low; Performance bias: low; Detection bias: unclear | | | |
| Consistency: | 0 | No important inconsistency. Only 1 study performed | | | |
| Directness: | -1 | Unclear if outcome is generalizable, as the outcome is measured in both children and adults. | | | |
| Precision: | -2 | Important imprecision due to small sample size. Only 1 study performed | | | |
| Publication bias: | 0 | Unlikely | | | |
| Effect size: | 0 | No large magnitude of effect | | | |
| Dose-response: | 0 | Unclear dose-response relationship | | | |
| Plausible confounding: | 0 | No plausible confounding | | | |
| **Quality of evidence:** |  | **⊕⊖⊖⊖ VERY LOW** | | | |
| **Conclusion:** |  | **There is very low-quality evidence that treatment with granisetron or ondansetron causes adverse effects in children with cancer receiving chemotherapy. It is unclear there is a significant difference between both treatment groups. Most commonly reported adverse effect was headache.** | | | |

### Granisetron vs tropisetron

| **Granisetron vs tropisetron** | | | | | |
| --- | --- | --- | --- | --- | --- |
| Studies | Type of participants | | Total no. of participants (intervention vs control) | Type of intervention vs control | Outcome and Effect size |
| Emetic episodes in 24h | | | | | |
| 1) Aksoylar, 2001 | 1) Children receiving highly emetogenic chemotherapy, aged 1 to 17 yrs. | | 1) Total patients:  51  Chemotherapy treatments:133 | A single daily dose of tropisetron of 0.2 mg/kg/day (max 5 mg) vs A single daily dose 24-h of granisetron 40 µg/kg/day (max 3 mg)  *Chemotherapy cycles were randomized 1:1 to receive either tropisetron or granisetron as anti-emetic agent.* | **Tropisetron vs granisetron**  Treatments with 0 emetic episodes: 74% vs 88%, p = 0.04  Treatments with 1-4 emetic episodes: 20% vs 12%  Treatments with > 4 emetic episodes: 6% vs 0% |
| **Grade assessment** | | | | | |
| Study design: | +4 | 1 Randomized Controlled Trial | | | |
| Study limitations | -2 | Serious limitations - Selection bias: Unclear; Attrition bias: low; Performance bias: high; Detection bias: unclear | | | |
| Consistency: | 0 | No important inconsistency. Only 1 study performed | | | |
| Directness: | 0 | Results are direct. Outcomes are generalizable. | | | |
| Precision: | -1 | No important imprecision. Only 1 study performed | | | |
| Publication bias: | 0 | Unlikely | | | |
| Effect size: | 0 | No large magnitude of effect | | | |
| Dose-response: | 0 | Unclear dose-response relationship | | | |
| Plausible confounding: | 0 | No plausible confounding | | | |
| **Quality of evidence:** |  | **⊕⊖⊖⊖ VERY LOW** | | | |
| **Conclusion:** |  | **There is very low quality of evidence that treatment with granisetron decreases the incidence of emetic episodes within 24h in children with cancer receiving chemotherapy as compared to treatment with tropisetron.** | | | |

| **Granisetron vs tropisetron** | | | | | |
| --- | --- | --- | --- | --- | --- |
| Studies | Type of participants | | Total no. of participants (intervention vs control) | Type of intervention vs control | Outcome and Effect size |
| **Severity of nausea** | | | | | |
| 1) Aksoylar, 2001 | 1) Children receiving highly emetogenic chemotherapy, aged 1 to 17 yrs. | | 1) Total patients:  51  Chemotherapy treatments:133 | A single daily dose of tropisetron of 0.2 mg/kg/day (max 5 mg) vs A single daily dose 24-h of granisetron 40 µg/kg/day (max 3 mg)  *Chemotherapy cycles were randomized 1:1 to receive either tropisetron or granisetron as anti-emetic agent.* | **Tropisetron vs granisetron**  Episodes of nausea (one episode was defined as nausea continuing for 1 hour)  Percentage of treatments with no episodes of nausea: 56% vs 82%, p = 0.002  Percentage of treatments with 1-4 episodes of nausea: 38% vs 18%  Percentage of treatments with > 4 episodes of nausea: 6% vs 0% |
| **Grade assessment** | | | | | |
| Study design: | +4 | 1 Randomized Controlled Trial | | | |
| Study limitations | -2 | Serious limitations - Selection bias: Unclear; Attrition bias: low; Performance bias: high; Detection bias: unclear | | | |
| Consistency: | 0 | No important inconsistency. Only 1 study performed | | | |
| Directness: | 0 | Results are direct. Outcomes are generalizable. | | | |
| Precision: | -1 | No important imprecision Only 1 study performed | | | |
| Publication bias: | 0 | Unlikely | | | |
| Effect size: | 0 | No large magnitude of effect | | | |
| Dose-response: | 0 | Unclear dose-response relationship | | | |
| Plausible confounding: | 0 | No plausible confounding | | | |
| **Quality of evidence:** |  | **⊕⊖⊖⊖ VERY LOW** | | | |
| **Conclusion:** |  | **There is very low quality of evidence that treatment with granisetron decreases nausea severity within 24h in children with cancer receiving chemotherapy as compared to treatment with tropisetron.** | | | |

| **Granisetron vs tropisetron** | | | | | |
| --- | --- | --- | --- | --- | --- |
| Studies | Type of participants | | Total no. of participants (intervention vs control) | Type of intervention vs control | Outcome and Effect size |
| Safety, adverse events | | | | | |
| 1) Aksoylar, 2001 | 1) Children receiving highly emetogenic chemotherapy, aged 1 to 17 yrs. | | 1) Total patients:  51  Chemotherapy treatments:133 | A single daily dose of tropisetron of 0.2 mg/kg/day (max 5 mg) vs A single daily dose 24-h of granisetron 40 µg/kg/day (max 3 mg)  *Chemotherapy cycles were randomized 1:1 to receive either tropisetron or granisetron as anti-emetic agent.* | **Tropisetron vs granisetron**  Adverse events were reported in 9 (6%) of the chemotherapy cycles (p = NS)  There were no differences in the tolerability of the two antiemetic therapy modalities (5% in tropisetron and 6% in granisetron group).  Most common effect: Headache (n = 6); constipation (n=2) |
| **Grade assessment** | | | | | |
| Study design: | +4 | 1 Randomized Controlled Trial | | | |
| Study limitations | -2 | Serious limitations - Selection bias: Unclear; Attrition bias: low; Performance bias: high; Detection bias: unclear | | | |
| Consistency: | 0 | No important inconsistency. Only 1 study performed | | | |
| Directness: | 0 | Results are direct. Outcomes are generalizable. | | | |
| Precision: | -1 | No important imprecision Only 1 study performed | | | |
| Publication bias: | 0 | Unlikely | | | |
| Effect size: | 0 | No large magnitude of effect | | | |
| Dose-response: | 0 | Unclear dose-response relationship | | | |
| Plausible confounding: | 0 | No plausible confounding | | | |
| **Quality of evidence:** |  | **⊕⊖⊖⊖ VERY LOW** | | | |
| **Conclusion:** |  | **There is very low-quality evidence that there was no significant effect of treatment with granisetron on adverse events in children with cancer receiving chemotherapy as compared to treatment with tropisetron. Most commonly reported adverse events were headache and constipation.** | | | |

### Aprepipant + Dexamethasone + ondansetron vs Dexamethasone + ondansetron

| **Aprepipant + Dexamethasone + ondansetron vs Dexamethasone + ondansetron** | | | | | |
| --- | --- | --- | --- | --- | --- |
| Studies | Type of participants | | Total no. of participants (intervention vs control) | Type of intervention vs control | Outcome and Effect size |
| Emetic episodes in 24h | | | | | |
| 1) Gore, 2009 | 1) Children with cancer who received chemotherapy aged 11 to 19 yrs. | | 1) 50 (32 vs 18) | Aprepipant (125 mg) administered 1hr before chemotherapy. Dexamethasone(8mg) + ondansetron (0.15/mg/kg x 3 doses) was administered 30min before chemotherapy vs  Placebo administered 1hr before chemotherapy. Dexamethasone(8mg) + ondansetron (0.15/mg/kg x 3 doses) was administered 30min before chemotherapy | **Aprepipant + Dexamethasone + ondansetron vs Dexamethasone + ondansetron**  Patients with 0 emetic episodes: 64.3% (95%CI 44.1% - 81.4%) vs 44.4% (95%CI 21.5 % - 69.2%) (p-value not reported) |
| **Grade assessment** | | | | | |
| Study design: | +4 | 1 Randomized Controlled Trial | | | |
| Study limitations | -1 | Some limitations - Selection bias: Unclear; Attrition bias: low; Performance bias: low; Detection bias: unclear | | | |
| Consistency: | 0 | No important inconsistency. Only 1 study performed | | | |
| Directness: | 0 | Results are direct. Outcomes are generalizable. | | | |
| Precision: | -2 | Important imprecision due to small sample size. Only 1 study performed | | | |
| Publication bias: | 0 | Unlikely | | | |
| Effect size: | 0 | No large magnitude of effect | | | |
| Dose-response: | 0 | Unclear dose-response relationship | | | |
| Plausible confounding: | 0 | No plausible confounding | | | |
| **Quality of evidence:** |  | **⊕⊖⊖⊖ VERY LOW** | | | |
| **Conclusion:** |  | **There is very low quality of evidence that treatment with aprepipant, dexamethasone and ondansetron decrease the incidence of emetic episodes within 24h in children with cancer receiving chemotherapy as compared to treatment with dexamethasone and ondansetron (unclear if significant).** | | | |

| **Aprepipant + Dexamethasone + ondansetron vs Dexamethasone + ondansetron** | | | | | |
| --- | --- | --- | --- | --- | --- |
| Studies | Type of participants | | Total no. of participants (intervention vs control) | Type of intervention vs control | Outcome and Effect size |
| Safety, adverse events | | | | | |
| 1) Gore, 2009 | 1) Children with cancer who received chemotherapy aged 11 to 19 yrs. | | 1) 16 (28 (+4) vs 18 | Aprepipant (125 mg) administered 1hr before chemotherapy. Dexamethasone(8mg) + ondansetron (0.15/mg/kg x 3 doses) was administered 30min before chemotherapy vs  Placebo administered 1hr before chemotherapy. Dexamethasone(8mg) + ondansetron (0.15/mg/kg x 3 doses) was administered 30min before chemotherapy | **Aprepipant + Dexamethasone + ondansetron vs Dexamethasone + ondansetron**  >1 clinical adverse event: 27 (84.4%) vs 13 (72.2%)  Drug related clinical adverse events (i.e., hiccups): 7 (21.9%) vs 1 (5.6%)  Serious clinical adverse events (i.e., neutropenia): 10 (31.3%) vs 3 (16.7%)  >1 laboratory adverse event (neutropenia, hypokalaemia, leukopenia): 6 (18.8%) vs 6 (33.3%)  No deaths, no discontinuation due to adverse events, no serious drug-related adverse events, no drug-related laboratory adverse events |
| **Grade assessment** | | | | | |
| Study design: | +4 | 1 Randomized Controlled Trial | | | |
| Study limitations | -1 | Some limitations - Selection bias: Unclear; Attrition bias: low; Performance bias: low; Detection bias: unclear | | | |
| Consistency: | 0 | No important inconsistency. Only 1 study performed | | | |
| Directness: | 0 | Results are direct. Outcomes are generalizable. | | | |
| Precision: | -2 | Important imprecision due to small sample size. Only 1 study performed | | | |
| Publication bias: | 0 | Unlikely | | | |
| Effect size: | 0 | No large magnitude of effect | | | |
| Dose-response: | 0 | Unclear dose-response relationship | | | |
| Plausible confounding: | 0 | No plausible confounding | | | |
| **Quality of evidence:** |  | **⊕⊖⊖⊖ VERY LOW** | | | |
| **Conclusion:** |  | **There is very low-quality evidence that treatment with aprepipant, dexamethasone and ondansetron or dexamethasone and ondansetron cause adverse effects in children with cancer receiving chemotherapy. It is unclear there is a significant difference between both treatment groups. Most commonly reported adverse effect was neutropenia** | | | |

### Midazolam vs dexamethasone vs midazolam + dexamethasone vs placebo

| **Midazolam vs dexamethasone vs midazolam + dexamethasone vs placebo** | | | | | |
| --- | --- | --- | --- | --- | --- |
| Studies | Type of participants | | Total no. of participants (intervention vs control) | Type of intervention vs control | Outcome and effect size |
| **Incidence of emetic episodes:** forceful expulsion of liquid or solid gastric contents | | | | | |
| 1) Riad, 2007 | 1) Children who were scheduled to undergo elective strabismus surgery | | 1) 100 (25 vs 25 vs 25 vs 25) | midazolam 50µgkg^-1^ vs dexamethasone 0.5mgkg^-1^ (maximum dose, 8mg) vs combination of midazolam 50µgkg^-1^ and dexamethasone 0.5mgkg^-1^ (maximum dose, 8mg) vs placebo | *Incidence post-operative vomiting*   - Group 1 – midazolam: N = 0 (0%), p < 0.001 compared with placebo, p < 0.05 compared with dexamethasone - Group 2 – dexamethasone: N = 8 (32%), p < 0.001 compared with placebo - Group 3 – Midazolam + dexamethasone: N = 0 (0%), p<0.001 compared with placebo, p < 0.05 compared with dexamethasone - Placebo: N=13 (52%) |
| **Grade assessment** | | | | | |
| Study design: | +4 | 1 Randomized Controlled Trial | | | |
| Study limitations | -1 | Some limitations - Selection bias: Low; Attrition bias: low; Performance bias: Low; Detection bias: unclear | | | |
| Consistency: | 0 | No important inconsistency. Only 1 study performed | | | |
| Directness: | -1 | Outcomes are direct. However, unclear if the population of children undergoing strabismus surgery is representative for children in palliative care. | | | |
| Precision: | -2 | Important imprecision due to small sample size. Only 1 study performed | | | |
| Publication bias: | 0 | Unlikely | | | |
| Effect size: | 0 | No large magnitude of effect | | | |
| Dose-response: | 0 | Unclear dose-response relationship | | | |
| Plausible confounding: | 0 | No plausible confounding | | | |
| **Quality of evidence:** |  | **⊕⊖⊖⊖ VERY LOW** | | | |
| **Conclusion:** |  | **There is very low quality of evidence that treatment with midazolam decreases the incidence of emetic episodes within 24h in children undergoing strabismus surgery as compared to placebo.**  **There is very low quality of evidence that treatment with dexamethasone decreases the incidence of emetic episodes within 24h in children undergoing strabismus surgery as compared to placebo.**  **There is very low quality of evidence that treatment with midazolam and dexamethasone decreases the incidence of emetic episodes within 24h in children undergoing strabismus surgery as compared to placebo.**  **There is very low quality of evidence that treatment with midazolam decreases the incidence of emetic episodes within 24h in children undergoing strabismus surgery as compared to dexamethasone.** | | | |

| **Midazolam vs dexamethasone vs midazolam + dexamethasone vs placebo** | | | | | |
| --- | --- | --- | --- | --- | --- |
| Studies | Type of participants | | Total no. of participants (intervention vs control) | Type of intervention vs control | Outcome and effect size |
| **Incidence of nausea:** subjective feeling that was reported by the patients | | | | | |
| Riad, 2007 | 1) Children who were scheduled to undergo elective strabismus surgery | | 1) 100 (25 vs 25 vs 25 vs 25) | midazolam 50µgkg^-1^ vs dexamethasone 0.5mgkg^-1^ (maximum dose, 8mg) vs combination of midazolam 50µgkg^-1^ and dexamethasone 0.5mgkg^-1^ (maximum dose, 8mg) vs placebo | *Incidence post-operative nausea*   - Group 1 – midazolam: N = 3 (12%), p < 0.001 compared with placebo, p = NS compared with dexamethasone - Group 2 – dexamethasone: N=8 (32%), p < 0.01 compared with placebo - Group 3 – Midazolam + dexamethasone N = 0 (0%), p<0.001 compared with placebo - Placebo: N=12 (48%) |
| **Grade assessment** | | | | | |
| Study design: | +4 | 1 Randomized Controlled Trial | | | |
| Study limitations | -1 | Some limitations - Selection bias: Low; Attrition bias: low; Performance bias: Low; Detection bias: unclear | | | |
| Consistency: | 0 | No important inconsistency. Only 1 study performed | | | |
| Directness: | -1 | Outcomes are direct. However, unclear if the population of children undergoing strabismus surgery is representative for children in palliative care. | | | |
| Precision: | -2 | Important imprecision due to small sample size. Only 1 study performed | | | |
| Publication bias: | 0 | Unlikely | | | |
| Effect size: | 0 | No large magnitude of effect | | | |
| Dose-response: | 0 | Unclear dose-response relationship | | | |
| Plausible confounding: | 0 | No plausible confounding | | | |
| **Quality of evidence:** |  | **⊕⊖⊖⊖ VERY LOW** | | | |
| **Conclusion:** |  | **There is very low quality of evidence that treatment with midazolam decreases the incidence of nausea within 24h in children undergoing strabismus surgery as compared to placebo**  **There is very low quality of evidence that treatment with dexamethasone decreases the incidence of nausea within 24h in children undergoing strabismus surgery as compared to placebo**  **There is very low quality of evidence that treatment with midazolam and dexamethasone decreases the incidence of nausea within 24h in children undergoing strabismus surgery as compared to placebo**  **There is very low quality of evidence that there is no significant effect of treatment with midazolam on the incidence of nausea within 24h in children undergoing strabismus surgery as compared to dexamethasone.** | | | |

## *Sub-WG 1H Neurological symptoms*

### Botulinetoxine type A injecties

| **Botulinum Toxin A injections** | | | | | |
| --- | --- | --- | --- | --- | --- |
| Studies | Type of participants | | Total no. of participants (intervention vs control) | Type of intervention vs control | Outcome and Effect size |
| **Parent reported treatment efficacy** - Canadian Occupational Performance Measure, range of score is not reported | | | | | |
| 1) Olesh, 2010  2) Copeland, 2014 | 1) Children with Cerebral Palsy (CP) aged 1 to 5 yrs.  2) Children with Cerebral palsy aged 2 to 16 yrs. | | 1) 22 (11 vs 11)  2) 41 (23 vs 18) | 1) Repeated botulinum toxin-A injections (n=3) with occupational therapy (OT) vs OT only  2) Botulinum toxin-A injection (n=1) with OT vs intramuscular sham with OT | 1)**Treatment efficacy at** **12-month follow-up:**  Estimated Mean Difference (EMD) _control – intervention_: -0.8 (95%CI -1.5 – 0.0), p = 0.04  2) **Treatment efficacy at 1 month follow-up:**  EMD _intervention - control_ = 2.2 (95%CI 0.9 – 3.5), p =0.001  **Treatment efficacy at 4-month follow-up:**  EMD _intervention - control_ = 1.2 (95%CI 0.0-2.5), p = NS |
| **Grade assessment** | | | | | |
| Study design: | +4 | 2 Randomized Controlled Trials | | | |
| Study limitations | -1 | Some limitations - Selection bias: Low in 2/2; Attrition bias low in 2/2; Performance bias high in 1/2 and low in 1/2; Detection bias: low in 1/2 and unclear in 1/2 | | | |
| Consistency: | 0 | No important inconsistency. All studies show that treatment efficacy is higher in children receiving botulinum toxin-A. In 1 study the relation at 4 months was not significant. | | | |
| Directness: | 0 | Results are direct. Outcomes are generalizable. | | | |
| Precision: | -1 | Some imprecisions due to small sample sizes | | | |
| Publication bias: | 0 | Unlikely | | | |
| Effect size: | 0 | No large magnitude of effect | | | |
| Dose-response: | 0 | Unclear dose-response relationship | | | |
| Plausible confounding: | 0 | No plausible confounding | | | |
| **Quality of evidence:** |  | **⊕⊕⊖⊖ LOW** | | | |
| **Conclusion:** |  | **There is low quality of evidence that Botulinum Toxin-A injection (n = 1 to 3) and OT in children with Cerebral Palsy increases treatment efficacy perceived by parents as compared to treatment with OT only. It is yet unclear whether this effect sustains over a longer period of time. Long-term effect might be dependent on the number of injections received by the patient.** | | | |

| **Botulinum Toxin A injections** | | | | | |
| --- | --- | --- | --- | --- | --- |
| Studies | Type of participants | | Total no. of participants (intervention vs control) | Type of intervention vs control | Outcome and Effect size |
| **Level of spasticity** Modified Tardieu scale, range of scores is not reported. | | | | | |
| Olesh, 2010 | Children with Cerebral Palsy (CP) aged 1 to 5 yrs. | | 22 (11 vs 11) | Repeated botulinum toxin-A injections (n=3) with occupational therapy (OT) vs OT only | **Level of spasticity forearm pronators at 12-month follow-up:**  EMD_control - intervention_ = 50.0 (95%CI 2.4 – 77.6), p = 0.009  **Level of spasticity wrists flexors at 12-month follow-up:**  EMD_control - intervention_ = 20.9 (95%CI 2.4 – 39.4), p = 0.029  **Level of spasticity elbow flexors at 12-month follow-up:**  EMD _control - intervention_ = 42.7 (95%CI -3.8 – 89.2), p = 0.070 |
| **Grade assessment** | | | | | |
| Study design: | +4 | 1 Randomized Controlled Trial | | | |
| Study limitations | -1 | Some limitations - Selection bias: Low; Attrition: bias low; Performance bias: high; Detection bias: unclear | | | |
| Consistency: | 0 | No important inconsistency. Only 1 study performed | | | |
| Directness: | 0 | Results are direct. Outcomes generalizable. | | | |
| Precision: | -2 | Important imprecision due to small sample size. Only 1 study performed | | | |
| Publication bias: | 0 | Unlikely | | | |
| Effect size: | 0 | No large magnitude of effect | | | |
| Dose-response: | 0 | Unclear dose-response relationship | | | |
| Plausible confounding: | 0 | No plausible confounding | | | |
| **Quality of evidence:** |  | **⊕⊖⊖⊖ VERY LOW** | | | |
| **Conclusion:** |  | **There is very low quality of evidence that repeated Botulinum toxin-A injections (n=3) and OT in children with Cerebral Palsy significantly decrease spasticity levels in upper limbs (forearm and wrist) as compared to treatment with OT only.** | | | |

| **Botulinum Toxin A injections** | | | | | |
| --- | --- | --- | --- | --- | --- |
| Studies | Type of participants | | Total no. of participants (intervention vs control) | Type of intervention vs control | Outcome and Effect size |
| **Level of motor performance** Quality of Upper Extremity Skills Test (QUEST) and Peabody Development Motor Scales – Fine motor (PDMS-FM) Range of score is not reported | | | | | |
| Olesh, 2010 | Children with Cerebral Palsy (CP) aged 1 to 5 yrs. | | 22 (11 vs 11) | 1) Repeated botulinum toxin-A injections (n=3) with occupational therapy (OT) vs OT only | **level of motor performance assessed by QUEST^3^ at 12-month follow-up:**  EMD _control – intervention_= -6.7 (-15.5 to 17.6), p = 0.833  **level of motor performance assessed by PDMS-FM^3^ at 12-month follow-up:**  EMD _control – intervention_ = -5.0 (-37.6 to 27.6), p = 0.753 |
| **Grade assessment** | | | | | |
| Study design: | +4 | 1 Randomized Controlled Trial | | | |
| Study limitations | -1 | Some limitations - Selection bias: Low; Attrition: bias low; Performance bias: high; Detection bias: unclear | | | |
| Consistency: | 0 | No important inconsistency. Only 1 study performed | | | |
| Directness: | 0 | Results are direct. Outcomes generalizable. | | | |
| Precision: | -2 | Important imprecision due to small sample size. Only 1 study performed | | | |
| Publication bias: | 0 | Unlikely | | | |
| Effect size: | 0 | No large magnitude of effect | | | |
| Dose-response: | 0 | Unclear dose-response relationship | | | |
| Plausible confounding: | 0 | No plausible confounding | | | |
| **Quality of evidence:** |  | **⊕⊖⊖⊖ VERY LOW** | | | |
| **Conclusion:** |  | **There is very low quality of evidence that there is no significant effect of repeated botulinum toxin A injections (n = 3) and OT on motor performance in children with Cerebral Palsy as compared to treatment with OT only.** | | | |

| **Botulinum Toxin A injections** | | | | | |
| --- | --- | --- | --- | --- | --- |
| Studies | Type of participants | | Total no. of participants (intervention vs control) | Type of intervention vs control | Outcome and Effect size |
| **Quality of life** Cerebral Palsy Quality of Life Questionnaire for children (CPQL-child), Range of score is not reported, Positive value indicates improvement in score | | | | | |
| 1) Copeland, 2014 | 1) Children with Cerebral palsy aged 2 to 16 yrs. | | 1) 41 (23 vs 18) | 1) Botulinum toxin-A injection (n=1) with OT vs intramuscular sham with OT | 1) **Quality of Life at 1 month follow-up:**  EMD _intervention – control_ = 3.7 (95%CI -0.5 - 8.0); p=. NS  **Quality of Life at 4-month follow-up:**  EMD _intervention – control_ = 2.0 (95%CI -2.9 – 6.8); p= NS |
| **Grade assessment** | | | | | |
| Study design: | +4 | 1 Randomized Controlled Trials | | | |
| Study limitations | 0 | No important limitations - Selection bias: Low; Attrition: bias low; Performance bias: low; Detection bias: low | | | |
| Consistency: | 0 | No important inconsistency. Only 1 study performed | | | |
| Directness: | 0 | Results are direct. Outcomes generalizable. | | | |
| Precision: | -2 | Some imprecisions. Only 1 study performed | | | |
| Publication bias: | 0 | Unlikely | | | |
| Effect size: | 0 | No large magnitude of effect | | | |
| Dose-response: | 0 | Unclear dose-response relationship. | | | |
| Plausible confounding: | 0 | No plausible confounding | | | |
| **Quality of evidence:** |  | **⊕⊕⊖⊖ LOW** | | | |
| **Conclusion:** |  | **There is low quality of evidence that there is no significant effect of botulinum toxin-A injection with OT on quality of life in children with Cerebral Palsy as compared to treatment with intramuscular sham and OT** | | | |

## *Sub WG 1I Pain*

### Cognitive behavourial therapy

| **Cognitive behavioural therapy for parents of children with a chronic illness** | | | | | |
| --- | --- | --- | --- | --- | --- |
| Studies | Type of participants | | Total no. of participants (intervention vs control) | Type of intervention vs control | Outcome and Effect size |
| **Child symptoms**, post-treatment | | | | | |
| 12 RCTs extracted from systematic review of RCTs: Eccleston, 2015 | Parents of children aged 0 to 19 with a chronic illness (i.e., painful conditions, cancer, diabetes, asthma, traumatic brain injury) | | Total participants 754 (396 vs 358) | Cognitive behavioural therapy for parents’ vs control (active treatment group, treatment-as-usual, waiting list control) | **Child symptoms – post treatment**  Overall effect of CBT was beneficial (SMD = -0.32, 95%CI -0.53 to -0.11, p <0.01 |
| **Grade assessment** | | | | | |
| Study design: | +4 | 12 Randomized Controlled Trials (results extracted from systematic review of RCTs: Eccleston, 2015) | | | |
| Study limitations | -2 | Some limitations - Selection bias: low in 4/12 studies and unclear 8/12 studies; Attrition bias: low in 5/12, unclear in 3/12 and high in 4/12; Performance bias: unknown; Detection bias: low in 4/12 and unclear in 8/12; | | | |
| Consistency: | 0 | No important inconsistency, I^2^ =47% | | | |
| Directness: | -1 | Outcomes are direct. It is unclear whether outcomes are generalizable to all children receiving palliative care as not all chronic conditions described are life-limiting/life-threatening. | | | |
| Precision: | 0 | No imprecision, large sample size, | | | |
| Publication bias: | 0 | Unlikely | | | |
| Effect size: | 0 | No large magnitude of effect | | | |
| Dose-response: | 0 | Unclear dose-response relationship | | | |
| Plausible confounding: | 0 | No plausible confounding | | | |
| **Quality of evidence:** |  | **⊕⊖⊖⊖ VERY LOW** | | | |
| **Conclusion:** |  | **There is very low quality of evidence that cognitive behavioural therapy for parents of children with a chronic illness decreases child symptoms post-treatment as compared to treatment as usual, active control or wait-list control.** | | | |

| **Cognitive behavioural therapy for parents of children with a chronic illness** | | | | | |
| --- | --- | --- | --- | --- | --- |
| Studies | Type of participants | | Total no. of participants (intervention vs control) | Type of intervention vs control | Outcome and Effect size |
| **Child symptoms**, follow-up | | | | | |
| 7 RCTs extracted from systematic review of RCTs: Eccleston, 2015 | Parents of children aged 0 to 19 with a chronic illness (i.e., painful conditions, cancer, diabetes, asthma, traumatic brain injury) | | Total participants 475 (253 vs 219) | Cognitive behavioural therapy (CBT) for parents’ vs control (active treatment group, treatment-as-usual, waiting list control) | **Child symptoms– follow-up**  No effect of CBT was identified. SMD -0.34 95%CI -0.73 to 0.05, z = 0.45, p = 0.65) z = 1.70. p = 0.09) |
| **Grade assessment** | | | | | |
| Study design: | +4 | 7 Randomized Controlled Trials (results extracted from systematic review of RCTs: Eccleston, 2015) | | | |
| Study limitations | -2 | Serious limitations - Selection bias: low in 3/7 studies and unclear 4/7 studies; Attrition bias: low in 4/7, unclear in 1/7 and high in 2/7; Performance bias: unknown; Detection bias: low in 2/7 and unclear in 5/7; | | | |
| Consistency: | -1 | Some inconsistency, I^2^ =74% | | | |
| Directness: | -1 | Outcomes are direct. It is unclear whether outcomes are generalizable to all children receiving palliative care as not all chronic conditions described are life-limiting/life-threatening. | | | |
| Precision: | 0 | No imprecision, large sample size. | | | |
| Publication bias: | 0 | Unlikely | | | |
| Effect size: | 0 | No large magnitude of effect | | | |
| Dose-response: | 0 | Unclear dose-response relationship | | | |
| Plausible confounding: | 0 | No plausible confounding | | | |
| **Quality of evidence:** |  | **⊕⊖⊖⊖ VERY LOW** | | | |
| **Conclusion:** |  | **There is very low quality of evidence that there is no significant effect of cognitive behavioural therapy for parents of children with a chronic illness on child symptoms at follow-up as compared to treatment as usual, active control or wait-list control.** | | | |

### Family therapy

| **Family therapy for parents of children with a chronic illness** | | | | | |
| --- | --- | --- | --- | --- | --- |
| Studies | Type of participants | | Total no. of participants (intervention vs control) | Type of intervention vs control | Outcome and Effect size |
| **Child symptoms**, post-treatment | | | | | |
| 5 RCTs extracted from systematic review of RCTs: Eccleston, 2015 | Parents of children aged 0 to 19 with a chronic illness (i.e., painful conditions, cancer, diabetes, asthma, traumatic brain injury) | | Total participants 259 (134 vs 125) | Family therapy for parents’ vs control (active treatment group, treatment-as-usual, waiting list control) | **Child symptoms – post-treatment**  No effect of family therapy was found. SMD: 0.04 95%CI -0.20 to 0.29, z = 0.35. p = 0.73 |
| **Grade assessment** | | | | | |
| Study design: | +4 | 5 Randomized Controlled Trials (results extracted from systematic review of RCTs: Eccleston, 2015) | | | |
| Study limitations | -2 | Serious limitations - Selection bias: unclear 5/5 studies; Attrition bias: unclear in 5/5; Performance bias: unknown; Detection bias: low in 2/5 and unclear in 3/5; | | | |
| Consistency: | 0 | No important inconsistency, I^2^ =1% | | | |
| Directness: | -1 | Outcomes are direct. It is unclear whether outcomes are generalizable to all children receiving palliative care as not all chronic conditions described are life-limiting/life-threatening. | | | |
| Precision: | 0 | No imprecision, large sample size | | | |
| Publication bias: | 0 | Unlikely | | | |
| Effect size: | 0 | No large magnitude of effect | | | |
| Dose-response: | 0 | Unclear dose-response relationship | | | |
| Plausible confounding: | 0 | No plausible confounding | | | |
| **Quality of evidence:** |  | **⊕⊖⊖⊖ VERY LOW** | | | |
| **Conclusion:** |  | **There is very low quality of evidence that there is no significant effect of family therapy for parents of children with a chronic illness on child symptoms post-treatment as compared to treatment as usual, active control or wait-list control.** | | | |

| **Family therapy for parents of children with a chronic illness** | | | | | |
| --- | --- | --- | --- | --- | --- |
| Studies | Type of participants | | Total no. of participants (intervention vs control) | Type of intervention vs control | Outcome and Effect size |
| **Child symptoms**, follow-up | | | | | |
| 2 RCTs extracted from systematic review of RCTs: Eccleston, 2015 | Parents of children aged 0 to 19 with a chronic illness (i.e., painful conditions, cancer, diabetes, asthma, traumatic brain injury) | | Total participants 96 (48 vs 48) | Family therapy for parents’ vs control (active treatment group, treatment-as-usual, waiting list control) | **Child symptoms – follow-up**  No effect of Family Therapy was identified. SMD: -0.02 95%CI -0.43 to 0.38, z = 0.12. p = 0.91 |
| **Grade assessment** | | | | | |
| Study design: | +4 | 2 Randomized Controlled Trials (results extracted from systematic review of RCTs: Eccleston, 2015018) | | | |
| Study limitations | -1 | Some limitations - Selection bias: unclear 2/2 studies; Attrition bias: unclear in 2/2 Performance bias: unknown; Detection bias: low in 2/2; | | | |
| Consistency: | 0 | No important inconsistency, I^2^ =0% | | | |
| Directness: | -1 | Outcomes are direct. It is unclear whether outcomes are generalizable to all children receiving palliative care as not all chronic conditions described are life-limiting/life-threatening. | | | |
| Precision: | -1 | some imprecision, small sample size | | | |
| Publication bias: | 0 | Unlikely | | | |
| Effect size: | 0 | No large magnitude of effect | | | |
| Dose-response: | 0 | Unclear dose-response relationship | | | |
| Plausible confounding: | 0 | No plausible confounding | | | |
| **Quality of evidence:** |  | **⊕⊖⊖⊖ VERY LOW** | | | |
| **Conclusion:** |  | **There is very low quality of evidence that there is no significant effect of family therapy for parents of children with a chronic illness on child symptoms at follow-up as compared to treatment as usual, active control or wait-list control.** | | | |

### Problem solving therapy

| **Problem solving therapy for parents of children with a chronic illness** | | | | | |
| --- | --- | --- | --- | --- | --- |
| Studies | Type of participants | | Total no. of participants (intervention vs control) | Type of intervention vs control | Outcome and Effect size |
| **Child symptoms**, post-treatment | | | | | |
| 2 RCTs extracted from systematic review of RCTs: Eccleston, 2015 | Parents of children aged 0 to 19 with a chronic illness (i.e., painful conditions, cancer, diabetes, asthma, traumatic brain injury) | | Total participants 216 (105 vs 111) | Problem solving therapy for parents’ vs control (active treatment group, treatment-as-usual, waiting list control) | **Child symptoms, post-treatment**  No effect of problem-solving therapy. SMD 0.19, 95%Ci -0.08 to 0.46, z = 1.41, p = 0.59 |
| **Grade assessment** | | | | | |
| Study design: | +4 | 2 Randomized Controlled Trials (results extracted from systematic review of RCTs: Eccleston, 2015) | | | |
| Study limitations | -1 | Some limitations - Selection bias: low in 1/2 and unclear 1/2 studies; Attrition bias: low in 1/2 and high in 1/2; Performance bias: unknown; Detection bias: low in 2/2; | | | |
| Consistency: | 0 | No important inconsistency, I^2^ =18% | | | |
| Directness: | -1 | Outcomes are direct. It is unclear whether outcomes are generalizable to all children receiving palliative care as not all chronic conditions described are life-limiting/life-threatening. | | | |
| Precision: | 0 | No imprecision, large sample size. | | | |
| Publication bias: | 0 | Unlikely | | | |
| Effect size: | 0 | No large magnitude of effect | | | |
| Dose-response: | 0 | Unclear dose-response relationship | | | |
| Plausible confounding: | 0 | No plausible confounding | | | |
| **Quality of evidence:** |  | **⊕⊕⊖⊖ LOW** | | | |
| **Conclusion:** |  | **There is low quality of evidence that there is no significant effect of problem-solving therapy for parents of children with a chronic illness on child symptoms at post-treatment as compared to treatment as usual, active control or wait-list control.** | | | |

### Multi-systemic therapy

| **Multi-systemic therapy for parents of children with a chronic illness** | | | | | |
| --- | --- | --- | --- | --- | --- |
| Studies | Type of participants | | Total no. of participants (intervention vs control) | Type of intervention vs control | Outcome and Effect size |
| **Child symptoms**, post-treatment | | | | | |
| 4 RCTs extracted from systematic review of RCTs: Eccleston, 2015 | Parents of children aged 0 to 19 with a chronic illness (i.e., painful conditions, cancer, diabetes, asthma, traumatic brain injury) | | Total participants 455 (130 vs 225) | Multi-systemic therapy for parents’ vs control (active treatment group, treatment-as-usual, waiting list control) | **Child symptoms, post-treatment**  No effect of multi-systemic therapy. SMD -0.24, 95%CI -0.56 to 0.07, z = 1.52, p = 0.13 |
| **Grade assessment** | | | | | |
| Study design: | +4 | 2 Randomized Controlled Trials (results extracted from systematic review of RCTs: Eccleston, 2015) | | | |
| Study limitations | -1 | Some limitations - Selection bias: low in 1/4 and unclear 3/4 studies; Attrition bias: low in 3/4 and unclear in 1/4; Performance bias: unknown; Detection bias: low in 3/4 and unclear in 1/4; | | | |
| Consistency: | -1 | Some inconsistency, I^2^ =60% | | | |
| Directness: | -1 | Outcomes are direct. It is unclear whether outcomes are generalizable to all children receiving palliative care as not all chronic conditions described are life-limiting/life-threatening. | | | |
| Precision: | 0 | No imprecision, large sample size | | | |
| Publication bias: | 0 | Unlikely | | | |
| Effect size: | 0 | No large magnitude of effect | | | |
| Dose-response: | 0 | Unclear dose-response relationship | | | |
| Plausible confounding: | 0 | No plausible confounding | | | |
| **Quality of evidence:** |  | **⊕⊕⊖⊖ VERY LOW** | | | |
| **Conclusion:** |  | **There is very low quality of evidence that there is no significant effect of multi-systemic therapy for parents of children with a chronic illness on child symptoms post-treatment as compared to treatment as usual, active control or wait-list control.** | | | |

| **Multi-systemic therapy for parents of children with a chronic illness** | | | | | |
| --- | --- | --- | --- | --- | --- |
| Studies | Type of participants | | Total no. of participants (intervention vs control) | Type of intervention vs control | Outcome and Effect size |
| **Child symptoms**, follow-up | | | | | |
| 2 RCTs extracted from systematic review of RCTs: Eccleston, 2015 | Parents of children aged 0 to 19 with a chronic illness (i.e., painful conditions, cancer, diabetes, asthma, traumatic brain injury) | | Total participants 247 (123 vs 124) | Multi-systemic therapy for parents’ vs control (active treatment group, treatment-as-usual, waiting list control) | **Child symptoms, follow-up**  No effect of multi-systemic therapy. SMD -0.19, 95%CI -0.44 to 0.09, z = 1.47, p = 0.14) |
| **Grade assessment** | | | | | |
| Study design: | +4 | 2 Randomized Controlled Trials (results extracted from systematic review of RCTs: Eccleston, 2015) | | | |
| Study limitations | -1 | Some limitations - Selection bias: low in 1/2 and unclear 1/2 studies; Attrition bias: low in 1/2 and unclear in 1/2; Performance bias: unknown; Detection bias: low in 1/2 and unclear in 1/2; | | | |
| Consistency: | 0 | No important inconsistency, I^2^ =0% | | | |
| Directness: | -1 | Outcomes are direct. It is unclear whether outcomes are generalizable to all children receiving palliative care as not all chronic conditions described are life-limiting/life-threatening. | | | |
| Precision: | 0 | No imprecision, large sample size | | | |
| Publication bias: | 0 | Unlikely | | | |
| Effect size: | 0 | No large magnitude of effect | | | |
| Dose-response: | 0 | Unclear dose-response relationship | | | |
| Plausible confounding: | 0 | No plausible confounding | | | |
| **Quality of evidence:** |  | **⊕⊕⊖⊖ LOW** | | | |
| **Conclusion:** |  | **There is low quality of evidence that there is no significant effect of multi-systemic therapy for parents of children with a chronic illness on child symptoms at follow-up as compared to treatment as usual, active control or wait-list control.** | | | |

### Opioids

| **Opioids** | | | |
| --- | --- | --- | --- |
| Studies | Type and number of studies | | Conclusions |
| Wiffen, 2011 | 0 randomized controlled trials | | No randomised controlled trials to support or refute the use of opioids to treat cancer pain in children and adolescents were identified. Following inclusion criteria were used: randomized controlled trials with or without blinding; infants, children and adolescents aged 0 to 17; studies reporting interventions prescribing opioid drug (alone or in combination) for cancer pain; and studies reporting pain assessment. |
| **Conclusion:** |  | **Unknown effects of opioids to treat cancer pain in children aged 0 to 17.** | |

### Intrathecal baclofen

| **Intrathecal baclofen** | | | | | |
| --- | --- | --- | --- | --- | --- |
| Studies | Type of participants | | Total no. of participants | Type of intervention vs control | Outcome and Effect size |
| **Pain** | | | | | |
| 3 RCTs extracted from systematic review of RCTs: Beecham, 2015.  Included RCTs:   - Bonouvrie, 2011 - Hoving, 2007 - Hoving, 2009 | Children with Cerebral Palsy (CP) aged 7 to 17 | | Total participants 21  N = 4 (Bonouvrie 2011)  N = 17 (Hoving, 2007; Hoving 2009) | Intrathecal baclofen vs placebo (Bonouvrie, 2011)  Intrathecal baclofen vs therapy as normal (Hoving, 2007; Hoving 2009) | **Pain measured using Visual Analogue Scale (VAS) (0-10):**   - Significant decrease of pain after administration of intrathecal baclofen in the intervention group compared to standard therapy in the control group. Mean Difference: 4.20, 95%CI 2.1 to 6.25 (Hoving, 2009) - Decrease of pain with 2.6 points in the intervention groups. Pain scores increased in the placebo group (Bonouvrie, 2011)   **Pain measured using VAS (0-10) at 6-month follow-up**: Significant decrease of pain in the intervention group as compared to placebo. Mean difference: 4.20, 95%CI 2.15 to 6.25 (Hoving, 2007)  **Bodily pain or discomfort measured using Child Health Questionnaire-parent form at 6-months follow-up:** Decrease of pain in the intervention group. Mean difference 26.60, 95% CI 2.61 to 50.59 (Hoving, 2007). |
| **Grade assessment** | | | | | |
| Study design: | +4 | 3 Randomized Controlled Trials (results extracted from systematic review of RCTs: Beecham, 2015) | | | |
| Study limitations | -2 | Serious limitations - Selection bias: Low in 2/3 and unclear in 1/3; Attrition bias low in 1/3, unclear in 1/3 and high in 1/3; Performance bias low in 1/3, unclear in 1/3 and high in 1/3; Detection bias: low in 1/3, unclear in 1/3 and high in 1/3; | | | |
| Consistency: | 0 | No important inconsistency. All studies show that pain scores decreased in children receiving intrathecal baclofen. | | | |
| Directness: | 0 | Results are direct. Outcomes are generalizable. | | | |
| Precision: | -1 | Some imprecisions due to small sample size (n = 21) | | | |
| Publication bias: | 0 | Unlikely | | | |
| Effect size: | 0 | No large magnitude of effect | | | |
| Dose-response: | 0 | Unclear dose-response relationship | | | |
| Plausible confounding: | 0 | No plausible confounding | | | |
| **Quality of evidence:** |  | **⊕⊖⊖⊖ VERY LOW** | | | |
| **Conclusion:** |  | **There is very low quality of evidence that treatment with intrathecal baclofen decreases pain in children with Cerebral Palsy as compared to standard treatment or placebo.** | | | |

| **Intrathecal baclofen** | | | | | |
| --- | --- | --- | --- | --- | --- |
| Studies | Type of participants | | Total no. of participants | Type of intervention vs control | Outcome and Effect size |
| **Safety,** Adverse events and adverse effects | | | | | |
| 3 RCTs extracted from systematic review of RCTs: Beecham, 2015.  Included RCTs:   - Bonouvrie, 2011 - Hoving, 2007 - Hoving, 2009 | Children with Cerebral Palsy (CP) aged 7 to 17 | | Total participants 21  N = 4 (Bonouvrie 2011)  N = 17 (Hoving, 2007; Hoving 2009) | Intrathecal baclofen vs placebo (Bonouvrie, 2011)  Intrathecal baclofen vs therapy as normal (Hoving, 2007; Hoving 2009) | **Number and type of adverse effects**   - Nine adverse effects in 8 of 17 participant, mostly related to Cerebrospinal Fluid (CSF leakage) (Hoving, 2007) - Fourteen of 17 patients experienced a total of 28 procedure or device related adverse events, mostly related to swelling at pump site (Hoving, 2009) - 2 of 4 patients experienced CSF leakage which in discontinuation of trial in one patient (Bonouvrie, 2011)   **Most common adverse effect**   - Most common adverse effect irrespective of treatment arm was related to CSF leakage, respectively 2 patients (Bonouvrie, 2011) and 3 patients (Hoving, 2007) |
| **Grade assessment** | | | | | |
| Study design: | +4 | 3 Randomized Controlled Trials (results extracted from systematic review of RCTs: Beecham, 2015) | | | |
| Study limitations | -2 | Serious limitations - Selection bias: Low in 2/3 and unclear in 1/3; Attrition bias low in 1/3, unclear in 1/3 and high in 1/3; Performance bias low in 1/3, unclear in 1/3 and high in 1/3; Detection bias: low in 1/3, unclear in 1/3 and high in 1/3; | | | |
| Consistency: | 0 | No important inconsistency. | | | |
| Directness: | 0 | Results are direct. Outcomes are generalizable. | | | |
| Precision: | -1 | Some imprecisions due to small sample size (n = 21) | | | |
| Publication bias: | 0 | Unlikely | | | |
| Effect size: | 0 | No large magnitude of effect | | | |
| Dose-response: | 0 | Unclear dose-response relationship | | | |
| Plausible confounding: | 0 | No plausible confounding | | | |
| **Quality of evidence:** |  | **⊕⊖⊖⊖ VERY LOW** | | | |
| **Conclusion:** |  | **There is very low quality of evidence that adverse effects were reported in both intervention and control group. Most common adverse effects were related to Cerebrospinal Fluid Leakage.** | | | |

### Botuline toxine type A injecties

| **Botulinum toxin A injections** | | | | | |
| --- | --- | --- | --- | --- | --- |
| Studies | Type of participants | | Total no. of participants | Type of intervention vs control | Outcome and Effect size |
| **Pain** | | | | | |
| N = 2 RCTs extracted from systematic review of RCTs: Beecham, 2015.  Included RCTs:   - Copeland, 2014 - Russo 2007 | Children with CP aged 2 to 16 | | Total participants: 84  N = 41 (Copeland, 2014)  N = 43 (Russo, 2007) | Botulinum Toxin A vs. placebo (Copeland, 2014)  Botulinum Toxin A with Occupational Therapy (OT) vs. OT only (Russo, 2007) | - **Pain measured using the Pediatric Pain Profile at 1 month follow-up:** No significant difference in pain scores between intervention and control group. Mean Difference -2.67, 95% CI -10.18 to 4.84 (Copeland, 2014) - **Pain measured using the Pediatric Pain Profile at 4-month follow-up:** No significant difference in pain scores between intervention and control group. Mean Difference 2.59, 95% CI -3.75 to 8.93 (Copeland, 2014) - **Pain measured using VAS at 3-month follow-up (2 participants in each group):** No significant difference in pain scores between intervention and control group. OR 1.05, 95%CI 0.13 to 8.24 (Russo, 2007) - **Pain measured using VAS at 6-month follow-up (1 participants in each group):** No significant difference in pain scores between intervention and control group. OR 1.05, 95% CI 0.06to 17.95 (Russo, 2007) |
| **Grade assessment** | | | | | |
| Study design: | +4 | 2 Randomized Controlled Trials (results extracted from systematic review of RCTs: Beecham, 2015) | | | |
| Study limitations | -2 | Serious limitations - Selection bias: Low in 1/2 and unclear in 1/2; Attrition bias: Low in 2/2; Performance bias: Low in 1/2 and high in 1/2; Detection bias: Low in 1/2 and high in 1/2; | | | |
| Consistency: | 0 | No important inconsistency. All studies show that there is no effect of treatment with Botulinum Toxin A on pain. In 1 study the relation at 4 months was not significant. | | | |
| Directness: | 0 | Results are direct. Outcomes are generalizable. | | | |
| Precision: | -1 | Some imprecisions due to small sample sizes | | | |
| Publication bias: | 0 | Unlikely | | | |
| Effect size: | 0 | No large magnitude of effect | | | |
| Dose-response: | 0 | Unclear dose-response relationship | | | |
| Plausible confounding: | 0 | No plausible confounding | | | |
| **Quality of evidence:** |  | **⊕⊖⊖⊖ VERY LOW** | | | |
| **Conclusion:** |  | **There is very low quality of evidence there is no significant effect of treatment with Botulinum Toxin A (with OT) on pain in children with Cerebral Palsy as compared to placebo or treatment with OT only.** | | | |

| **Botulinum toxin A injections** | | | | | |
| --- | --- | --- | --- | --- | --- |
| Studies | Type of participants | | Total no. of participants | Type of intervention vs control | Outcome and Effect size |
| **Safety,** adverse events and adverse effects | | | | | |
| N = 2 RCTs extracted from systematic review of RCTs: Beecham, 2015.  Included RCTs:   - Copeland, 2014 - Russo 2007 | Children with CP aged 2 to 16 | | Total participants: 84  N = 41 (Copeland, 2014)  N = 43 (Russo, 2007) | Botulinum Toxin A vs. placebo (Copeland, 2014)  Botulinum Toxin A with Occupational Therapy (OT) vs. OT only (Russo, 2007) | **Number and type of participants with adverse events (intervention vs control)**   - 1 participant with epilepsy and hospital admission vs 2 participants with hospital admission due to epilepsy (Russo, 2007) - 3 participants with systemic drooling, decreased vocalization or drooling vs 1 participant (Copeland, 2014)   **Number and type of adverse effects (intervention vs control)**   - 22 adverse effects (feeling unwell) vs 0 adverse effects (Russo, 2007) - 23 patients with moderate or mild adverse effects (Copeland, 2014)   **Most common adverse effect**  Most common reported effect were seizures and respiratory symptoms |
| **Grade assessment** | | | | | |
| Study design: | +4 | 2 Randomized Controlled Trials (results extracted from systematic review of RCTs: Beecham, 2015) | | | |
| Study limitations | -2 | Serious limitations - Selection bias: Low in 1/2 and unclear in 1/2; Attrition bias: Low in 2/2; Performance bias: Low in 1/2 and high in 1/2; Detection bias: Low in 1/2 and high in 1/2; | | | |
| Consistency: | 0 | No important inconsistency. | | | |
| Directness: | 0 | Results are direct. Outcomes are generalizable. | | | |
| Precision: | -1 | Some imprecisions due to small sample sizes | | | |
| Publication bias: | 0 | Unlikely | | | |
| Effect size: | 0 | No large magnitude of effect | | | |
| Dose-response: | 0 | Unclear dose-response relationship | | | |
| Plausible confounding: | 0 | No plausible confounding | | | |
| **Quality of evidence:** |  | **⊕⊖⊖⊖ VERY LOW** | | | |
| **Conclusion:** |  | **There is very low quality of evidence that adverse effects were reported in both intervention and control groups. Most common adverse effects were seizures and respiratory symptoms.** | | | |

### Oral alendronate

| **Oral alendronate** | | | | | |
| --- | --- | --- | --- | --- | --- |
| Studies | Type of participants | | Total no. of participants | Type of intervention vs control | Outcome and Effect size |
| **Pain** | | | | | |
| N = 2 RCTs extracted from systematic review of RCTs: Beecham, 2015.  Included RCTs:   - Seikaly, 2005 (cross-over RCT) - Ward, 2011 | Children with Osteogenesis imperfecta (OI) aged 3 to 19. | | Total participants 159  N = 20 (Seikaly, 2005)  N = 139 (Ward, 2011) | Oral alendronate vs placebo | - **Pain measured by number of pain-free days per month at 12-month follow-up:** Significant decrease of pain in the intervention group. Mean difference, MD-3.63, 95%CI -5.17 to -2.09 (Seikaly, 2005) - **Pain measured by number of days with analgesic use for skeletal pains at 12-month follow up:** Significant decrease of analgesic use in the intervention group. Mean Difference, -2.00, 95% CI -3.57 to -0.43 (Seikaly, 2005) - **Pain measured by number of patients with bone pain at 24-month follow-up:** In the intervention group fewer patients experienced pain in comparison to placebo (37%, 38/102 vs. 57%, 17/30). This effect was not statistically significant. OR, 0.45, 95% CI 0.20 to 1.04 (Ward, 2011) - **Pain measured by number of days per week that patients experienced bone pain at 24-month follow-up:** No significant difference the intervention group at baseline and follow-up (Ward, 2011). |
| **Grade assessment** | | | | | |
| Study design: | +4 | 2 Randomized Controlled Trials (results extracted from systematic review of RCTs: Beecham, 2015) | | | |
| Study limitations | -1 | Some limitations - Selection bias: Low in 1/2 and unclear in 1/2; Attrition bias: high in 1/2 and unclear in 1/2; Performance bias: low in 2/2; Detection bias: low in 1/2 and unclear in 1/2; | | | |
| Consistency: | 0 | No Important inconsistency. One study shows that there is a significant decrease in pain after treatment with oral alendronate. Although treatment with oral alendronate is decreased in the other study, this effect is not considered significant. | | | |
| Directness: | 0 | Results are direct. Outcomes are generalizable. | | | |
| Precision: | -1 | Some imprecisions due to small study of Seikaly, 2005 | | | |
| Publication bias: | 0 | Unlikely | | | |
| Effect size: | 0 | No large magnitude of effect | | | |
| Dose-response: | 0 | Unclear dose-response relationship | | | |
| Plausible confounding: | 0 | No plausible confounding | | | |
| **Quality of evidence:** |  | **⊕⊕⊖⊖ LOW** | | | |
| **Conclusion:** |  | **There is low quality of evidence that treatment with oral alendronate decreases pain in children with Osteogenesis Imperfecta as compared to treatment with placebo (significant in one study).** | | | |

| **Oral alendronate** | | | | | |
| --- | --- | --- | --- | --- | --- |
| Studies | Type of participants | | Total no. of participants | Type of intervention vs control | Outcome and Effect size |
| **Safety,** adverse events and adverse effects | | | | | |
| N = 2 RCTs extracted from systematic review of RCTs: Beecham, 2015.  Included RCTs:   - Seikaly, 2005 (cross-over RCT) - Ward, 2011 | Children with Osteogenesis imperfecta (OI) aged 3 to 19. | | Total participants 159  N = 20 (Seikaly, 2005)  N = 139 (Ward, 2011) | Oral alendronate vs placebo | **Number and type of participants with adverse events (intervention vs control)**   - 2 participants vs 1 participant. This resulted in withdrawal from the study (Ward, 2011)   **Number and type of adverse effects**   - 2 of 20 participants with abdominal discomfort (Seikaly, 2005) - 50% of 139 participants experienced gastrointestinal symptoms. No difference in treatment arm (Ward, 2011)   **Most common adverse effect**  Most common reported effects were gastrointestinal symptoms. |
| **Grade assessment** | | | | | |
| Study design: | +4 | 2 Randomized Controlled Trials (results extracted from systematic review of RCTs: Beecham, 2015) | | | |
| Study limitations | -1 | Some limitations - Selection bias: Low in 1/2 and unclear in 1/2; Attrition bias high in 1/2 and unclear in 1/2; Performance bias low in 2/2; Detection bias: low in 1/2 and unclear in 1/2; | | | |
| Consistency: | 0 | No important inconsistency | | | |
| Directness: | 0 | Results are direct. Outcomes are generalizable. | | | |
| Precision: | -1 | Some imprecisions due to small study of Seikaly, 2005 | | | |
| Publication bias: | 0 | Unlikely | | | |
| Effect size: | 0 | No large magnitude of effect | | | |
| Dose-response: | 0 | Unclear dose-response relationship | | | |
| Plausible confounding: | 0 | No plausible confounding | | | |
| **Quality of evidence:** |  | **⊕⊕⊖⊖ LOW** | | | |
| **Conclusion:** |  | **There is low quality of evidence that adverse effects in both intervention and control groups. Most common adverse effects were gastrointestinal symptoms** | | | |

### Oral risedronate

| **Oral risedronate** | | | | | |
| --- | --- | --- | --- | --- | --- |
| Studies | Type of participants | | Total no. of participants | Type of intervention vs control | Outcome and Effect size |
| **Pain** | | | | | |
| N = 1 RCTs extracted from systematic review of RCTs: Beecham, 2015.  Included RCTs:   - Bishop, 2013 | Children with Osteogenesis imperfecta (OI) | | Total participants unknown | Oral risedronate vs placebo | **Pain was considered an adverse event and was measured using pain scales:**  When pain was reported as an adverse event there was no significant difference between the intervention and control group in the number of participants experiencing pain: OR 1.54,95% CI 0.52 to 4.56 (Bishop, 2013)    No difference in pain scales was measured (discussion of Bishop, 2013) |
| **Grade assessment** | | | | | |
| Study design: | +4 | 1 Randomized Controlled Trial (results extracted from systematic review of RCTs: Beecham, 2015) | | | |
| Study limitations | 0 | No limitations - Selection bias: Low; Attrition bias: low; Performance bias: low; Detection bias: low | | | |
| Consistency: | 0 | No important inconsistency. Only 1 study performed | | | |
| Directness: | 0 | Results are direct. Outcomes are generalizable. | | | |
| Precision: | -2 | Serious imprecision due to unknown sample size. Only 1 study performed | | | |
| Publication bias: | 0 | Unlikely | | | |
| Effect size: | 0 | No large magnitude of effect | | | |
| Dose-response: | 0 | Unclear dose-response relationship | | | |
| Plausible confounding: | 0 | No plausible confounding | | | |
| **Quality of evidence:** |  | **⊕⊕⊖⊖ LOW** | | | |
| **Conclusion:** |  | **There is low quality of evidence there is no significant effect of treatment with oral risedronate on pain in children with Osteogenesis Imperfecta as compared to treatment with placebo.** | | | |

| **Oral risedronate** | | | | | |
| --- | --- | --- | --- | --- | --- |
| Studies | Type of participants | | Total no. of participants | Type of intervention vs control | Outcome and Effect size |
| **Safety,** adverse events and adverse effects | | | | | |
| N = 1 RCTs extracted from systematic review of RCTs: Beecham, 2015.  Included RCTs:   - Bishop, 2013 | Osteogenesis imperfecta (OI) | | Total participants unknown | Oral risedronate vs placebo | **Number of participants with adverse events (intervention vs control)**   - No significant difference in number of adverse events between intervention and control group: OR 0.46, 95% CI 0.09 to 2.24 (Bishop, 2013) |
| **Grade assessment** | | | | | |
| Study design: | +4 | 1 Randomized Controlled Trial (results extracted from systematic review of RCTs: Beecham, 2015) | | | |
| Study limitations | 0 | No limitations - Selection bias: Low; Attrition bias: low; Performance bias: low; Detection bias: low | | | |
| Consistency: | 0 | No important inconsistency. Only 1 study performed | | | |
| Directness: | 0 | Results are direct. Outcomes are generalizable. | | | |
| Precision: | -2 | Serious imprecision due to unknown sample size. Only 1 study performed | | | |
| Publication bias: | 0 | Unlikely | | | |
| Effect size: | 0 | No large magnitude of effect | | | |
| Dose-response: | 0 | Unclear dose-response relationship | | | |
| Plausible confounding: | 0 | No plausible confounding | | | |
| **Quality of evidence:** |  | **⊕⊕⊖⊖ LOW** | | | |
| **Conclusion:** |  | **There is low quality of evidence there is no significant effect of treatment with oral risedronate on adverse events in children with Osteogenesis Imperfecta as compared to treatment with placebo.** | | | |

### Intravenous pamidronate

| **Intravenous pamidronate** | | | | | |
| --- | --- | --- | --- | --- | --- |
| Studies | Type of participants | | Total no. of participants | Type of intervention vs control | Outcome and Effect size |
| **Pain** | | | | | |
| N = 1 RCT extracted from systematic review of RCTs: Beecham, 2015.  Included RCTs:   - Letocha, 2005 | Osteogenesis imperfecta (OI) | | Total participants 18 | Intravenous Pamidronate vs no treatment | - **Pain measured by a 4-point self-reported pain scale (from 4 = no pain to 1 = intractable pain):** No differences in self-reported bone pain were found. Mean difference: -0.11, 95% CI -0.83 to 0.61 (Letocha, 2005) |
| **Grade assessment** | | | | | |
| Study design: | +4 | 1 Randomized Controlled Trial (results extracted from systematic review of RCTs: Beecham, 2015) | | | |
| Study limitations | -2 | Serious limitations - Selection bias: Unclear; Attrition bias: low; Performance bias high; Detection bias: high | | | |
| Consistency: | 0 | No important inconsistency. Only 1 study performed. | | | |
| Directness: | 0 | Results are direct. Outcomes are generalizable. | | | |
| Precision: | -2 | Serious imprecision due to small sample size. Only 1 study performed | | | |
| Publication bias: | 0 | Unlikely | | | |
| Effect size: | 0 | No large magnitude of effect | | | |
| Dose-response: | 0 | Unclear dose-response relationship | | | |
| Plausible confounding: | 0 | No plausible confounding | | | |
| **Quality of evidence:** |  | **⊕⊖⊖⊖ VERY LOW** | | | |
| **Conclusion:** |  | **There is very low quality of evidence there is no significant effect of treatment with intravenous pamidronate on pain in children with Osteogenesis Imperfecta as compared to no treatment.** | | | |

| **Intravenous pamidronate** | | | | | |
| --- | --- | --- | --- | --- | --- |
| Studies | Type of participants | | Total no. of participants (intervention vs control) | Type of intervention vs control | Outcome and Effect size |
| **Safety,** adverse events and adverse effects | | | | | |
| N = 1 RCT extracted from systematic review of RCTs: Beecham, 2015.  Included RCTs:   - Letocha, 2005 | Osteogenesis imperfecta (OI) | | Total participants 18 | Intravenous Pamidronate vs no treatment | All participants experienced acute phase reactions upon the first infusion cycle of pamidronate. What these reactions were are not described; no other complications were noted (Letocha, 2005). |
| **Grade assessment** | | | | | |
| Study design: | +4 | 1 Randomized Controlled Trial (results extracted from systematic review of RCTs: Beecham, 2015) | | | |
| Study limitations | -2 | Serious limitations - Selection bias: Unclear; Attrition bias: low; Performance bias high; Detection bias: high | | | |
| Consistency: | 0 | No important inconsistency. Only 1 study performed. | | | |
| Directness: | 0 | Results are direct. Outcomes are generalizable. | | | |
| Precision: | -2 | Serious imprecision due to small sample size. Only 1 study performed | | | |
| Publication bias: | 0 | Unlikely | | | |
| Effect size: | 0 | No large magnitude of effect | | | |
| Dose-response: | 0 | Unclear dose-response relationship | | | |
| Plausible confounding: | 0 | No plausible confounding | | | |
| **Quality of evidence:** |  | **⊕⊖⊖⊖ VERY LOW** | | | |
| **Conclusion:** |  | **There is very low quality of evidence that treatment with intravenous pamidronate results in acute phase reactions during the first infusion cycle in children with Osteogenesis Imperfecta.** | | | |
